# Supplementary material for: Sacituzumab Govitecan in patients with breast cancer brain metastases and recurrent glioblastoma: a phase 0 window-of-opportunity trial
Source: Nat Commun. 2024 Aug 7;15:6707. doi: 10.1038/s41467-024-50558-9 (PMC11306739; doi:10.1038/s41467-024-50558-9)
Supplement: Supplementary file 1 — Supplementary Information [file 41467_2024_50558_MOESM1_ESM.pdf]

SUPPLEMENTARY TABLES

Supplementary Table 1: Adverse events with incidence greater than 2% (n=25).

	Number of patients n (%)	
Adverse Event	*All grades*	*Grade 3 or Higher*
Other AE not listed	6 (24)	2 (8)
Alopecia	11 (44)	
Anemia	4 (16)	
Constipation	6 (24)	
Cough	5 (20)	
Diarrhea	13 (52)	1 (4)
Dizziness	4 (16)	
Dysuria	5 (20)	
Encephalitis infection		1 (4)
Fatigue	15 (60)	
Febrile neutropenia		1 (4)
Flu like symptoms		1 (4)
Headache	10 (40)	
Hyperglycemia	6 (24)	
Hypertension		1 (4)
Hypertriglyceridemia		1 (4)
Hypokalemia	7 (28)	2 (8)
Insomnia	5 (20)	
Myalgia	4 (16)	
Nausea	9 (36)	
Neutrophil count decreased	10 (40)	7 (28)
Paresthesia		1 (4)
Rash maculo-papular	6 (24)	1 (4)
Seizure	5 (20)	2 (8)
Sepsis		1 (4)
Skin hyperpigmentation	4 (16)	
Skin infection		1 (4)
Thromboembolic event		2 (8)
Urinary tract infection		2 (8)
Urticaria		1 (4)
Vomiting	6 (24)	
Wound infection		1 (4)
White blood cell decreased	4 (16)	
Edema limbs	4 (16)	
Lung infection		1 (4)
Anorectal infection		1 (4)
Soft tissue infection		1 (4)
Muscle weakness lower limb		2 (8)

Table of adverse events including all events with greater than 2% incidence and detailing both all grades and grade 3 or higher.

Supplementary Table 2: Summary of SN-38 levels, Trop-2, γH2AX and CAIX expression in breast cancer with brain metastasis and recurrent glioblastoma samples.

Patient #	Cancer Type	SN-38 Levels in Serum (ng/ml)	SN-38 Levels in Tissue (ng/g)	% SN-38 Levels Tissue/Serum Ratio	SN-38 Levels in CSF (ng/ml)	Trop-2 Expression H-score	γH2AX Expression Quantification (% positive nuclei)	Tissue Hypoxia by CAIX Expression	Comments
1	BCBM	5266.5	102.2	2	N/A	167.2	66.0	High	
5	BCBM	3646.7	182.6	5	N/A	159.2	39.9	Low	
8	BCBM	4473	308.3	7	5	168.6	38.5	High	
10	BCBM	5659.6	539.4	10	N/A	182.6	77.9	High	
12	BCBM	1758.1	372.1	21	N/A	N/A	N/A	N/A	No tissue for IHC
13	BCBM	1743.2	66.9	4	N/A	169.0	33.1	Low	
14	BCBM	2266.8	86.5	4	N/A	134.7	20.2	Low	
20	BCBM	3098	652	21	N/A	185.0	52.7	Low	
21	BCBM	1266	212	17	N/A	224.8	53.8	High	
23	BCBM	97.9	21.2	22	9.4	55.7	73.7	Low	Pathology not consistent with recurrence
24	BCBM	1410	122	9	26.5	243.6	67.3	High	
25	BCBM	2658	236	9	N/A	214.6	67.3	Low	
26	BCBM	1318	118	9	N/A	252.4	81.2	Low	
2	GBM	5363.1	229.2	4	5.1	59.2	78.2	High	
3	GBM	3923.8	211.8	5	N/A	0	67.3	Low	
4	GBM	3918.2	60.4	2	N/A	N/A	85.1	N/A	Radiation injury
6	GBM	3089.4	39.7	1	N/A	6.8	43.4	Low	
7	GBM	4291.9	141.9	3	N/A	140.4	64.1	Low	
9	GBM	2465.7	46.5	2	N/A	8.0	91.8	Low	
15	GBM	1810.4	259.1	14	N/A	293.3	65.1	Low	
16	GBM	N/A	N/A	N/A	N/A	40.0	78.3	High	No sample for SN-38 quantification
17	GBM	2221.3	232.2	10	N/A	N/A	N/A	N/A	No tissue for IHC
18	GBM	1930.9	81.6	4	N/A	N/A	71.7	N/A	Radiation injury, no evidence of high-grade glioma
19	GBM	2226.5	104.5	5	N/A	111.2	80.4	High	
22	GBM	115	8.6	7	N/A	N/A	84.2	N/A	Radiation injury

Table of correlatory biomarkers, detailing SN-38 levels in serum, tissue and CSF as well H-Score. Expression of γH2AX and CAIX for these patients is also shown.

SUPPLEMENTARY FIGURES

Supplementary Figure 1: Quantification of tumor volume in mice.

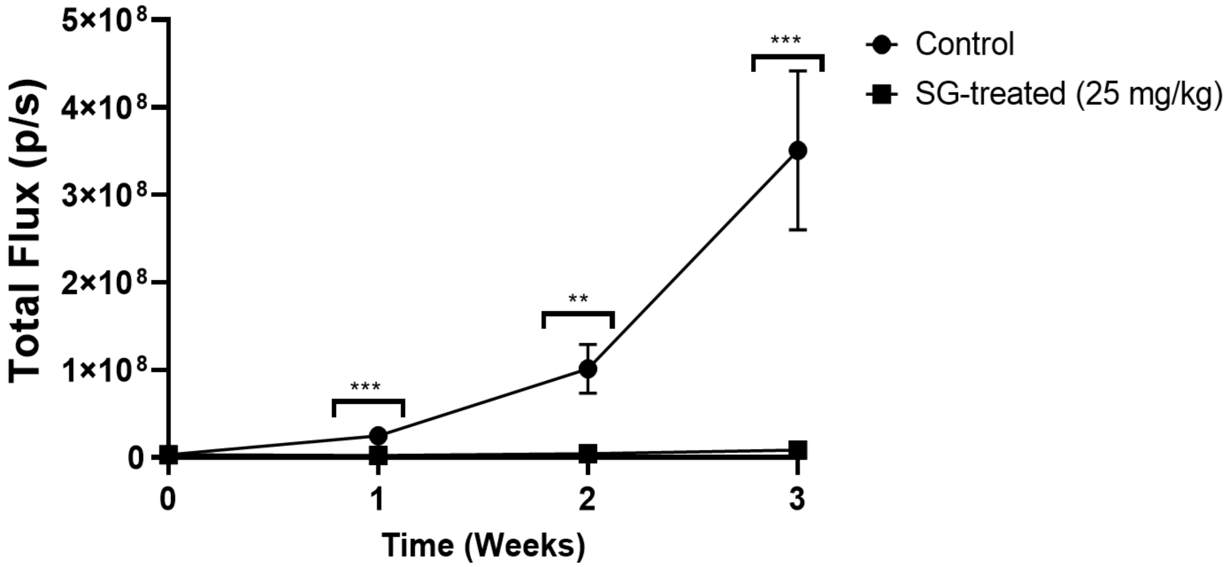

SCID/NCr Mice (n=20) treated with sacituzumab govitecan (SG) were imaged using a Xenogen in vivo imaging system to measure total flux in pixels/second (p/s). Statistical differences were calculated using two-sided unpaired t-test (p-value = 0.00004 at week 1; 0.004 at week 2; 0.0007 at week 3).

Supplementary Figure 2: Sacituzumab govitecan inhibits tumor growth in a xenograft model of intracranial breast cancer.

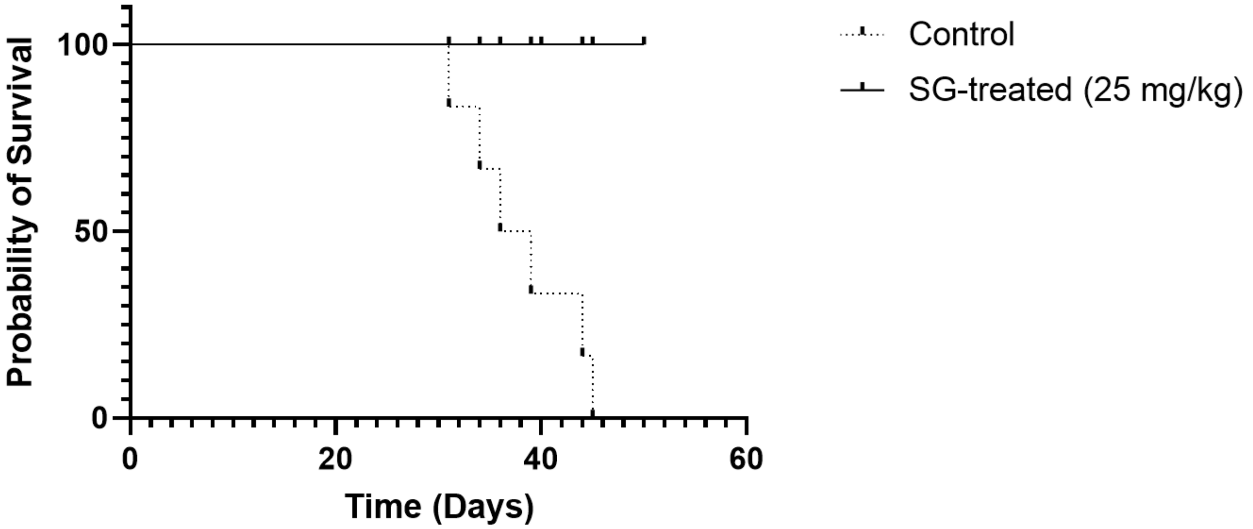

Kaplan-Meier survival curve comparing mice treated with sacituzumab govitecan (SG) and control vehicle (saline). (Control n=10; SG n=10). P-value was calculated using the Log-rank (Mantel-Cox) test (p-value = 0.0022).

Supplementary Figure 3: CONSORT flow diagram of participants.

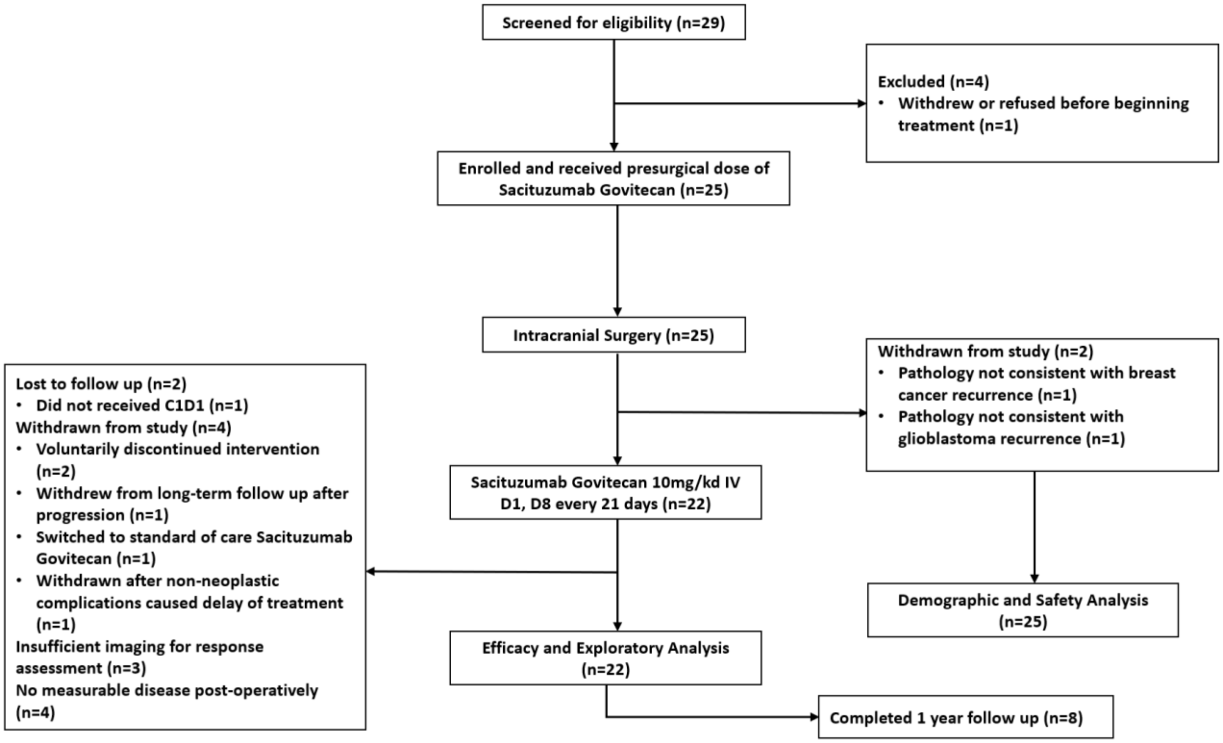

Diagram of patient disposition and treatment.

Supplementary Figure 4: Progression-free survival for patients having breast cancer with brain metastasis after treatment with sacituzumab govitecan.

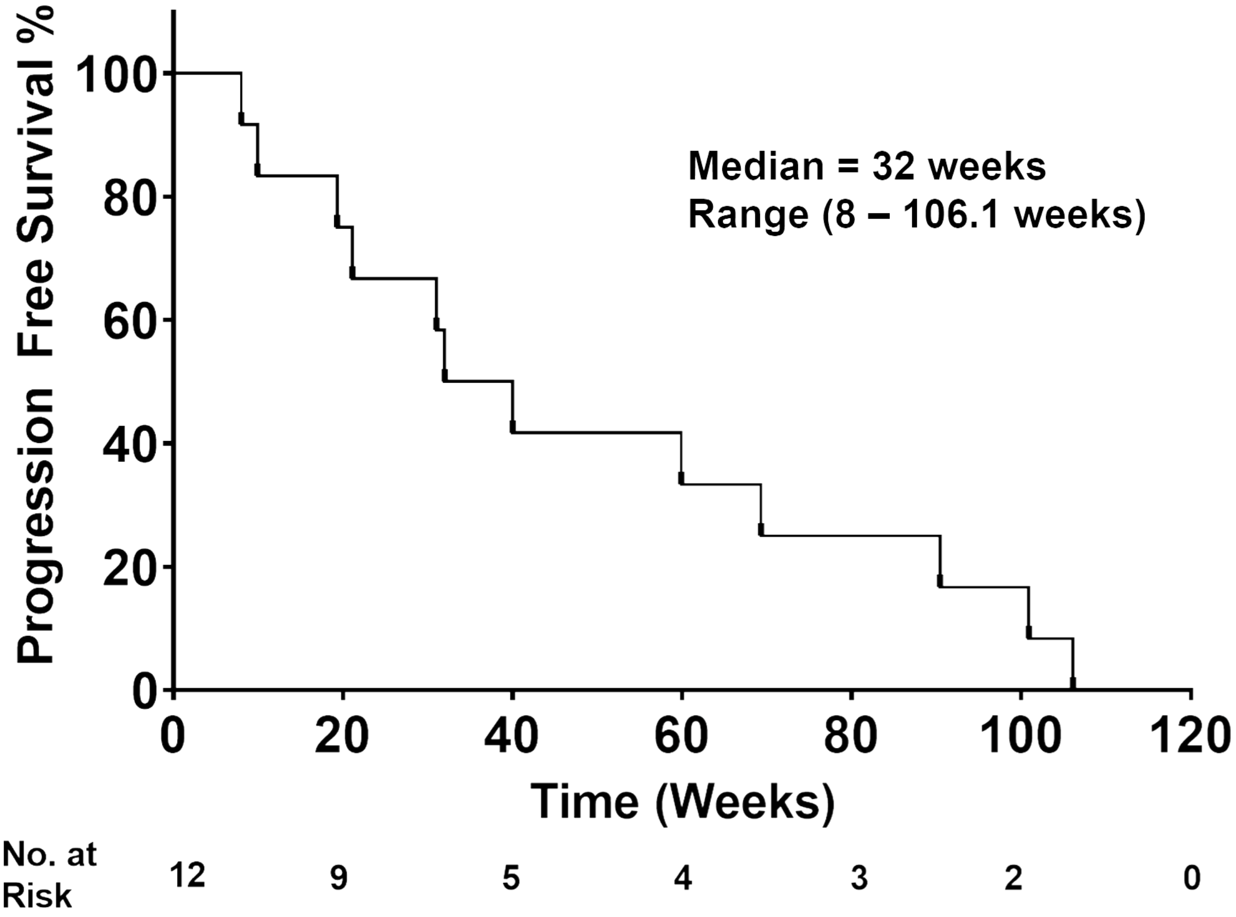

Kaplan-Meier plot showing progression free survival for patients (n = 12). Median was 32 weeks, and the range was 8 to 106.1 weeks.

Supplementary Figure 5: Progression-free survival for patients with recurrent glioblastoma after treatment with sacituzumab govitecan.

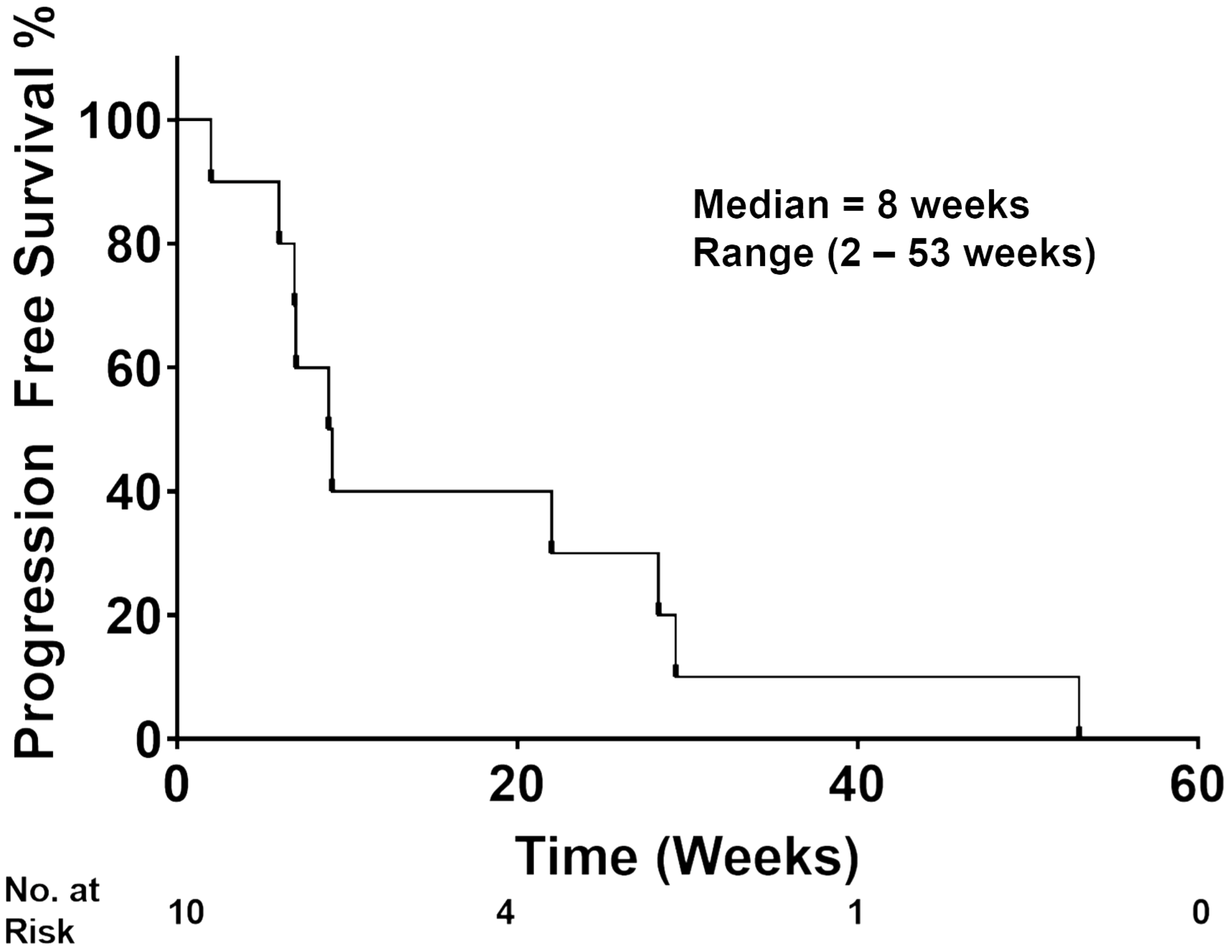

Kaplan-Meier plot showing progression free survival for patients (n = 10). Median was 8 weeks, and the range was 2 to 53 weeks.

Supplementary Figure 6: Intracranial disease response for patients having breast cancer with brain metastasis after treatment with sacituzumab govitecan.

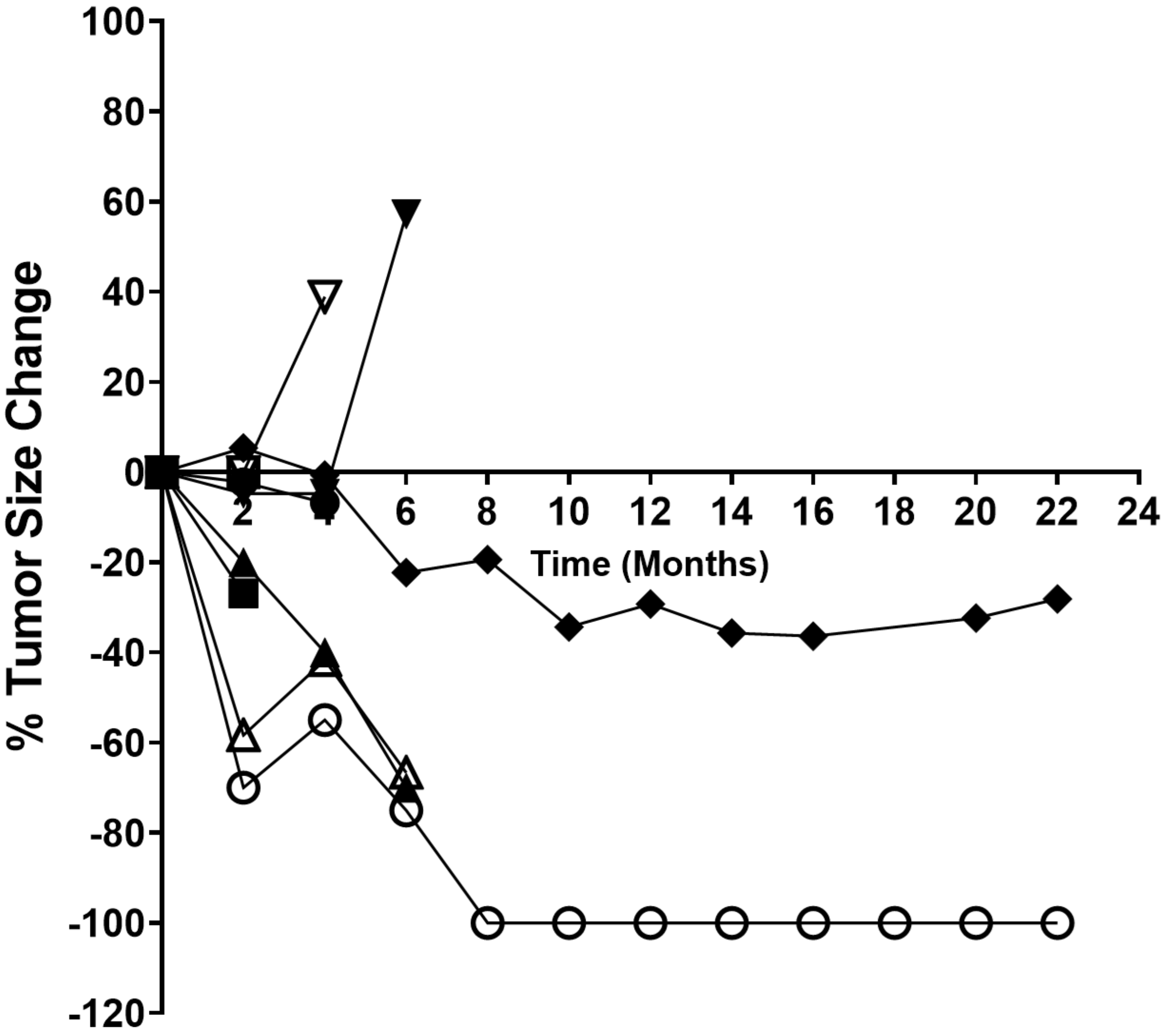

Spider plot showing intracranial best disease response as per RECIST 1.1 and duration of response (n = 9).

Supplementary Figure 7: Patient #20.

1a 1b 1c 1d

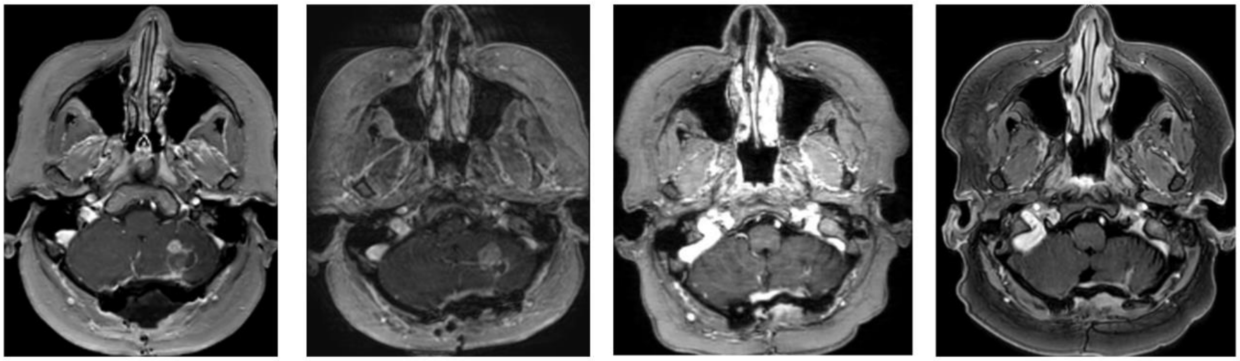

Patient #20 (breast cancer with brain metastasis) had had left cerebellar and right frontal lesions on MRI (Day -88, 1A) and underwent left cerebellar resection (Day -63). Enrolled on Sacituzumab and underwent right frontal resection (Day 1). MRI (Day +29, 1B) with post-surgical changes and residual nodular enhancement. MRI (Day +302, 1C) shows near complete resolution of nodularity. MRI (Day +770, 1D) eventually showed progression and patient came off study and was treated with radiosurgery. At time of publication (Day +1299) patient remains alive and on standard-of-care Sacituzumab.

Supplementary Figure 8: Extracranial disease response for patients having breast cancer with brain metastasis after treatment with sacituzumab govitecan.

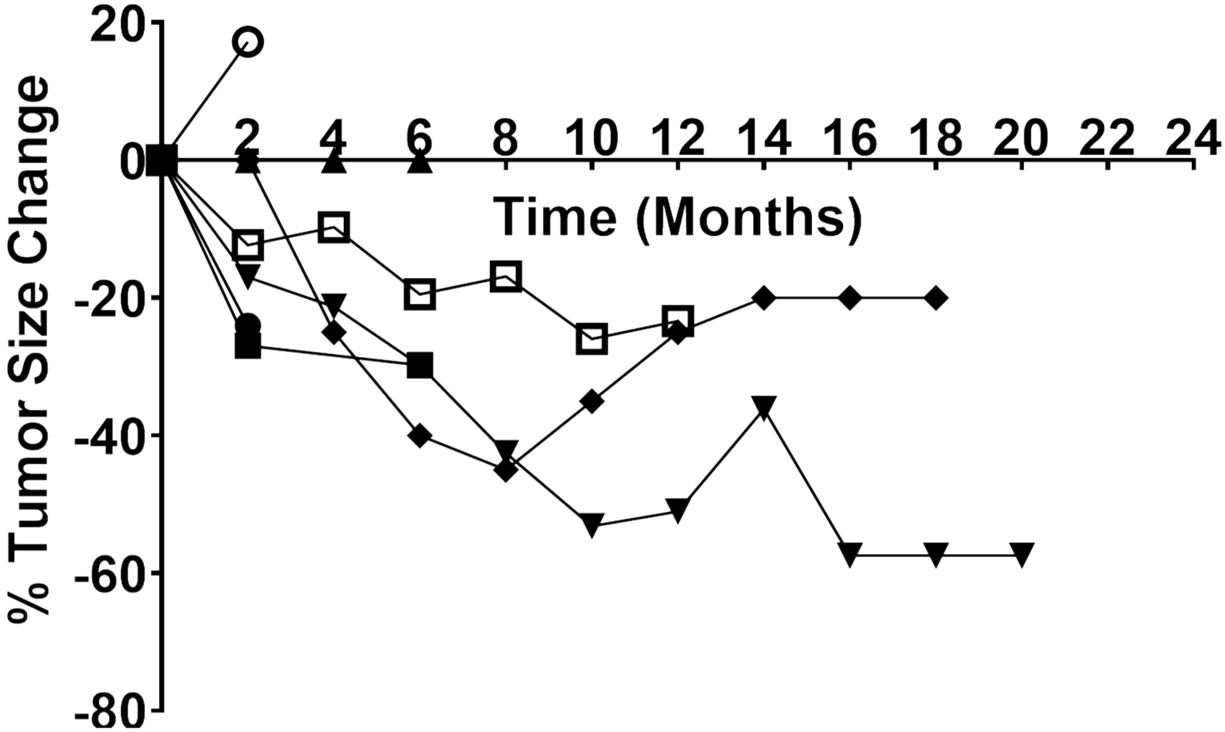

Spider plot showing extracranial disease response as per RECIST 1.1 and duration of response (n= 7).

Supplementary Figure 9: Intracranial disease response for patients with recurrent glioblastoma after treatment with sacituzumab govitecan.

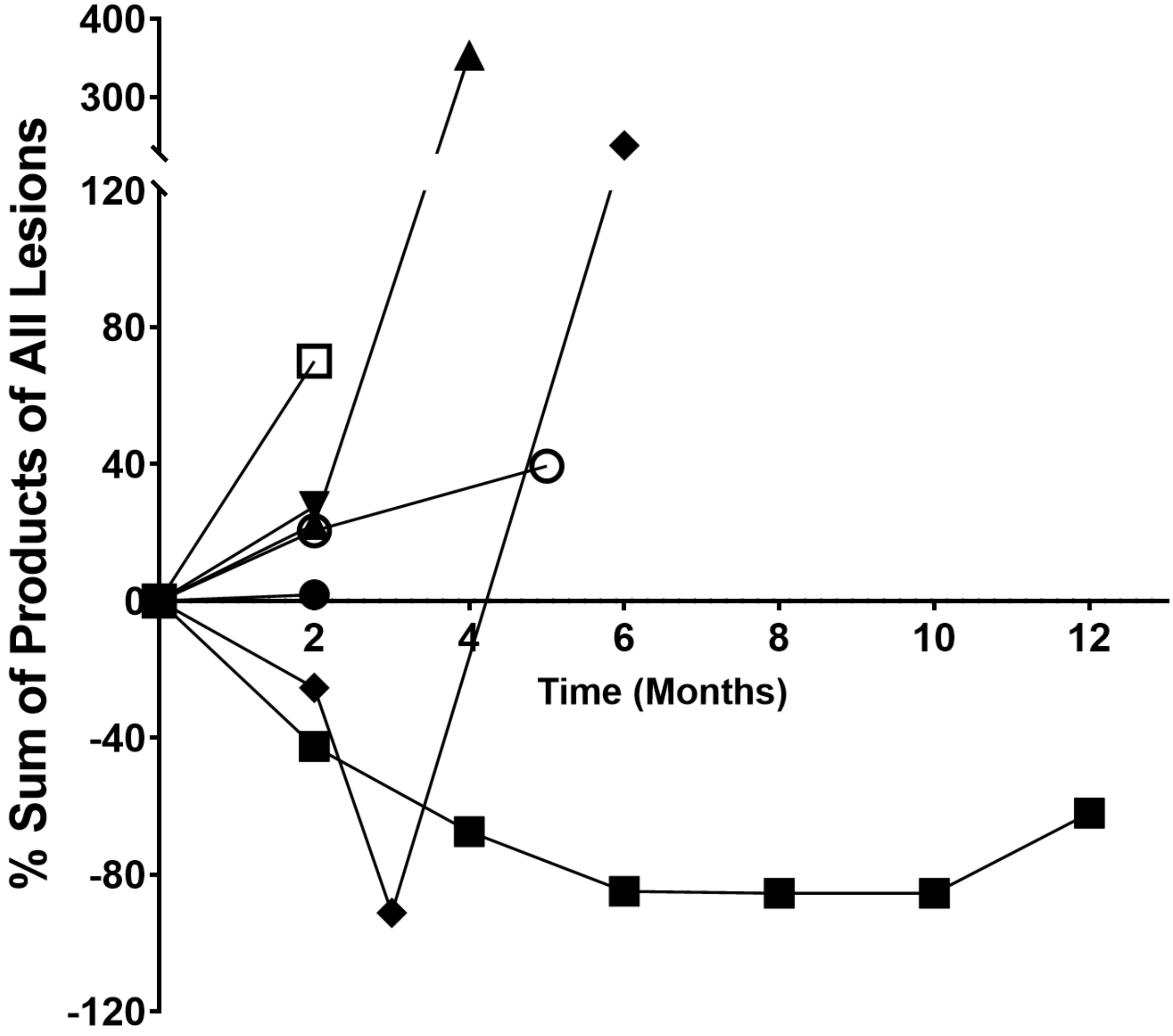

Spider plot showing intracranial disease response as per RANO criteria (n = 7).

Supplementary Figure 10: Patient #9.

2a 2b 2c 2d

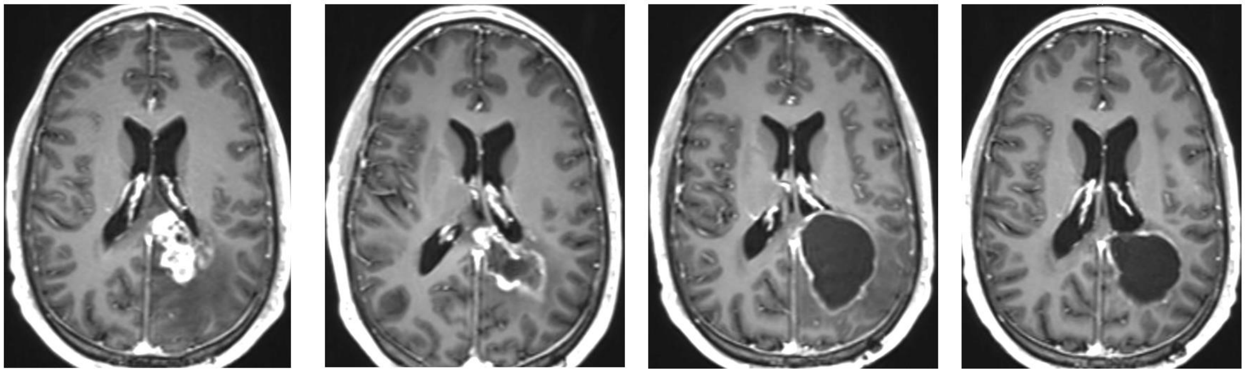

Patient #9 (recurrent glioblastoma) completed chemoradiation (Day -197). Unfortunately, MRI (Day -70) showed interval increase in medial left hemisphere mass. Presurgical MRI (Day -3, 2A) again demonstrated this left parietal mass crossing the midline as well as multicentric medial right occipital lesions and a non-enhancing right posterior temporal lobe lesion. Underwent surgery (Day 1). However, MRI (Day +40, 2B) showed residual enhancement along splenium and inferior aspect of surgical cavity. Had partial response (Day +147) and preserved on MRI (Day +222) but eventually progressed (Day +313).

Supplementary Figure 11: Trop-2 and CAIX expression in patients having breast cancer with brain metastasis.

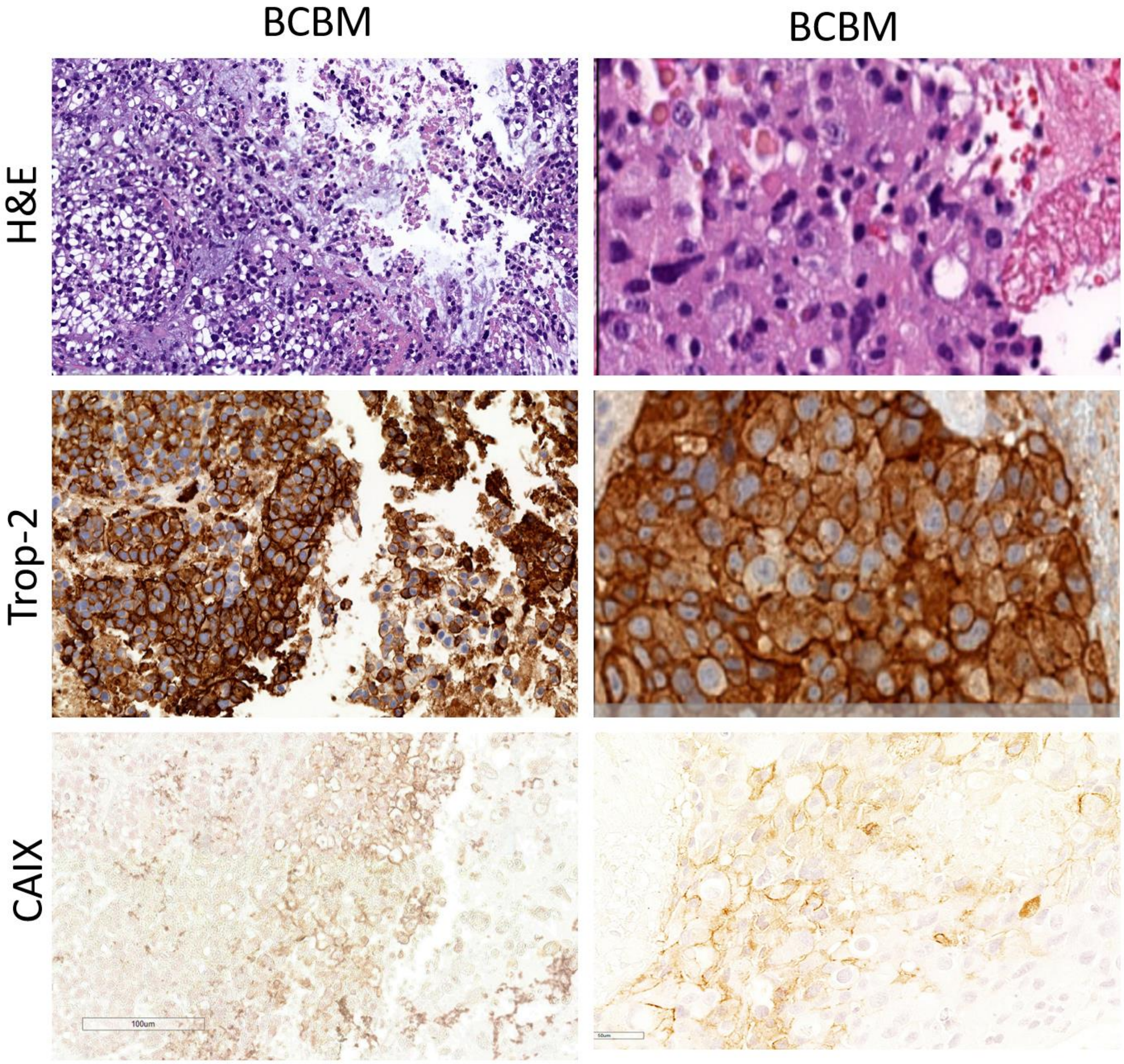

Representative images for hematoxylin and eosin (H&E), Trop-2 and CAIX immunohistochemistry staining in patients having breast cancer with brain metastasis (BCBM).

Supplementary Figure 12: Trop-2 and CAIX expression in patients with recurrent glioblastoma.

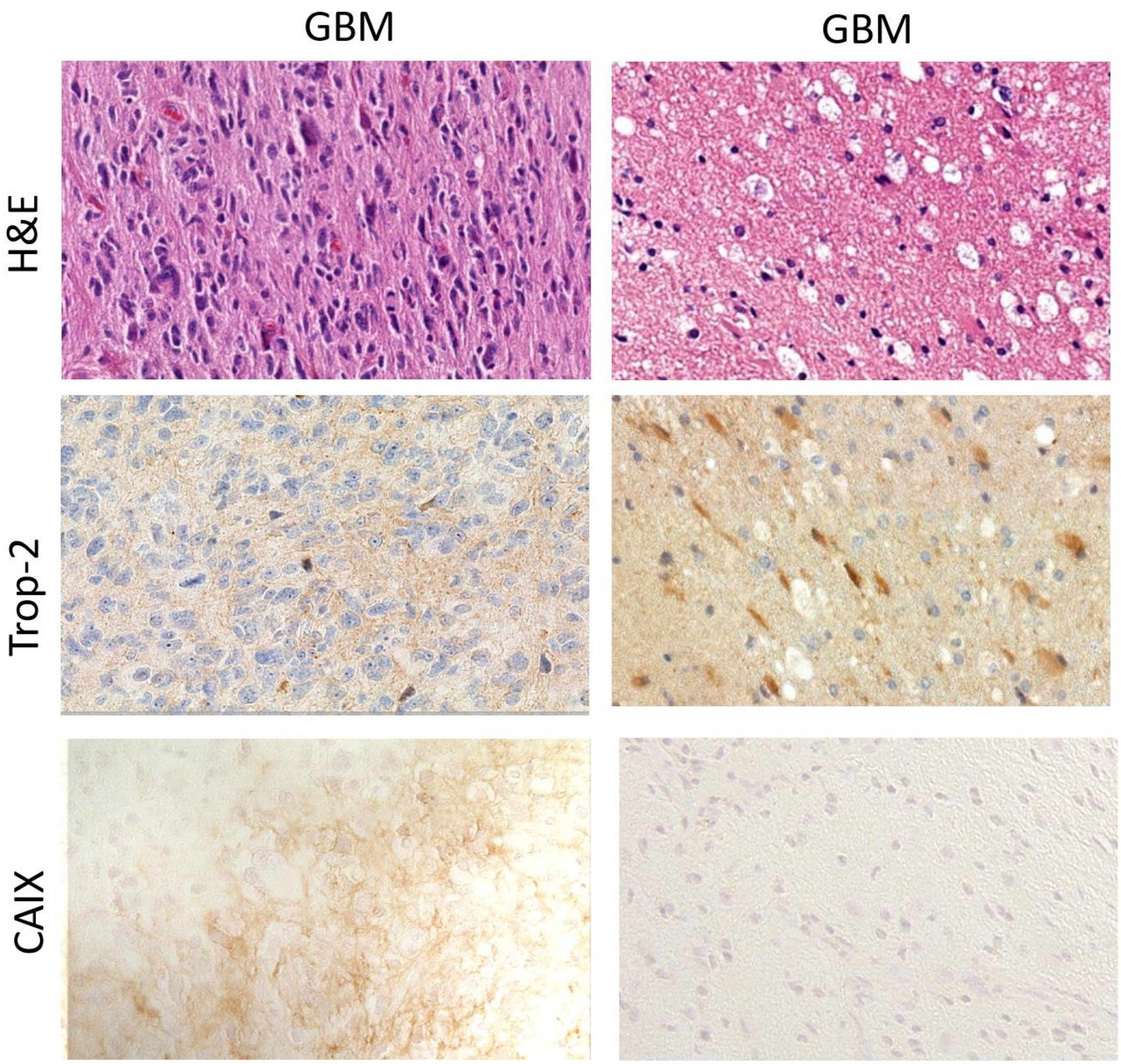

Representative images for hematoxylin and eosin (H&E), Trop-2 and CAIX immunohistochemistry staining in patients with recurrent glioblastoma (GBM).

Supplementary Figure 13: Correlation between SN-38 tissue/serum drug ratio and Trop-2 expression levels in patients having breast cancer with brain metastasis.

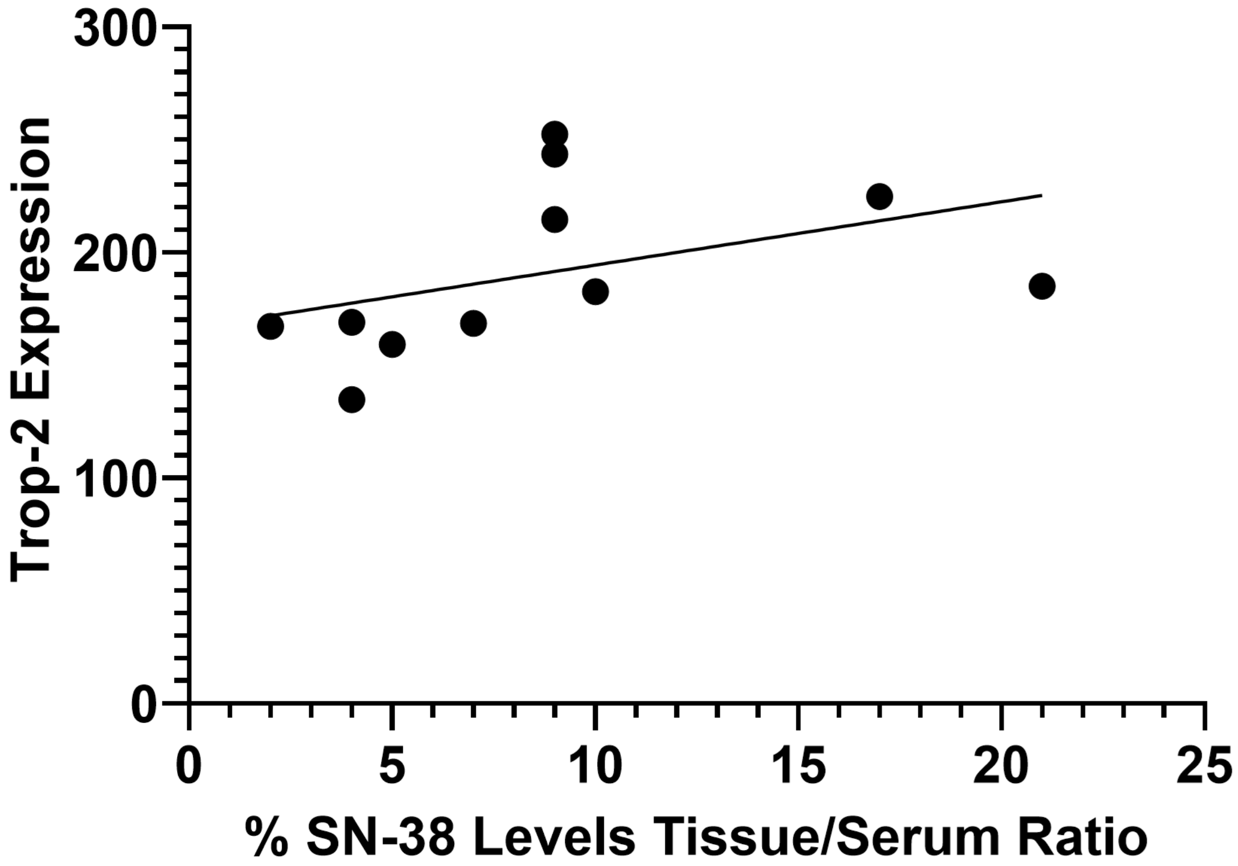

Linear regression was used to determine correlation between SN-38 tissue/serum ratio and Trop-2 expression levels (r^2 = 0.18).

Supplementary Figure 14: Correlation between SN-38 tissue/serum drug ratio and Trop-2 expression levels in patients with recurrent glioblastoma.

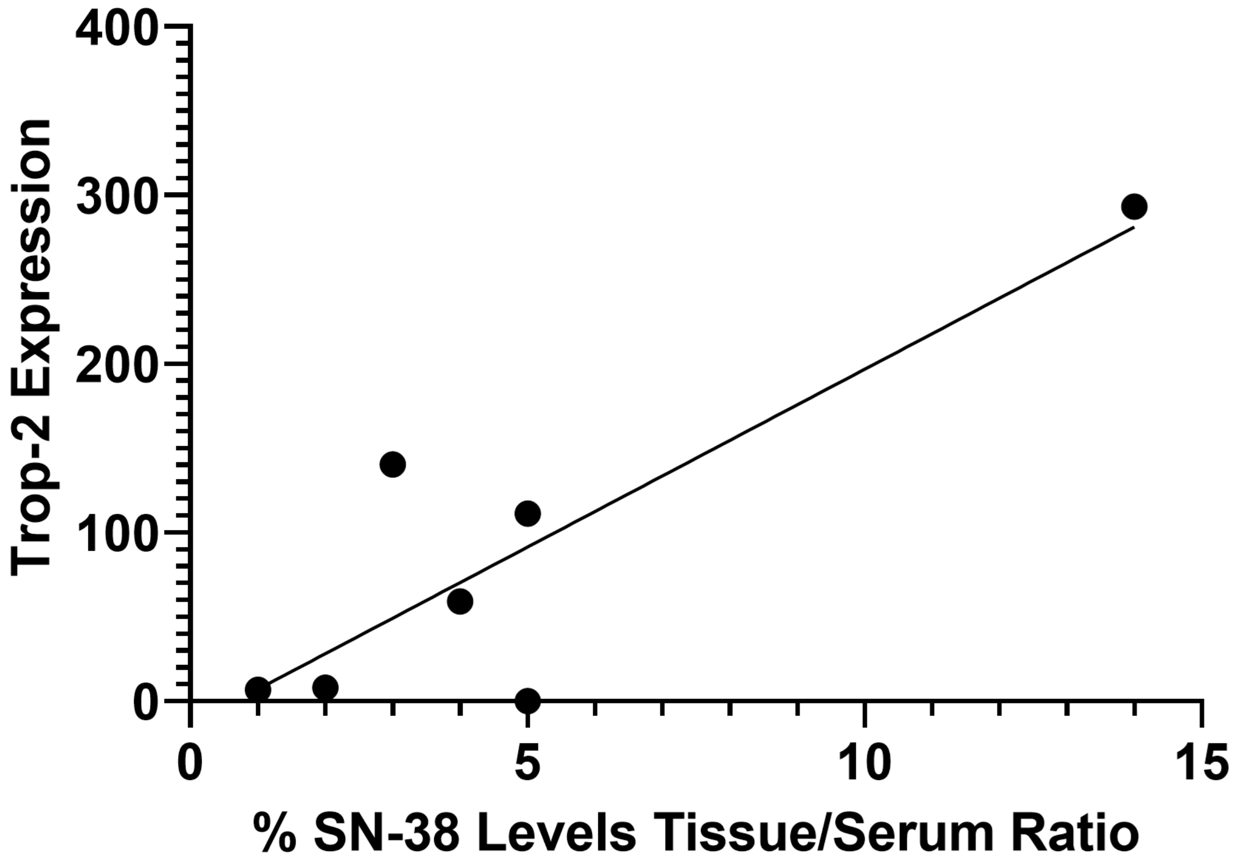

Linear regression was used to determine correlation between SN-38 tissue/serum ratio and Trop-2 expression levels (r^2 = 0.74).

Supplementary Figure 15: Correlation between SN-38 tissue/serum drug ratio and γH2AX expression levels in patients having breast cancer with brain metastasis.

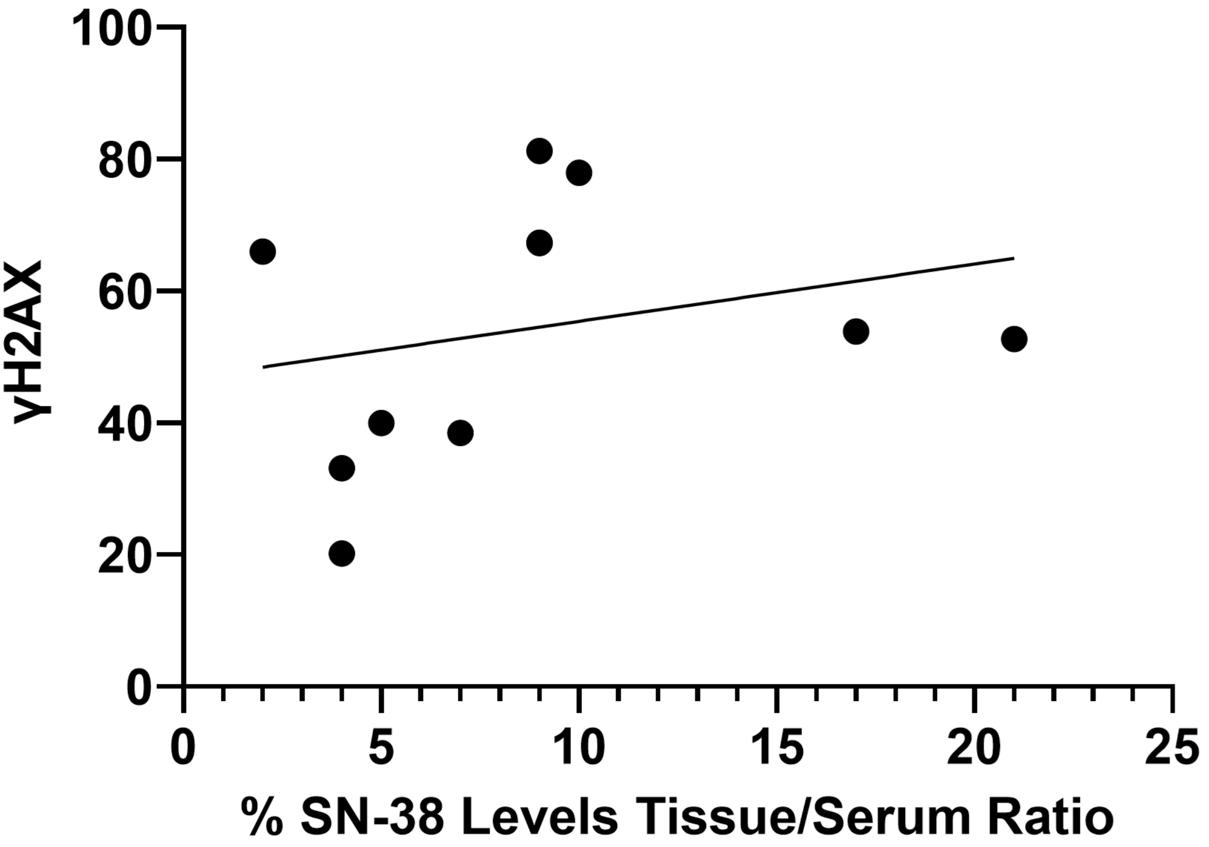

Linear regression was used to determine correlation between SN-38 tissue/serum ratio and γH2AX expression levels (r^2 = 0.06).

Supplementary Figure 16: Correlation between SN-38 tissue/serum drug ratio and γH2AX expression levels in patients with recurrent glioblastoma.

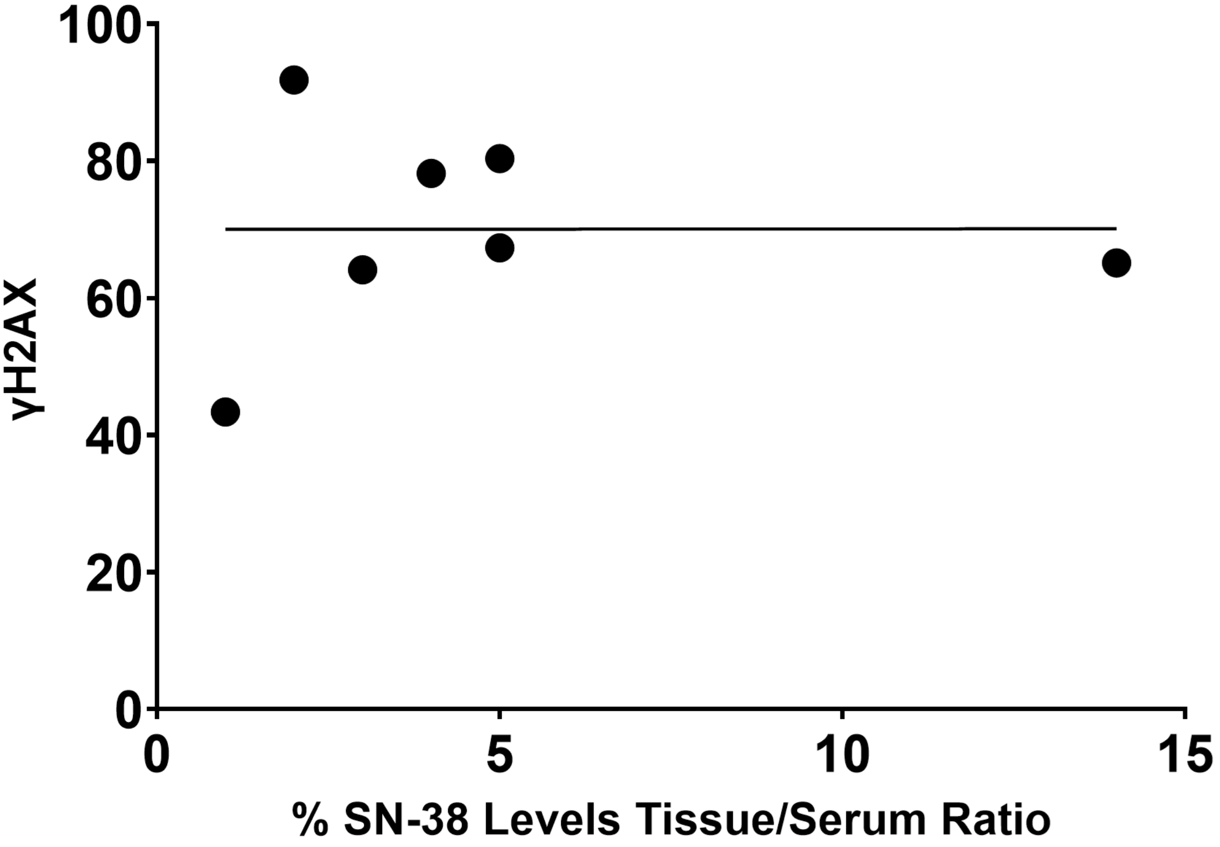

Linear regression was used to determine correlation between SN-38 tissue/serum ratio and γH2AX expression levels ($r^2 = 3.7x10^{-6}$).

Supplementary Figure 17: Comparison of SN-38 tissue/serum ratio in patients with high and low CAIX expression in patients having breast cancer with brain metastasis.

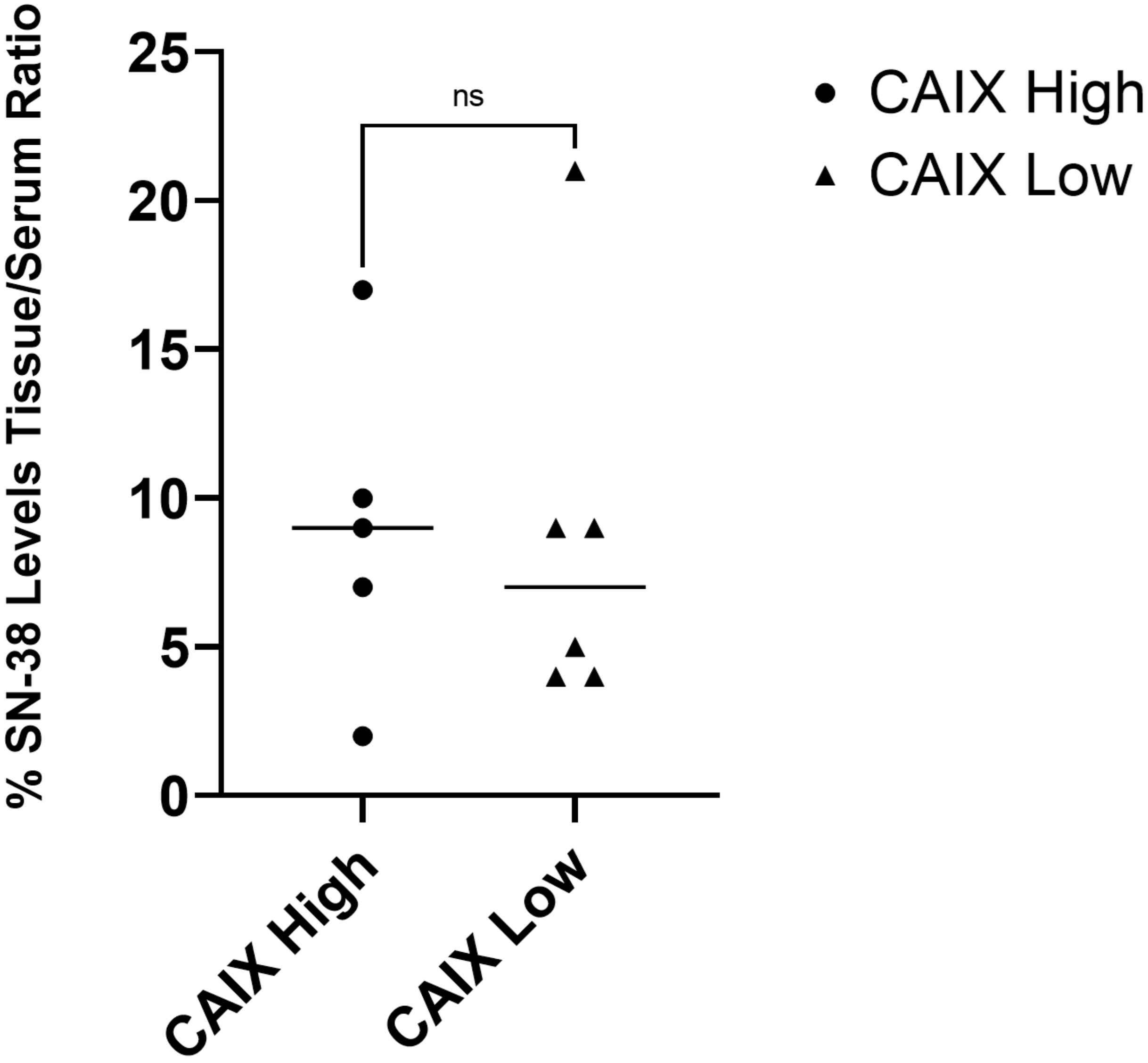

A two-tailed t test was used to compare SN-38 tissue/serum drug ratio levels between patients with high and low CAIX expression (p-value = 0.93).

Supplementary Figure 18: Comparison of SN-38 tissue/serum ratio in patients with high and low CAIX expression in patients with recurrent glioblastoma.

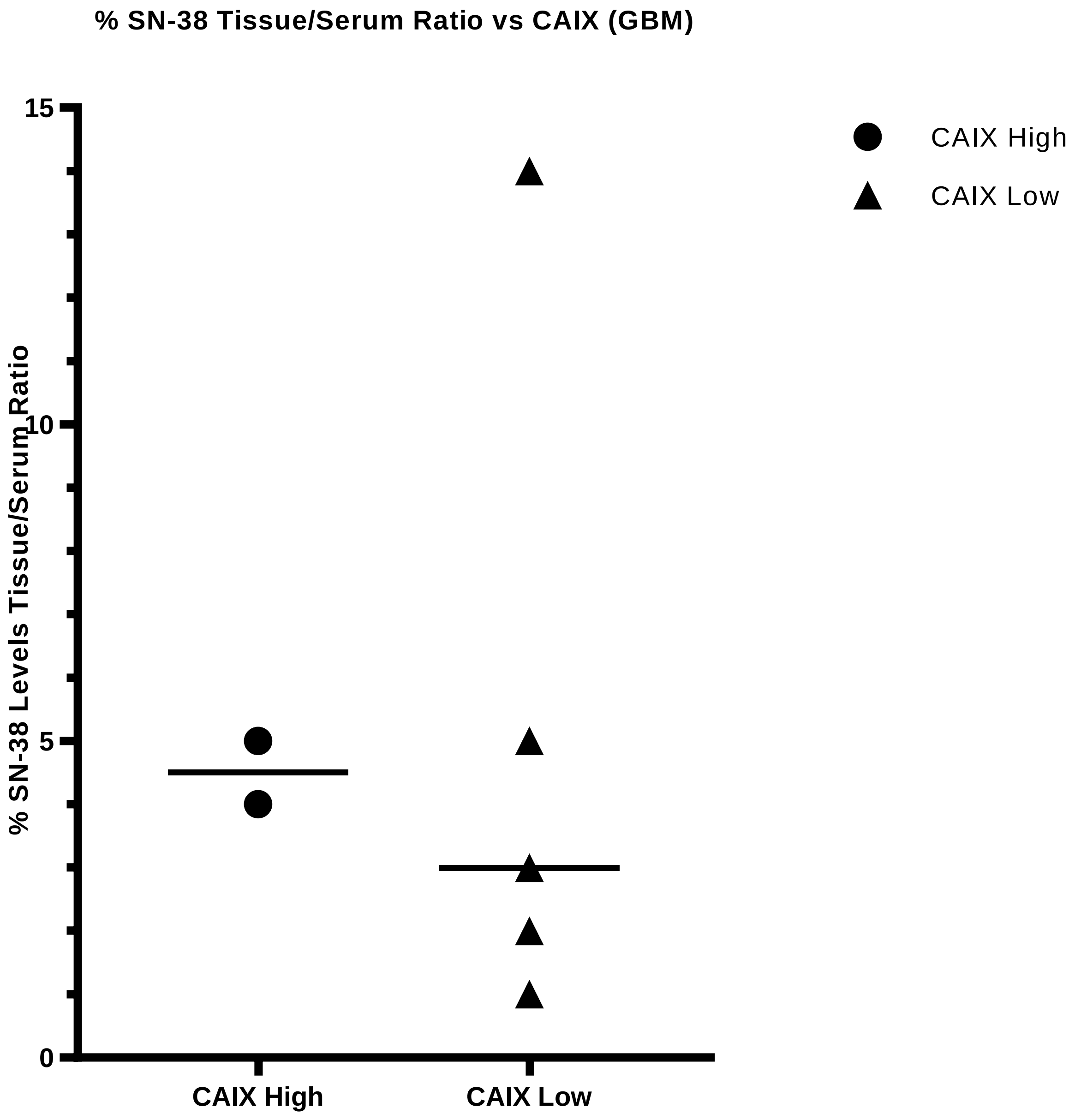

Comparison of SN-38 tissue/serum ratio in patients with high and low CAIX expression in patients with recurrent glioblastoma (p-value =0.9).

SUPPLEMENTARY NOTE 1

Demographics Supplementary: Gender assignment was by self-report and no post hoc analysis of gender was conducted. No formal subgroup analysis by sex or gender was conducted due to limited sample size. At time of publication one patient on the BCBM remains on active follow up. One of the 4 screened patients (#11) had signed consent but then opted for treatment elsewhere. For the BCBM patient (#23) whose surgical tissue was not consistent with disease recurrence, the intracranial biopsy had shown no malignant cells and was notable for fragments of benign cortical brain parenchyma and reactive gliosis compatible with radiation effect. This patient was taken off study but their data is included in the demographic, safety and CSF analysis but not the efficacy nor exploratory analysis. As this was a perioperative study, some BCBM patients (#10, 13, 24, 25) had no measurable disease post-operatively and therefore were not included in response assessments. Of the 8 BCBM patients included in the radiographic response rate analysis (#1, 5, 8, 12, 14, 20, 21, 26), all had active brain metastasis at time of study enrollment. 2 BCBM patients (#1, 21) had had prior intracranial radiation including whole brain radiation for both, with the interval between last radiation and surgical resection on study being 273 and 230 days, respectively. One patient (#14, BCBM) received 26 cycles of Sacituzumab on study and was then switched to standard of care Sacituzumab. One patient (#26, BCBM) voluntarily withdrew from the study after 18 cycles of Sacituzumab but consented to ongoing prospective data analysis. For the rGBM cohort, patient #16 never received C1D1 and was therefore excluded from ORR, PFS and OS. Another patient (#18) was determined to have radiation necrosis at surgery and did not receive post-surgical treatment. This patient was included in demographic and safety but not efficacy nor the exploratory analysis. Another patient in the rGBM cohort (#4) also had pathology showing fibrosis, atypical gliosis, fibrin and macrophages, favoring radiation necrosis. This patient elected to continue with the study and is included in all analysis. A third rGBM patient had elements of rGBM and radiation necrosis and is also included in all analysis. Two rGBM patients (#2, #17) had insufficient postop imaging for RANO assessment. A third patient (#19) had clinical progression after C1D8 but was lost to follow up before post-surgical imaging could be obtained. Patient #2, #17 and #19 were therefore excluded for ORR but included for OS and PFS. At the time of data cutoff for publication of this study, 1 patient remained on alive and on study treatment

with 2 other patients alive and on long-term follow up. Two rGBM patients (#3, #4) had PD on final assessment for new lesions. Another had a 'new' lesion which did not meet size criteria and therefore still qualified as PR. However, the patient voluntarily withdrew form study shortly after. It should also be noted that this study took place during height of the COVID-19 epidemic which may have impacted logistics.

SUPPLEMENTARY NOTE 2

A Phase 0, Investigator Initiated Study to Determine the Bioavailability of Sacituzumab Govitecan in Breast Brain Metastasis and Glioblastoma

Study Center:
Mays Cancer Center at UT Health San Antonio
Institute for Drug Development
7979 Wurzbach Rd., 4th Floor Zeller Building
San Antonio, TX 78229

Principal Investigator:
Andrew Brenner, MD, PhD
Mays Cancer Center at UT Health San Antonio
Institute for Drug Development
7979 Wurzbach Road, 4th floor
Mail Code# 8232
San Antonio, TX 78229
Tel. (210) 450-5936
Fax. (210) 692-7502
E-mail: brennera@uthscsa.edu

Co-Principal Investigator:
John R. Floyd, MD
UTHSA
Department of Neurosurgery
7703 Floyd Curl Drive Mail
Code# 7843
San Antonio, TX 78229
Tel. (210) 567-5625
Fax. (210) 567-6066
E-mail: floydj@uthscsa.edu

Sub-Investigators: Prathibha Surapaneni
PGY-5, Fellow
Department of Hematology Oncology
UT Health San Antonio

See FDA 1572

IND# **144440**
CTMS# **19-0069**

Protocol Version 6, dated March 10, 2021

Responsible Study Coordinator or Research Nurse: **Christine Maglaki, RN**
(210) 450- 5958
maglaki@uthscsa.edu

Pharmacy: IDS Pharmacy
Britni Secor, PharmD
(210) 450-5885
secor@uthscsa.edu

Regulatory Office: Lisa Creighton, MBA, CCRP
Mays Cancer Center at UT Health San Antonio Institute for Drug Development
7979 Wurzbach Road, 4th floor
Mail Code# 8032
San Antonio, TX 78229
Tel. (210) 450-5953
E-mail: creighton@uthscsa.edu
E-mail: RegulatoryAffairs@uthscsa.edu

INVESTIGATOR'S AGREEMENT

I have read and understand the contents of this clinical protocol for Protocol "A Phase 0, Investigator
Initiated Study to Determine the Bioavailability of Sacituzumab Govitecan in Breast Brain Metastasis and Glioblastoma" CTMS# 19-0069 and will adhere to the study requirements as presented, including all statements regarding confidentially. In addition, I will conduct the study in accordance with current international conference on harmonization (ICH) guidance, Good Clinical Practice (GCP) guidance, the Declaration of Helsinki, US Food and Drug Administration (FDA) regulations and local IRB and legal requirements.

Name of Investigator: Andrew Brenner, MD, PhD

Institution: Mays Cancer Center, UT Health San Antonio

__________________________ _________________

Investigator Signature Date

TABLE OF CONTENTS

Synopsis	5
Introduction	8
Rationale	14
Objectives	14
Study Design	15
Population	22
Procedures	23
Materials and Supplies	28
Management of Intercurrent Events	30
Removing Subjects From Study	34
Conditions for Initiating, Modifying, or Terminating the Study	35
Investigator Responsibilities	36
References	43
Appendix A, Schedule	46
Appendix B, ECOG Performance Scale	48
Appendix C, RANO Response Criteria	48
Appendix D, Immunomedics “Product Inspection Form”	49
Appendix E, Summary of Changes	50

SYNOPSIS	
**Study Title:**	A Phase 0, Investigator Initiated Study to determine he bioavailability of Sacituzumab Govitecan in Breast Brain Metastasis and Glioblastoma
**Investigators:**	Andrew J. Brenner MD, PhD<br>John R. Floyd, MD<br>*See 1572 for other investigators*
**Indication**	Brain metastases from Breast cancer and Primary brain tumors (Glioblastoma)
**Primary Objectives:**	To determine the extent by which Sacituzumab Govitecan is able to penetrate the blood brain barrier and access tumor tissue by testing for free SN-38, SN38G and total SN-38 concentrations in tumor tissue as well as in CSF (if available) and serum samples.
**Secondary Objectives:**	• To determine the progression-free survival after debulking craniotomy for patients treated with Sacituzumab Govitecan in patients with Breast brain metastatic tumors and Glioblastoma.<br>• To determine overall survival after debulking craniotomy for patients treated with Sacituzumab Govitecan in patients with Breast brain metastatic tumors and Glioblastoma.<br>• To assess the safety of Sacituzumab Govitecan in Breast brain metastatic tumors and Glioblastoma.
**Exploratory Objectives:**	Correlate total SN-38 and free SN-38 tumor tissue concentrations with tumoral hypoxia and tumoral Trop-2 expression.
**Study Design:**	Single center, non-randomized, Phase 0 study. Sacituzumab Govitecan given preoperatively, followed by craniotomy with surgery or biopsy of brain tumors (GBM and metastatic brain tumors from Breast) and intraoperative tissue collection will follow with contemporaneous CSF (depending on tumor location) and whole blood (serum) sampling.<br><br>Sacituzumab Govitecan treatment will be initiated on day-1, prior to surgery.<br>Sacituzumab govitecan will continue to be administered by IV infusion on Days 1 and 8 of a 21 day cycle post-operatively until progression. The first infusion will be given over 3 hours. Subsequent infusions may be given over 1-2 hours if previous infusions were well tolerated.

All patients will be subjected to standard dose of Sacituzumab Govitecan 10mg/kg without any dose escalations.

Cohorts	# of subjects	Post-operative Sacituzumab dose	Schedule/ Frequency	Route
A (Breast brain	20	10 mg/kg	D1 and D8 of	Intravenous

<table>
<tr><td></td><td>
<table>
<tr><td>metastasis)</td><td></td><td></td><td>21-day cycle</td><td></td></tr>
<tr><td>B(Glioblastoma)</td><td>10</td><td>10 mg/kg</td><td>D1 and D8 of 21-day cycle</td><td>Intravenous</td></tr>
</table>
</td></tr>
<tr><td>Duration:</td><td>Subjects will be allowed to continue treatment on study post-surgery or until they have evidence of significant treatment-related toxicity or progressive disease.</td></tr>
<tr><td>Planned Total Sample Size:</td><td>Up to approximately 30 subjects: Two cohorts of patients will be enrolled in this exploratory study, 20 subjects with breast brain metastasis and 10 subjects with Glioblastoma.

The study will allow for replacement of subjects if their pathology from the craniotomy surgery on study comes back showing necrosis to achieve the planned total sample size.</td></tr>
</table>

**Inclusion Criteria**	1. At least 18 years of age<br>2. Histologically or cytologically documented breast cancer of all subtypes including hormone receptor positive, HER2 positive, and triple negative (Cohort A) with known or suspected parenchymal brain metastases.<br>3. Recurrent glioblastoma (Cohort B) with documented progression by RANO criteria following standard combined modality treatment with radiation and temozolomide.<br>4. Plans to undergo craniotomy as part of standard of care. Patients emergently needing surgical debulking due to symptoms of their disease are not eligible. 5. Recovered from toxicities of prior therapy to grade 0 or 1<br>ECOG performance status ≤ 2. 6.<br>Life expectancy of at least 3 months. 7.<br>Acceptable liver function: 8.<br>a) Bilirubin ≤ 1.5 times upper limit of normal<br>b) AST (SGOT) and ALT (SGPT) ≤ 3.0 times upper limit of normal (ULN);<br>9. Adequate renal function: calculated creatinine clearance ≥30mL/minute according to the Cockcroft and Gault formula<br>10. Acceptable hematologic status (without hematologic support)<br>a) ANC ≥1500 cells/uL<br>b) Platelet count ≥100,000/uL<br>c) Hemoglobin ≥9.0 g/dL<br>11. All women of childbearing potential must have a negative serum pregnancy test and male and female subjects must agree to use effective means of contraception (surgical sterilization or the use or barrier contraception with either a condom or diaphragm in conjunction with spermicidal gel or an IUD) with their partner from entry into the study through 6 months after the last dose.

**Exclusion Criteria:**	1. The subject is receiving warfarin (or other coumarin derivatives) and is unable to switch to low molecular weight heparin (LMWH) before the first dose of study drug.<br>2. The subject has evidence of acute intracranial or intratumoral hemorrhage either by MRI or computerized tomography (CT) scan. Subjects with resolving hemorrhage changes, punctate hemorrhage, or hemosiderin are eligible.<br>3. The subject is unable to undergo MRI scan (eg, has pacemaker).<br>4. The subject has received enzyme-inducing anti-epileptic agents within 14 days of study drug (eg, carbamazepine, phenytoin, phenobarbital, primidone).<br>5. Patients whose only lesion undergoing resection has received stereotactic radiation within the past 3 months<br>6. The subject has received any of the following prior anticancer therapy:<br>• Biologic agents (antibodies, immune modulators, vaccines, cytokines) within 21 days prior to first dose of study drug • Prior treatment with Sacituzumab Govitecan<br>7. Patients receiving UGT1A1 (Uridine diphosphate glucuronosyl transferase 1A1) inhibitors or inducers.<br>8. History of significant cardiovascular disease, defined as:<br>• Congestive heart failure greater than New York Heart Association (NYHA) Class II according to the NYHA Functional Classification.<br>• Unstable angina or myocardial infarction within 6 months before enrollment.<br>• Serious cardiac arrhythmia.<br>9. Clinically significant ECG abnormality, including:<br>• Marked Baseline prolonged QT/QTc interval (ie, a repeated demonstration of a QTc interval >500 ms) demonstrated on ECG at Screening.<br>• History of risk factors for torsade de pointes (eg, heart failure, hypokalemia, family history of long QT Syndrome).<br>10. Any medical or other condition which, in the opinion of the Investigator, causes the subject to be medically unfit to receive Sacituzumab Govitecan, or unsuitable for any other reason.<br>11. Patients with leptomeningeal carcinomatosis are excluded.

**Assessments of:**<br>• **Efficacy**<br><br><br>• **Safety**<br><br><br><br><br>• **Tumor Penetration**	Progression-free survival<br>Overall survival<br><br>Safety endpoints<br>• Incidence and severity of adverse events will be monitored using CTCAE version 5.0<br>• Changes in lab parameters, vital signs and weight<br><br>Tumor samples will be tested for total SN-38, free SN-38, and SN-38G with concentrations correlated against plasma concentration at the same timepoint.
**Procedures (Summary)**	All 30 subjects will receive study drug Sacituzumab Govitecan preoperatively. Intraoperative tissue collection will follow with contemporaneous CSF (depending on tumor location) and whole blood (serum) sampling. Samples will be tested for total SN-38and free SN-38, as well as SN-38G. Following recovery from surgery, patients will resume treatment<br><br>Adverse events will be reported for all events occurring after the start of treatment until 30 days after study drug is discontinued or subsequent cancer therapy is initiated.

1 INTRODUCTION

1.1 SCIENTIFIC BACKGROUND

1.1.1 Sacituzumab govitecan is an antibody drug conjugate that targets Trop-2 for the selective delivery of SN-38 to tumors. Trop2, also known as trophoblast antigen 2, is a cell surface glycoprotein which is differentially expressed in a number of epithelial tumors[1, 2]. Originally identified in trophoblast cells, it is an intracellular calcium signal transducer which provides crucial signals for cells with requirements for proliferation, survival, self-renewal, and invasion[3]. A number of approaches have been used to target Trop2, including antibody based therapy using the RS7-3G11 (RS7) murine IgG1 antibody[4][5]. In vitro studies have demonstrated antibody-dependent cellular cytotoxicity activity against Trop-2 positive carcinomas[6]. Based upon the broad reactivity of RS7 with epithelial cancers and its ability to internalize, Goldenberg et al hypothesized that conjugating RS7 to the topoisomerase inhibitor SN-38 (the active metabolite of irinotecan) could result in sustained release of the drug within the tumor environment as well as achieve direct intracellular delivery[7]. The antibody drug conjugate (ADC), Sacituzumab govitecan

(SG), showed enhanced efficacy in rodent models with minimal toxicity in primates, leading to clinical trials. Most recently, SG was evaluated in a single-arm, multicenter trial in 69 patients with relapsed/refractory metastatic triple negative breast cancer (TNBC) at a 10 mg/kg starting dose on days 1 and 8 of 21-day cycles[8]. The results were impressive given the refractory nature of this population, with a confirmed objective response rate of 30% (including 2 complete responses), with responses occurring early (median onset of 1.9 months) and being durable (median duration 8.9m). SG has since been granted priority review designation by the FDA, with approval anticipated upon resolution of manufacturing issues. If approved, this will be the first targeted therapy for non-BRCA mutated TNBC.

Data has also been made available showing a similar level of efficacy in refractory hormone receptor positive breast cancer[9]. Fifty-four patients having received at least 2 prior treatments, with a median of 3 prior hormonal agents and 2 prior chemotherapy regimens were treated with SG. The overall response rate was 31% with 17 partial responses, and a clinical benefit rate of 48%.

1.1.2 Brain metastases remain a clinical dilemma in triple negative breast cancer.

Nearly half of all women with advanced triple negative breast cancer will be diagnosed with brain metastases[10]. The outcome for these patients is quite poor, with a median overall survival following the diagnosis of brain metastasis of only 7.3 months [11]. Few treatment options exist, with only carboplatin and capecitabine being active agents in triple negative breast cancer while also showing activity within the CNS. Even with these two therapies, systemic treatment has had no impact on overall survival following the diagnosis of brain metastasis for TNBC, in contrast to other subtypes such as luminal or HER2 subsets of patients[12]. Therefore, with the activity of SG in TNBC, and the high relative frequency of brain metastases in TNBC, a logical question follows as to SG's ability to reach and potentially impact brain metastases in TNBC.

1.1.3 Primary brain tumors are characterized by poor survival which correlates with Trop2 expression. In 2019, an estimated 26,170 new cases of primary malignant brain tumors will be diagnosed and 15,475 patients will die from these tumors[13]. The majority of these are astrocytic, with glioblastoma (GBM, Grade IV astrocytoma) representing 48%. GBM is the most common and most aggressive of the primary malignant brain tumors in adults, and hence the primary target of drug development for intracranial malignancy. Currently, front-line treatment consists of a multi-modality approach that includes maximal surgical resection, adjuvant radiation therapy with concurrent temozolomide, and maintenance temozolomide with tumor treatments fields[14]. Once a patient fails standard front-line therapy, prognosis is very poor. The only currently approved therapeutic for salvage treatment is bevacizumab, which has no proven survival benefit [15]. Survival for patients with GBM is currently only a median of 20.9 months. Interestingly, Trop2 expression correlates with not only grade in gliomas, but with malignant features. While normal brain expression of Trop2 is not observed, 95% of GBM samples examined showed moderate to intense staining by immunohistochemistry[16] and strong correlations were observed for both proliferation rate (r=0.68, p=0.01) and microvessel density (r=0.37, p=0.03). Associations have also

been observed for both grade (r=0.17, p<0.01) and time to death (r=-0.16, p<0.01) in the TCGA dataset[17]. This supports GBM as an additional under-met need potentially targetable with SG, with the caveat that this ADC is able to reach its target.

1.1.4 Antibody drug conjugates may achieve higher concentrations within brain tumors than systemic administration of the drug alone. Consensus generated over the years has been that brain tumors, whether primary or secondary, are inaccessible to antibodies due to their large size and the restriction of the blood brain barrier (BBB). However, converging evidence has recently challenged this view. In breast cancer, the ADC Trastusumab-emtansine (T-DM1) was approved based upon a survival benefit in the EMILIA study[18]. The first suggestion of CNS activity was the finding of prolonged OS of 26.8 months in the 10% subset of patients with brain metastases treated with T-DM1 relative to patients treated with lapatinib/capecitabine with 12.9 months [19]. While prospective studies of intracranial response are otherwise lacking, these findings have been corroborated in retrospective analyses where the intracranial response rate has been seen at approximately 25% [20-22]. In GBM, encouraging data has been reported with the EGFR targeting ADC ABT-414, with an objective response rate of 14% and a 6-month progression free survival (PFS) rate of 25%. This was in a recurrent setting where

responses are uncommon. Scott et al had previously shown accumulation of radiolabeled ^{111}In-ch806 (the parental chimeric precursor to ABT-414) within tumor of a patient with anaplastic astrocytoma at day 7 following infusion[23], with no accumulation in normal organs. Animal studies also lend support, with one study using labelled 81C6 demonstrating tumor-to-normal-brain ratios of 25:1 to as high as 200:1[24]. However, there is a clear lack of prospective studies characterizing intracranial intratumoral concentrations for ADCs.

1.1.5 Sacituzumab govitecan is unique as an ADC, with payload and linker characteristics preferable for CNS delivery. SG utilizes a linker designated CL2A (Figure 1). The linkage between CL2A and SN-38 is sensitive to both acidic and alkaline conditions, allowing the detachment of SN38 at a rate of about 50% per the ex vivo serum study [25]. This less stable linker allows for SN-38 to be released at the tumor site after the ADC targets the cells, making the drug accessible to surrounding tumor cells and not just cells directly targeted by the ADC. The payload, SN-38, is the active metabolite of irinotecan which crosses the blood-brain barrier, and is frequently a drug partner in CNS regimens[26]. However, SN-38 has 1000 times more activity than irinotecan itself

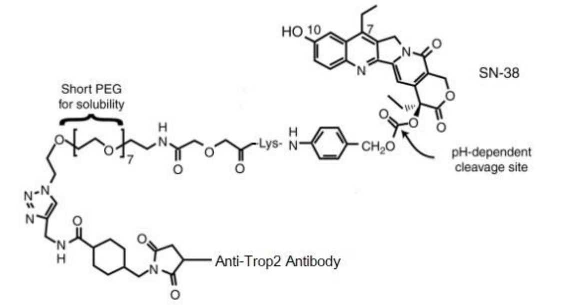

Figure 1. Sacituzumab govitecan structure with the CL2A linker providing a pH dependent cleavage site.

with typical IC50s in the single digit nanomolar range for most GBM cell lines[27]. The combination is one of a hydrolysable linker and release is not solely dependent on pH, with a payload that has high potency and good CNS penetration. While many ADCs may be dependent on disrupted vasculature to reach tumor antigen across the blood brain barrier and internalization for payload release, SG may additionally be able to release SN38 within the vasculature upon encountering the reduced pH of the tumor microenvironment. Free SN-38, being able to cross the blood brain barrier, is thereby hypothetically capable of accumulating in the tumor.

1.1.6 Nonclinical Studies with Sacituzumab Govitecan

1.1.6.1 Human Tumor Xenograft Studies

Sharkey et al [28] examined the delivery of SN-38 to Trop-2 expressing tumors and assessed constitutive products in serum, liver and small intestine in nude mice bearing human tumor xenografts, given a single injection of Irinotecan (40mg/kg; 0.8 mg/mouse, containing 460 µg SN-38 equivalents) or Sacituzumab Govitecan (SG); 1 mg containing

16 μg SN-38 equivalents).

Study design utilized 3 sets of studies for this analysis; nude mice bearing xenografts of the human pancreatic cancer cell line, Capan-1 and NCI-N87. Mice were administered intravenously with single doses containing fixed amount of SG (1.0mg), whereas Irinotecan was given at a dose of 40mg/kg, using the average prestudy weight of the animals to determine a fixed dose for that group. By mass, 1.0mg of irinotecan contains approximately 0.58mg of SN-38. Animals in the first and third study were examined at 5 different intervals and at 2 to 3 intervals in the second study. At select times, tissues were extracted and concentrations of the products measured by reversed-phase highperformance liquid chromatography (HPLC)[28]. HPLC analysis of SN-38, SN-38G and irinotecan required extractions of the harvested tissues.

This study demonstrated the following results favoring Sacituzumab govitecan (IMMU132). In serum, >98% of irinotecan was cleared within 5 minutes ; peak levels of SN-38 and SN-38G (glucuronidated SN-38) were detected in equal amounts and no longer detected after 6 to 8 hours. Intact IMMU-132 cleared with a half-life of 14 hours which closely reflected the in vitro rate of SN-38 released from the conjugate in mouse serum[28]. Area under the curve (AUC) analysis indicated that IMMU-132 delivers 20fold to as much as 136-fold more SN-38 to tumors with irinotecan, with tumor: blood ratios favoring IMMU-132 by 20- to 40-fold. Intestinal concentrations of SN-38/SN-38G also were 9-fold lower with IMMU-132, indicating reduced intestinal uptake that elucidates the lower rate and severity of diarrhea in patients[28].

SN-38 is a highly potent drug but delivery by irinotecan is compromised by (i) rapid clearance from the blood, (ii) poor conversion rate, and (iii) rapid conversion to the inactive SN-38G form. Thus, development of new type of ADC that utilizes SN-38, improves the pharmacokinetics of SN-38 and also provided a mechanism for selective tumor retention via the retention antibody. IMMU-132 delivers much higher levels of SN38 to tumors than irinotecan and importantly, all of the SN-38 delivered to the tumor by IMMU-132 is released in its most potent form along with significantly much lower amounts of SN-38/SN-38G in the intestine reduces the risk of severe diarrhea in patients , which is confirmed in clinical studies[28].

1.1.7 Clinical Study with Sacituzumab govitecan

Study reported by Starodub et al 2015[29] is a phase 1 dose escalation study investigating Sacituzumab govitecan in subjects with metastatic solid cancers. The trial was designed as a 3 + 3 phase 1 design, starting at a dose of 8mg/kg per injection, with dosages given weekly for 2 weeks (day1 and 8) in a 3-week treatment cycle, with cycles repeated until dose-limiting toxicity or progression. Although no preselection based on Trop-2 expression was required, Trop-2 expression in present in >75% of the cases. Twenty five patients were

treated at dose levels of 8 (n=7), 10 (n=6), 12 (n=9) and 18 (n=3) mg/kg. Based on the toxicity studies, hematologic toxicity was reported to be the major doselimiting adverse reaction with grade 3 or 4 neutropenia (n=9) at doses of 12 and 18mg/kg. While the maximum tolerated dose (MTD) was declared to be 12mg/kg, 8.0 and 10.0 mg/kg dose levels were selected for further expansion, as patients were more likely to tolerate additional cycles at these levels with minimal supportive care and responses were observed at these levels. Other common toxicities attributed to sacituzumab govitecan included fatigue, nausea, vomiting (grade 1 or 2) and alopecia. This phase 1 experience has indicated that SG is tolerated with moderate and manageable toxicity, all related to the activity of SN-38 with no evidence of damage to normal tissues known to contain Trop-2. Importantly, SG is active in patients with diverse metastatic solid tumors, even after failing prior therapy with topoisomerase-1 inhibitors.

Ocean et al. 2017 reported preliminary results of the Phase I/II multicenter, dose expansion study to determine the pharmacokinetics and safety of multiple cycles of Sacituzumab govitecan (IMMU-132) at doses of 8 or 10 mg/kg in patients with diverse advanced epithelial cancers[30]. Patients with diverse metastatic cancers who received IMMU-132 at 8mg/kg (n=81) and 10mg/kg (n=97) were examined. The median number of prior therapies for all patients was four. Trop-2 was positive in 93% of the available specimens. Hematological toxicity with grade 3 or 4 neutropenia was observed at both doses causing treatment delays (29% in 8mg/kg group and 34% in 10mg/kg dose group)[30]. 50% of all dose reductions in both the groups occurred after first or second dose in the first cycle. During the phase 2 expansion portion of the trial, investigators were permitted to use a granulocyte - colony - stimulating factor cytokine after the first dose to prophylactically control subsequent neutropenia, with 22% in 8mg/kg group and 26% in 10mg/kg group requiring one hematologic cytokine supportive treatment[30]. Patients with UGT1A1*28*28 haplotype were somewhat more likely to develop grade >/=grade 3 neutropenia than those with other haplotypes; however, because 40% of patients did not experience grade>/=3 neutropenia, and approximately 40% of those with other haplotypes experienced grade>/=3 neutropenia, the management of neutropenic event is more appropriate than screening for patients who have UGT1A1 mutant alleles[30]. At the time of analysis, adverse events for all causalities were reported as 99% and 92% in the 8mg/kg and 10mg/kg cohorts. The 10mg/kg cohort had somewhat higher incidence of grade 3 events including neutropenia, febrile neutropenia and diarrhea, but the overall incidences were not appreciable to warrant selecting 8mg/kg as the starting dose. Free SN-38 levels 30 minutes after the first dose and neutropenia were assessed which showed that there is no evidence to suggest that neutropenia was correlated with the levels of free SN-38 initially in the serum. No serum samples exhibited any antibody response to any component of IMMU-132. In addition, the clinical benefit rate was calculated by combining objective response rates and stable disease that lasted for >/=4months for lung cancer and >/=6months for colorectal and TNBCs, was higher in 10mg/kg group than the 8mg/kg cohort. In conclusion, results from this trial showed that Sacituzumab govitecan is safe with excellent pharmacokinetic profile, manageable toxicity profile along with improved

responses and a good therapeutic index at 10mg/kg, making this dose being chosen for future developments particularly in metastatic TNBC for pursuing accelerated approval.

Study reported by Bardia et al. 2017 is a single arm, multicenter study that enrolled patients with many different cancer types (including mTNBC) to evaluate safety and antitumor activity of Sacituzumab in these patients. 69 patients with mTNBC were enrolled, all were heavily pretreated with median of five lines of therapy since diagnosis and were in the dosing cohort of 10mg/kg, administered intravenously on days 1 and 8 of 21-day repeated cycles[31]. The primary end points were safety and objective response rate (ORR); secondary end points were progression-free survival and overall survival. The confirmed ORR was 30% with median response duration of 8.9 months and early median onset of response was 1.9 months; clinical benefit rate was at 46%[31]. Median Progression free survival (PFS) was 9.6(95% CI, 5.0 to 7.3) months and median overall survival was 16.6 (95% CI, 11.1 to 20.6) months, along with manageable side effect profile[31]. This received Breakthrough Therapy designation from the FDA for the treatment of patients with mTNBC.

1.1.8 Standard Treatment for High Grade Glioma

High-grade gliomas are highly aggressive tumors and invariably recur after standard of care first-line therapy. Currently, front-line treatment consists of a multi-modality approach that includes maximal surgical resection, adjuvant radiation therapy with concurrent temozolomide at 75 mg/m^2 followed by 6 months of single-agent temozolomide at up to 200 mg/m^2. Temozolomide (a prodrug) is a rapidly and nonenzymatically converted to the active alkylating metabolite MTIC [(methyl-triazene-1-yl) – imidazole-4-carboxamide]. The cytotoxic effects of MTIC are manifested through alkylation of DNA at the O6, N7 guanine positions.

This multimodal approach has been the standard of care ever since the publication of the landmark EORTC phase III trial published by Roger Stupp et al in 2005[32] that demonstrated an improved primary end-point of median overall survival of this regimen when compared to adjuvant radiation alone. A median survival of 14.6 months was reported in the temozolomide group versus 12.1 months in the radiation alone group[32]. At that time, radiation alone was considered standard of care in most countries. In the US, common practice was to add adjuvant nitrosurea-based chemotherapy regimen with a small survival benefit with significant adverse effects.

However, once a patient fails standard front-line therapy, prognosis is very poor and new therapies are needed. In the era of targeted therapies, anti-angiogenic drugs have come into the limelight. Bevacizumab, a recombinant humanized monoclonal antibody against VEGF, has been studied extensively over the past several years and has demonstrated rather impressive radiographic and clinical response rates when compared to historical data but the responses are not very long-lasting. The largest study to date was a phase II,

multicenter, open-label, noncomparative trial conducted by Friedman et al in 2009[33] which evaluate the efficacy of bevacizumab (10 mg/kg every 2 weeks), alone and in combination with irinotecan (125 mg/m2 in those non receiving enzyme-inducing anti-epileptic agents and 340 mg/m2 in those who were taking these drugs), in patients with recurrent glioblastoma. In the bevacizumab-alone and the bevacizumab-plus-irinotecan groups, estimated 6-month progression-free survival rates were 42.6% and 50.3%, respectively; objective response rates were 28.2% and 37.8%, respectively; and median overall survival times were 9.2 months and 8.7 months, respectively. Nonetheless, these are all improved outcomes when compared to historical data in the recurrent setting. Bevacizumab obtained accelerated FDA-approval in 2009 for patients with recurrent glioblastoma multiforme. The activity of Sacituzumab govitecan has not yet been assessed in GBM. In order to address CNS penetration of sacituzumab govitecan in these settings, we propose a window of opportunity surgical study to assess bioavailability of Sacituzumab govitecan.

1.1.9 Standard Treatment of Breast Brain Metastasis

Breast cancer is the most frequently diagnosed tumor and second leading mortality in female world[34]. It is the second most common solid malignancy to metastasize to brain, estimated to be present at the time of diagnosis of breast cancer in 0.41% of patients, constituting all 7.56% of all metastatic sites [35]. Patients with triple negative or HER2 positive subtypes experience significantly higher incidence of brain metastasis (BM) occurrence. The cumulative incidence of brain metastasis at 1 and 2 years was 17 and 25% respectively[11]. Retrospective series illustrate overall survival following a diagnosis of TNBC-BM is 4 months despite initial treatment with whole brain radiotherapy (WBRT)[36]. In contrast to patients with endocrine sensitive or HER2-positive BC-BM, the addition of systemic therapy following WBRT has yet to yield improvements in survival for patients with TNBC-BM. The development of effective systemic therapies for patients diagnosed with TNBC-BM is an urgent and unmet medical need. Phase II study of Inipraib in combination with Irinotecan/TBCRC 018 evaluated the efficacy and safety of Iniparib which is a small molecule anti-cancer agent that crosses the blood brain barrier, with the topoisomerase I inhibitor irinotecan in patients with TNBC-BM. This novel, phase II study illustrated that irinotecan and iniparib yields an intracranial clinical benefit rate (CBR) of 30% among patients with new and/or progressive TNBC-BM with a tolerable safety profile and no significant detriment to quality of life[37]. While encouraging, further treatment options are clearly needed.

1.1.10 Biomarkers

As mentioned above, SG targets Trop-2 for the selective delivery of SN-38 to tumors. SG carries SN-38, a topoisomerase inhibitor active in the nanomolar range for most cells and freely cross the blood brain barrier. Brain metastases is a significant concern in mTNBC, but whether this agent is able to target the CNS through the blood brain barrier is unknown. We further hypothesize that while total concentration of SN-38 (both antibody conjugated and

free) will correlate with expression of trop2, free SN-38 will correlate more strongly with intratumoral hypoxia. We will assess carbonic anhydrase IX (CAIX), Trop-2, and γH2AX in surgical specimens by immunohistochemistry, semiautomated image analysis and semiquantitative scoring will be performed, and correlated to results from the above aim.

1.2 RATIONALE FOR DOSE SELECTION AND SCHEDULE

Dosing will be at 10 mg/kg on days 1 and 8 of a 21-day cycle, as previously published and as being assessed for accelerated approval in metastatic TNBC.

1.3 STUDY COMPLIANCE

This study will be conducted in compliance with this protocol, the principles of Good Clinical Practices, and applicable regulations.

2. OBJECTIVES OF THE STUDY

2.1 PRIMARY OBJECTIVES

To determine the extent by which Sacituzumab Govitecan is able to penetrate the blood brain barrier and access tumor tissue by testing for free SN-38, SN-38G and total SN-38 concentrations in tumor tissue as well as in CSF (if available) and serum samples.

2.2 SECONDARY OBJECTIVES

1. To determine the progression-free survival after debulking craniotomy for patients treated with Sacituzumab Govitecan in patients with breast brain metastatic tumors and glioblastoma.
2. To determine overall survival after debulking craniotomy for patients treated with Sacituzumab Govitecan in patients with breast brain metastatic tumors and glioblastoma.
3. To assess the safety of Sacituzumab Govitecan in Breast brain metastatic tumors and Glioblastoma.

2.3 EXPLORATORY OBJECTIVES

1. Correlate tumoral total SN-38 and free SN-38 with tumoral hypoxia and tumoral Trop-2 expression.

3. STUDY DESIGN AND METHODS

3.1 DESIGN

1. Single center, prospective study, non-randomized
2. Sacituzumab govitecan is given as single dose at 10mg/kg administered preoperatively on Day-1 and will receive sacituzumab govitecan post-operatively on Day 1 and 8 of 21-day cycle until progression.
3. Sacituzumab govitecan will be administered intravenously over a period of 3 hours at a dose of 10mg/kg for the first infusion Subsequent infusions may be given over 1-2 hours if previous infusions were well tolerated.
4. Surgery will be followed by post-operative treatment with sacituzumab govitecan given intravenously with standard dose of 10 mg/kg on day1 and day 8 of 21-day cycle, until disease progression

Cohort	# of subjects	Pre-op dose	Post- op dose (Day1 and Day8 of 21-day cycle)
B (GBM)	10	10mg/kg	10mg/kg
A (Breast brain tumors)	20	10mg/kg	10mg/kg

5. Approximately 30 patients will be enrolled. Study includes 2 cohorts, 1 cohort with 10 patients with GBM and 1 cohort of 20 patients with breast brain tumors from breast cancer of any subtype. The study will allow for replacement of subjects if their pathology from the craniotomy surgery on study comes back showing necrosis to achieve the planned total sample size.
6. Sacituzumab govitecan will be administered by IV infusion on day 1 and day 8 of each 21-day cycle. The first infusion will be given over 3 hours. Subsequent infusions may be given over 1-2 hours if previous infusions were well tolerated.

3.1.1 Investigational Sites

This study is a single center, Phase 0 study, non-randomized without dose escalation.

3.1.2 Subject Assignment Methods

This is an open-label study and all of the patients enrolled will receive the study drug Sacituzumab Govitecan pre-operatively and post-operatively. Study includes two cohorts of patients, which includes patients with Glioblastoma multiforme and metastatic breast brain tumors. Presurgical dose will consist of single agent Sacituzumab Govitecan at 10mg/kg IV prior to surgery. Once the patient has recovered from surgery adequately in the opinion of both the PI and the neurosurgeon, and if they continue to meet all eligibility criteria, they will be eligible to continue receiving Sacituzumab govitecan in the post-operative setting.

3.1.3 Statistics

Given the absence of previous data on study drug uptake in human tumors and probable heterogeneity, no formal sample size calculation was conducted. Descriptive statistics will be used to summarize and compare drug concentrations in serum, CSF and tumor tissue. Drug levels from resected specimens will be presented as averages and ranges of determinations, with correction for blood volume using the absorbance of hemoglobin. Tumor concentrations will similarly be expressed as a tumor-to-serum ratios. For serum studies, area-under-the-curve (AUC) concentration will be calculated, ending at the time of tumor resection. The numbers of patients screened, screen failures by reason, the number enrolled and completing the study at each stage and the number and proportion progression-free at each stage will be tabulated. The distribution of time to progression and death will be summarized with Kaplan-Meier curves. Adverse events will be tabulated.

For biomarker exploration, two means of analysis will be used. First, a traditional semiquantitative analysis by a trained pathologist utilizing the H-score. The score is obtained by the formula:

> 3 x percentage of strongly staining nuclei + 2 x percentage of moderately staining nuclei + percentage of weakly staining nuclei, giving a range of 0 to 300.

Additionally, semiautomated image analysis and semiquantitative scoring will be performed using Image ProPlus Software. The hypoxic fraction is calculated as the ratio of area of the region of interest identified with morphometric analysis to total area of the representative area analyzed. The analyses between immunohistochemistry and drug concentration correlation will be calculated by linear regression.

All statistical testing will be two sided with a significance level of 5%. SAS Version 9.3 for Windows (SAS Institute, Cary, North Carolina) will be used throughout. Statistical analyses will be performed by Joel E. Michalek, PhD, Department of Epidemiology and Biostatistics University of Texas Health Science Center at San Antonio.

3.2 TREATMENT REGIMEN

3.2.1 Sacituzumab Govitecan

The dose SG chosen for this study is 10mg/kg consistent with published results [8, 29, 30, 38]. Sacituzumab govitecan will be administered by IV infusion over 3 hours for the first infusion Subsequent infusions may be given over 1-2 hours if previous infusions were well tolerated.

Each dose will be prepared in normal saline for injection in infusion bags and administered intravenously via an infusion pump. The full recommended infusion volume should be used. Each 100 mg of antibody contains approximately 1.6 mg of SN-38, with a mean drug:antibody ratio (DAR) of 7.6:1[29]. Before the start of each infusion, prophylaxis with acetaminophen 650mg po, diphenhydramine 25mg IV, and dexamethasone 10mg is given. Presurgical treatment will be allocated to a single dose either 16 hours preoperatively (t1/2, n=5 per cohort) or 48 hours preoperatively (3x t1/2, n=5 per cohort). These time points were selected based upon the established half-life[30] and results from subcutaneous xenograft studies[29]. Growth factor support (G-CSF, filgrastim, peg-filgrastim) is mandated for the preoperative dose, and at the investigator's discretion for subsequent doses. Following recovery from surgery, therapy consists of two consecutive doses given on days 1 and 8 of a 3-week treatment until unacceptable toxicity or progression. Timing between consent and preoperative dose, as well as resuming dosing postoperatively are as noted in section 5.3.

This study will use a standard dose of 10mg/kg IV in both the cohorts (no dose escalations).

Cohort	# of subjects	Pre and post -operative Sacituzumab dose
A	20	10mg/kg
B	10	10mg/kg

Intravenous Administration of sacituzumab govitecan

Do not administer as an IV push or bolus. Sacituzumab Govitecan is administered intravenously as a slow infusion as described below.

Intravenous access must be well established prior to initiating infusion. At the time of dosing, the IV line will be connected to an infusion container containing the prepared volume of Sacituzumab Govitecan. Either gravity or an infusion pump may be used. Only normal saline should be used as the infusion base solution, since the Sponsor has not examined the compatibility of Sacituzumab Govitecan with other infusion diluents.

The initial infusion should proceed slowly. If vital signs remain stable and in the absence of infusion reactions, the infusion rate can be incrementally advanced following suggested guidelines given in the table below, and following completion, the intravenous line should be flushed slowly with 20 mL normal saline and the end of infusion time recorded. In the event of infusion reactions or vital sign changes, the infusion rate may be slowed, interrupted or terminated, as considered appropriate by the managing physician.

Study drug must be stored under refrigerated conditions (2-8°C) in a locked room that can be accessed only by the pharmacist, the study Investigator, or another duly authorized study/site personnel. The study medications must not be used outside of the context of this protocol. Under no circumstances should the Investigator or other site personnel supply study drug to other Investigators, subjects, or clinics, or allow supplies to be used other than as directed by this protocol without prior written authorization from Immunomedics, Inc.

Drug Interactions

No formal drug-drug interaction studies with Sacituzumab Govitecan have been conducted. SN-38 (the active metabolite of Sacituzumab Govitecan) is metabolized via human UDP-glucuronosyltransferase 1A1(UGT1A1). Concomitant administration of strong inhibitors or inducers of UGT1A1, with Sacituzumab Govitecan, should be avoided due to the potential to either increase (inhibitors) or decrease (inducers) the exposure to SN-38.

3.3 DOSE MODIFICATIONS AND TREATMENT OF SACITUZUMAB GOVITECAN – ASSOCIATED TOXICITIES

Instructions for the infusion of sacituzumab govitecan are provided in Section 6.2. The following sections provide guidance for sacituzumab govitecan administration and management of treatment-related toxicities, including modification of dosing and treatment discontinuation. Toxicities should be managed in accordance with standard institutional practices and accepted treatment guidelines.

3.3.1 Preventative Medications

Infusion-Related Reactions: Pre-medication for prevention of infusion-related reactions with antipyretics and H1 and H2 blockers should be administered before each sacituzumab govitecan infusion. Corticosteroids (hydrocortisone 50 mg or equivalent P.O. or I.V.) may be administered prior to subsequent infusions if the patient had experienced an infusionrelated reaction with a previous infusion. Additional details of recommended treatment of infusion-related reactions are described in Section 3.3.2.1.

Nausea, Vomiting: Sacituzumab govitecan is considered to be moderately emetogenic. Premedication with a 2-drug antiemetic regimen is recommended. If nausea and vomiting are persistent, a 3-drug regimen may be used, including a 5-HT3 inhibitor (ondansetron or palonosetron, or other agents according to local practices), an NK1-receptor antagonist (fosaprepitant), and dexamethasone (10 mg PO or IV). The use of fosaprepitant is preferred over aprepitant as aprepitant is a cytochrome P450 3A4 inhibitor and, therefore, may increase exposure to sacituzumab govitecan. Anticipatory nausea can be treated with

olanzapine. The recommended treatment of delayed nausea and vomiting is described in Section 6.5.2.2.

3.3.2 Management of Sacituzumab Govitecan Toxicities

NCI-CTCAE v5.0 is used to grade the severity of all AEs. The guidelines for management of toxicities associated with sacituzumab govitecan are based on the assessment of severity according to these criteria. Toxicities should be managed in accordance with standard medical practice and treatment guidelines. Instructions for sacituzumab govitecan dose reduction for treatment-related toxicities are provided in Section 3.3.2.2.

3.3.2.1 Infusion-Related Reactions

Infusion-related reactions are defined as symptoms that occur during and within the first 6 hours after the infusion of sacituzumab govitecan. Symptoms can include: fever, chills, rigors, arthralgias, myalgias, urticaria, pruritus, rash, diaphoresis, hypotension, dizziness, syncope, hypertension, dyspnea, cough, and wheezing, as well as severe hypersensitivity reactions including anaphylactic reactions. Infusion-related reactions should be treated in accordance with best clinical practices and standard institutional guidelines. Because of the potential for life-threatening infusion-related reactions, sacituzumab govitecan should only be administered in a setting in which appropriately trained medical staff, emergency equipment and medications are available in the event that resuscitation is required. NCI CTCAE v5.0 is used to grade the severity of all infusion-related adverse events. Premedication for the prevention of infusion-related reactions is described in Section 3.3.1.

Grade 3 and Grade 4 Events

Grade 3 and Grade 4 infusion-related reactions can include severe or clinically significant cardiopulmonary events and severe allergic reactions such as symptomatic bronchospasm and anaphylactic reactions. Grade 3 infusion-related reactions are defined as those which are prolonged and do not improve with symptomatic treatment and/or brief interruption of treatment, reactions that recur following treatment, and reactions that require hospitalization. Grade 4 reactions include potentially life-threatening reactions, requiring urgent intervention. Severe allergic and anaphylactic reactions should be treated in accordance with best clinical practices and standard institutional guidelines. If Grade 3 or Grade 4 infusion-related reactions occur, sacituzumab govitecan should be permanently discontinued.

Grade 2 Events

Grade 2 infusion-related reactions are defined as those that require infusion interruption and respond to symptomatic treatment; prophylactic medications are indicated for ≤24 hours. For Grade 2 infusion-related reactions, the infusion should be interrupted for at least 15 minutes until symptoms resolve. After symptoms resolve, the infusion should be resumed at a slower infusion rate. Recommended infusion rates are provided in Section *<insert cross-reference to section that describes drug administration>*. For recurrent Grade 2

infusion reactions that fails to recover within 6 hours, despite optimal management, permanently discontinue sacituzumab govitecan.

3.3.2.2 Gastrointestinal Toxicities

Nausea, vomiting, and diarrhea are frequent sacituzumab govitecan-associated toxicities. Appropriate treatment, including, as needed, fluid and electrolyte replacement, is required to minimize the risk of serious consequences such as dehydration. Instructions for sacituzumab govitecan dose reduction for treatment-related gastrointestinal toxicities are provided in Section 3.3.2.4.

Nausea and Vomiting

Instructions for the use of premedications for prophylactic treatment of nausea and vomiting and anticipatory nausea are provided in Section 6.5.1. Do not hold the dose of sacituzumab govitecan for Grade 3 nausea unless Grade 3 nausea persists despite maximal optimal medical management. Patients should be treated for delayed nausea and vomiting on Days 2 and 3 with 5-HT_3 receptor antagonist (ondansetron or palonosetron) monotherapy and other agents if needed. Steroids may be added if symptoms do not resolve with these other agents. Consider olanzapine for persistent or anticipatory nausea; an olanzapine dose of 2.5 mg or 5 mg at bedtime is recommended. If a NK1 receptor antagonist is administered, the use of fosaprepitant is preferred over aprepitant as aprepitant is a cytochrome P450 3A4 inhibitor and, therefore, may increase exposure to sacituzumab govitecan.

Diarrhea

Dietary modification should be recommended for the management of diarrhea, including a bland diet, small frequent meals, adequate fluid intake of clear liquids to maintain hydration, and discontinuation of lactose-containing foods and drinks and alcohol. Loperamide should be administered at the onset of treatment-related Grade 1 or Grade 2 diarrhea, at an initial dose of 4 mg, followed by 2 mg with every episode of diarrhea to a maximum dose of 16 mg/day. If diarrhea is not resolved after 24 hours,-add diphenoxylate/atropine.

Add octreotide 100-150 mcg SC tid if diarrhea persists. For Grade 3 or Grade 4 diarrhea, the patient should be hospitalized, and treated with intravenous fluids, and octreotide. Antibiotics can be administered as clinically indicated.

Subjects who exhibit an excessive cholinergic response to treatment with sacituzumab govitecan (e.g., abdominal cramping, diarrhea, salivation, etc.) can receive appropriate premedication (e.g., atropine) for subsequent treatments.

3.3.2.3 Neutropenia

Complete blood cell counts must be obtained prior to each sacituzumab govitecan infusion and should be administered if absolute neutrophil counts meet the following criteria:

- Day 1: ANC > 1500/mm^3
- Day 8: ANC >1000/mm^3

The routine prophylactic use of growth factors is not recommended; however, they may be used in patients who have experienced febrile neutropenia or Grade 3 or Grade 4 neutropenia following previous infusions. Growth factors may also be administered in the setting of neutropenia in patients at high risk of poor clinical outcomes, including those with prolonged neutropenia, ANC <100/mm^3, febrile neutropenia, and serious infections.

3.3.2.4 Sacituzumab Govitecan Dose Delays, Dose Reductions and Discontinuation Guidelines

Dose Delays

Sacituzumab govitecan is to be administered in 21-day cycles on day 1 and day 8; the next cycle should start a minimum 14 days after the day 8 dose. Visit windows of one day prior to and two days after the scheduled infusion are permitted. The scheduled day 1 and day 8 infusions may be delayed for up to one week for treatment-related toxicities. Instructions for dose delays and dose reductions for specific toxicities are summarized below. Dosing may be delayed for >Grade 2 toxicities for a maximum of one week. If the toxicity has improved to ≤Grade 2, the dose should be administered at that time. For a toxicity that delays day 8 dosing, if the toxicity has not resolved to ≤Grade 2 within one week, dosing should resume with the next scheduled cycle, i.e. the next dose will be Day 1 of the following cycle. Regardless of whether the day 8 dose is delayed for toxicity, there should be a minimum of 14 days between the day 8 infusion and the day 1 infusion of the next cycle. There cannot be more than a 5-week dose delay from the last dose or failure to resolve a toxicity within 3 weeks from the last dose.

Dose Reductions and Discontinuation

The major toxicities of sacituzumab govitecan are expected to be gastrointestinal symptoms and hematologic suppression. All patients will be closely monitored over the course of their treatment and aggressively medically managed, including dose reduction and interruption, in order to prevent the need for treatment discontinuation and serious complications of these toxicities. Sacituzumab dose reductions and interruptions will be managed based on toxicity severity, as assessed by NCI CTCAE v5.0. The sacituzumab govitecan dose must not be re-escalated following a dose reduction. Table 1~~Table 1~~ summarizes recommendations for sacituzumab govitecan dose reductions and discontinuations for treatment-related toxicities. Dose reductions will be allowable per physician discretion as long as a patient is still deriving clinical benefit as evidenced by radiographic stability on RANO or RECIST (depending on the cohort).

Table 1: Recommended Dose Reduction Schedule for Sacituzumab Govitecan

**Event** NCI CTCAE v5.0	**Occurrence**	**Recommended dose reduction or action**
**Severe Neutropenia**		
Grade 4 neutropenia ≥7 days, OR<br>Grade 3 febrile neutropenia (absolute neutrophil count <1000/mm^3 and fever ≥38.5°C),<br>OR<br>At time of scheduled treatment, ≥Grade 3 neutropenia which has delayed dosing by 1 week	First	Administer granulocytecolony stimulating factor (G-CSF) or sooner, if clinically indicated
	Second	25% dose reduction
	Third	50% dose reduction
	Fourth	Discontinue treatment
≥Grade 3 neutropenia which delays dosing beyond 3 weeks	First	Discontinue treatment
**Severe Non-Neutropenic Toxicity**		
	First	25% dose reduction
	Second	50% dose reduction
Grade 4 non-hematologic toxicity of any duration,<br>OR<br>Any ≥Grade 3 nausea, vomiting or diarrhea due to treatment that is not controlled with antiemetics and anti-diarrheal agents,<br>OR<br>Other ≥Grade 3 non-hematologic toxicity persisting >48 hours despite optimal medical management,<br>OR<br>At time of scheduled treatment, ≥Grade 3 non-neutropenic hematologic or nonhematologic toxicity, which has delayed dosing by 1 week	Third	Discontinue treatment
≥Grade 3 non-neutropenic hematologic or non-hematologic	First	Discontinue treatment

toxicity, which has delayed dosing for more than 3 weeks		

4. PATIENT POPULATION

4.1 INCLUSION CRITERIA

1. At least 18 years of age
2. Histologically or cytologically documented breast cancer of all subtypes including hormone receptor positive, HER2 positive, and triple negative (Cohort A) with known or suspected parenchymal brain metastases.
3. Recurrent glioblastoma (Cohort B) with documented progression by RANO criteria following standard combined modality treatment with radiation and temozolomide.
4. Plans to undergo craniotomy as part of standard of care. Patients emergently needing surgical debulking due to symptoms of their disease are not eligible.
5. Recovered from toxicities of prior therapy to grade 0 or 1
6. ECOG performance status ≤ 2.
7. Life expectancy of at least 3 months.
8. Acceptable liver function:
   a) Bilirubin ≤ 1.5 times upper limit of normal
   b) AST (SGOT) and ALT (SGPT) ≤ 3.0 times upper limit of normal (ULN);
9. Acceptable renal function: calculated creatinine clearance ≥30mL/minute according to the Cockcroft and Gault formula
10. Acceptable hematologic status (without hematologic support)
    a) ANC ≥1500 cells/uL
    b) Platelet count ≥100,000/uL
    c) Hemoglobin ≥9.0 g/dL
12. All women of childbearing potential must have a negative serum pregnancy test and male and female subjects must agree to use effective means of contraception (surgical sterilization or the use or barrier contraception with either a condom or diaphragm in conjunction with spermicidal gel or an IUD) with their partner from entry into the study through 6 months after the last dose

4.2 EXCLUSION CRITERIA

1. The subject is receiving warfarin (or other coumarin derivatives) and is unable to switch to low molecular weight heparin (LMWH) before the first dose of study drug.

2. The subject has evidence of acute intracranial or intratumoral hemorrhage either by MRI or computerized tomography (CT) scan. Subjects with resolving hemorrhage changes, punctate hemorrhage, or hemosiderin are eligible.
3. The subject is unable to undergo MRI scan (eg, has pacemaker).
4. The subject has received enzyme-inducing anti-epileptic agents within 14 days of study drug (eg, carbamazepine, phenytoin, phenobarbital, primidone).
5. Patients whose only lesion undergoing resection has received stereotactic radiation within the past 3 months
6. The subject has received any of the following prior anticancer therapy:
   a) Biologic agents (antibodies, immune modulators, vaccines, cytokines) within 21 days prior to first dose of study drug
   b) Prior treatment with Sacituzumab Govitecan
7. Patients receiving UGT1A1 (Uridine diphosphate glucuronosyl transferase 1A1) inhibitors or inducers.
8. History of significant cardiovascular disease, defined as:
   i) Congestive heart failure greater than New York Heart Association (NYHA) Class II according to the NYHA Functional Classification.
   ii) Unstable angina or myocardial infarction within 6 months before enrollment.
   iii) Serious cardiac arrhythmia.
9. Clinically significant ECG abnormality, including:
   i) Marked Baseline prolonged QT/QTc interval (ie, a repeated demonstration of a QTc interval >500 ms) demonstrated on ECG at Screening.
   ii) History of risk factors for torsade de pointes (eg, heart failure, hypokalemia, family history of long QT Syndrome).
10. Any medical or other condition which, in the opinion of the Investigator, causes the subject to be medically unfit to receive Sacituzumab Govitecan, or unsuitable for any other reason.
11. Patients with leptomeningeal carcinomatosis are excluded.

5. PROCEDURES

Subjects are expected to participate for up to treatment duration with continued follow up for survival until one year after the first dose of study drug. Please refer to Appendix A, Schedule of Assessments, for an overview of the study assessments. Subjects who withdraw from the study before all follow-up procedures have been performed will be managed and documented as described in Section 8, Removing Subjects from the Study.

A summary of visits and clinical procedures is found in Appendix A, Schedule of Assessments. The total duration of the active part of the study for each subject will be approximately 18 weeks, divided as follows:

- Up to 3 weeks predose (screening period)
- Up to 6 weeks for presurgical dosing, surgery and recovery.

- 3-week treatment periods of Sacituzumab govitecan
- Study termination visit 1-2 weeks after last dose of study medication

When a subject has completed the study termination or early termination visit, he/she and/or a family member will be contacted for survival information every 3 months until one year from completion.

All subjects will be screened within 21 days prior to Presurgical Day -1. Vital signs, clinical laboratory test results, weight and AEs will be used to assess safety. Efficacy will be assessed based on tumor assessments (objective response rate, progression-free survival and duration of response) conducted at intervals during the study. Subjects who have not progressed after 6 cycles may be permitted to continue therapy on a case-by-case basis.

During screening, candidates for the study will be fully informed about the nature of the study and possible risks, and will receive a copy of the informed consent for review. Candidates must read the consent form and sign the document after the investigator has answered all questions to the candidate's satisfaction. Further procedures can begin only after the consent form has been signed. The original signed consent form will be retained by the investigator and a copy will be given to the candidate. Candidates will be evaluated for entry into the study according to the stated inclusion and exclusion criteria (Section 4, Study Population). The investigator will evaluate the results of all examinations, including clinical laboratory tests, and will determine each candidate's suitability for the study. The investigator must know the baseline results before enrollment. The pregnancy test for females of reproductive potential must be negative for those subjects to proceed to enrollment. All screening procedures must be done within 21 days of presurgical day -1, unless otherwise specified. The following procedures will be performed to establish each candidate's general health and qualifications for possible enrollment into the study:

- Obtain signed, written informed consent and permission to use protected health information, (in accordance with the Health Insurance Portability and Accountability Act or HIPAA). Refusal to sign informed consent and permission excludes an individual from the study.
- Record medical history, including cancer history: histology of primary tumor (including degree of differentiation), date of cancer diagnosis, types and dates of prior anti-tumor therapy (including surgery, radiation therapy, systemic therapy),and date of most recent disease progression. For patients with GBM, isocitrate dehydrogenase mutation status and methyl guanine methyl transferase (MGMT) methylations status (if available) will be recorded as well.
- Record recent medication history, including vitamins, herbal preparations, blood products, and other over the counter (OTC) drugs.
- Record blood pressure (BP), heart rate (HR), respiratory rate (RR) and temperature measurements. In subjects with known significant pulmonary disease, measure oxygen saturation using pulse oximeter after a 2 minute walk
- Perform a complete physical examination, including height and weight.

- Perform tumor assessment with MRI of the brain per RANO criteria both preoperatively and postoperatively within 5 days of dosing respectively (refer to Appendix C). MRI only, no tumor measurement needed at screening.
- Assess Eastern Cooperative Oncology Group (ECOG) Performance Status score (see Appendix B, Eastern Cooperative Oncology Group Performance Status Scale).
- Draw blood samples for hematology, chemistry and coagulation.
- Obtain a blood sample for serum HCG pregnancy test in female subjects of child-bearing potential (all female subjects unless surgically sterilized or at least 1 year post-menopausal).
- Obtain a urine sample for urinalysis with micro.
- Obtain a baseline ECG to assess for cardiac arrhythmias or evidence of recent cardiac events.
- Review inclusion and exclusion criteria (see Section 4, Study Population).

Prior to proceeding to post-surgical treatment, all breast cancer patients should be engaged in a discussion of alternate systemic treatment options. This is particularly important in patients with extracranial disease with a limited prior treatment history or newly diagnosed disease. Specifically, in Her2 positive patients, trastuzumab, pertuzumab, and TDM1 are established treatment options that should be considered prior to experimental therapy. For hormone receptor positive patients, endocrine therapy and a CDK4/6 inhibitor should be considered prior to experimental therapy. Patients completing the surgical component, who have not already received these agents, should be encouraged to do so following surgery. Documentation of this discussion should be placed in the source documents for each patient. This is not applicable to recurrent glioma patients.

5.3 TREATMENT PERIOD- CYCLES 1-6

Study drug should be administered with 60 min of the pre-surgical timepoint and within ±2 days of the nominal time point for postoperative cycles. Lab tests used for determining dosing must be done within 5 days before the first dose of study drug (Cycle l/Day 1), within 3 days before Days 1 and 8 of all subsequent cycles. All other required study assessments should be obtained within 5 days of the nominal time point unless otherwise specified. Subjects must receive their single pre-surgical dose of study drug within 21 days of the start of screening. Postoperative dosing (Cycle 1/Day 1) may begin no sooner than 21 days postoperatively, and a maximum of 60 days postoperatively. Patients not able to begin treatment postoperatively within 60 days of surgery should be removed from the study, unless exception is given by the study PI and a representative from Immunomedics.

5.3.1 Presurgical Evaluations and Procedures

The following procedures will be done in all subjects:

- If screening assessments have not been performed within 3 days of planned surgery, then screening assessments will be repeated except for informed consent, demographics, MRI, medical/surgical history, and ECG;
- Serum and whole blood will be collected from patients on day -1 within 30 minutes at the end of Sacituzumab infusion, and intra-operatively at the time of tissue collection for biomarker analysis.

5.3.2 Procedures (Day 1 of each cycle)

Before administering Sacituzumab govitecan, the following procedures will be done in all subjects, at the post-surgical visit or within 5 days of Cycle 1 Day 1:

- Record interim medical history since screening;
- Confirm that subject continues to meet inclusion/exclusion criteria;
- Record concomitant medications for previous 14 days;
- Draw serum and whole blood samples for biomarker analysis

The following procedures will be done predose on all subjects within a 5-day window prior to Cycle 1 Day 1 and within 3 days of Day 1 for **All Future Cycles** (unless otherwise specified) and before administering Sacituzumab govitecan in subjects:

- Assess Eastern Cooperative Oncology Group (ECOG) Performance Status score;
- Assess whether subject is adequately hydrated for administration of study drugs
- Cycle 2 and all subsequent cycles: Record AEs since last visit;
- Record concomitant medications since last cycle;
- Record weight and vital signs;
- Detailed physical exam including neurologic assessment.
- Draw blood samples for hematology and chemistry;
- Draw serum and whole blood samples for biomarker analysis
- Obtain (serum or urine) pregnancy test (prior to start of Cycle 1, 3 and 5 only; females of childbearing potential)
- Administer Sacituzumab

5.3.3 Procedures (Day 8 of each cycle)

- A physical exam from Day 1 will be taken into consideration for D8 of cycle as well.
- Record AEs since the last visit, on the day of infusion D8
- Measure and record vital signs (BP, HR, RR, temperature) on D8
- Obtain blood samples for hematology, chemistry on Day8
- Administer Sacituzumab govitecan on D8 of 21-day treatment cycle.

5.4 STUDY TERMINATION/EARLY STUDY TERMINATION AND SURVIVAL FOLLOW-UP

5.4.1 Study Termination / Early Study Termination

This visit will occur at least 2 weeks after the last dose of Sacituzumab govitecan treatment for subjects who terminate early. The following will be done at the Termination/Early Termination visit:

- Record concomitant medications, including vitamins, herbal preparations, blood products, and other OTC drugs since the last visit; • Record AEs since the last visit;
- Perform a complete physical examination, including weight;
- Assess Eastern Cooperative Oncology Group (ECOG) Performance Status score;
- Measure and record vital signs (BP, HR, RR, temperature);
- Obtain blood samples for hematology and chemistry
- Obtain serum and whole blood samples for biomarker analysis
- Obtain a blood sample for serum HCG pregnancy test in female subjects of child-bearing potential (all female subjects unless surgically sterilized or at least 1 year post-menopausal);
- Perform tumor assessments, using the same imaging assessments done at baseline, if not done within past 4 weeks.

In accordance with good medical practice, any ongoing study drug-related AE present at study termination, including a clinically significant laboratory test abnormality, will be followed until resolved or until the event stabilizes and the overall clinical outcome has been ascertained. Adverse events starting up to 30 days after the last dose of study medication, or until the start of an alternate medication, may be collected by telephone contacts.

5.4.2 Survival Follow-up

When a subject has completed the study termination or early termination visit, he/she and/or a family member will be contacted for survival information every 3 months until one year from last dose. Anti-tumor therapy (description and dates) since the last contact will be collected at each survival follow up.

5.4.3 Termination Reasons

- Death.
- Withdrawal of consent for the study at any time for any reason.
- Lost to follow-up.
- Sponsor decision.

5.5.1 Safety Procedures

5.5.1.1 Physical Examination

A complete physical examination will be performed at screening and at study termination or early study termination and results will be recorded by the investigator (or designee). Limited physical examination will be done within 5 days before Day 1 of each cycle

(q21days). Body weight will be measured on Day 1 of every cycle. The results of the physical examinations will be used for safety monitoring purposes only. At each study visit, according to good medical practice, the subject's general health (e.g., appearance, adequacy of hydration, presence of illness or injury, temperature, and vital signs indicative of a concurrent illness) will be assessed to determine whether continued dosing is appropriate.

5.5.1.2 Vital Signs

BP, HR, RR and temperature will be measured at the following time points:

- Screening
- All subjects: Day 1 and Day 8 of every cycle (predose and postdose for each study drug administered)
- Study Termination or Early Study Termination

Blood pressure and HR measurements should be obtained with the subject's arm unconstrained by clothing or other material. The measurements will be obtained with the appropriate cuff size from the opposite arm from that used for blood sampling, where possible, which is supported at the level of the heart. All BP measurements will be obtained from the same arm throughout the dosing period. The cuff should be placed on the designated arm at least 10 minutes prior to taking BP measurements.

5.5.1.3 Disease Assessment

Patients in Cohort A will be assessed for bicompartmental progression simultaneously. CNS and non-CNS (extracranial) disease will be scored according to RANO-BM and RECIST 1.1 criteria, respectively. Any unscheduled disease assessments (e.g. due to clinical worsening) should include evaluations of both CNS and non-CNS disease. Patients in cohort B will be assessed by RANO. Patients in both cohorts will be assessed at screening, within 3 days prior to the first post-surgical cycle, and following every third cycle (with a 5-day window of the subsequent cycle). MRI only, no tumor measurements needed at screening.

6. MATERIALS AND SUPPLIES

6.1 DOSAGE, FORM, DRUG SUPPLY AND STORAGE

Sacituzumab govitecan is formulated for investigational use only. Formulation consists of 10 mg/mL sacituzumab govitecan formulated in 25 mM MES, pH 6.5, together with the following excipients (25 mM trehalose, 0.01% Polysorbate 80), which are then lyophilized. Glass vials containing 200 mg of sacituzumab govitecan as a sterile, non-pyrogenic, lyophilized powder are to be stored under refrigerated conditions (2-8°C) until used.

It contains 200 mg sacituzumab govitecan with a minimum extractable drug quantity and label claim of 180 mg sacituzumab govitecan.

While the content of the vial has not changed and remains 200 mg, this label change to 180 mg represents the minimum extractable quantity of drug from a vial of sacituzumab govitecan (IMMU-132).

Each vial is labeled “For Clinical Trial Use Only. Caution: New Drug-Limited by Federal (or United States) Law to Investigational Use. Sponsor: Immunomedics, Inc” and identified by study drug name, lot number, and dose. Since the formulated drug product contains no preservative, vials should be used only once. Sacituzumab govitecan will be provided in single vial packaging.

6.2 STUDY DRUG PREPARATION AND ADMINISTRATION

Reconstitute with normal saline and dilute immediately into infusion bags. Initiate the infusion within 1 hour of reconstitution/dilution. If infusion is delayed beyond 1 hour, refrigerate at 2-8°C for no more than 4 hours from reconstitution/dilution prior to infusion. If refrigerated, allow the diluted solution to come to room temperature prior to administration. If infusion does not begin within 4 hours after reconstitution/dilution, dispose of the original preparation and prepare a new infusion bag by reconstitution and dilution from new vials. Discard any unused portion in the vial. The product does not contain a preservative.

Appropriate use of aseptic technique should be employed in preparing the dose. Allow the sacituzumab govitecan vials to warm to room temperature to allow faster dissolution. The mg of lyophilized powder in each vial should be reconstituted using 20 mL of 0.9% sterile sodium chloride (normal saline). The reconstituted solution should be gently shaken and allowed to dissolve for up to 15 minutes. Calculate the prescribed dose in mg based on the patient’s bodyweight at the beginning of EACH cycle (or more frequently for > 10% change in body weight or if required by institutional policy; see Table 6.1 below).

The appropriate calculated amount should then be withdrawn from the supplied vials of study drug. The Sponsor recommends using a 21-gauge needle. Inject the solution into a glass or plastic infusion container slowly to minimize foaming and do not shake the contents. Adjust the volume in the infusion container as needed with normal saline to obtain a concentration of 1.1-3.4 mg/mL (total volume should not exceed 500 mL). Only normal sterile saline should be used since the stability of the reconstituted product has not been determined with other infusion- based solutions. The prepared study drug is stable for up to 8 hours at room temperature, however, as there is no preservative, the prepared study drug should be refrigerated.

Table 6.1 Sacituzumab Govitecan Sample Dose Preparation- 58kg subject, reconstituted vial containing 10mg/ml and 250 mL infusion bag

Preparation for 10 mg/kg Dosing			
Step 1	Calculate total dose	58 kg x 10 mg/kg	580 mg dose
Step 2	Reconstitute the required number of vials by adding 20 mL 0.9% Sodium Chloride Injection, USP (normal saline) to each	n/a	Result: Approximately 20 mL of 10 mg/mL reconstituted IMP in each vial
Step 3	Calculate required volume (number of mL's needed) of reconstituted IMP to equal desired dose	580 mg dose ÷ 10 mg/mL	58 mL
Step 4	Select sterile 0.9% Sodium Chloride Injection, USP (normal saline) infusion bag of appropriate volume	250 mL for 580 mg dose (within 1.1 – 3.4 mg/mL ratio)	250 mL infusion bag selected
Step 5	Withdraw volume from infusion bag equivalent to the volume of reconstituted IMP to be added	250 mL normal saline infusion bag – 58 mL extracted	192 mL normal saline remaining in infusion bag
Step 6	Slowly transfer the calculated amount of reconstituted IMP into the infusion bag and gently mix.	58 mL IMP + 192 mL normal saline	250 mL total volume
Step 7	Verify final concentration is within the range of 1.1-3.4 mg/mL	580 mg ÷ 250 mL	2.32 mg/mL

Administer the first infusion over 3 hours. Monitor the subject during the infusion, and for at least 30 minutes after infusion. Subsequent infusions may be administered over 1-2 hours if previous infusions were well tolerated.

6.3 DRUG ACCOUNTABILITY

The investigator is responsible for the control of drugs under investigation. Adequate records of the receipt and disposition of all study drug shipped to the site must be maintained. Records will include dates, quantities received, quantities dispensed, and the identification codes of the subjects who received study drug. The individual administering the study drug will write the study number, subject number, date, and start/stop times of administration on the study drug label, and the Drug Accountability Record, as appropriate.

6.4 DISPOSITION OF USED AND UNUSED VIALS OF STUDY MEDICATION

All used, partially used, and unused vials must be retained by the pharmacist. Periodically throughout and at the conclusion of the study, inventory checks and accountability of study materials will be conducted by a representative of Immunomedics or its designated agent. Once accountability is completed, a Immunomedics representative will authorize the return to a designated facility or release for local destruction of all used, partially used, and

unused vials. Partially used or empty vials may be destroyed prior to Immunomedics's review per the site's standard institutional procedures and local and federal regulations as applicable, and with appropriate documentation after two study staff members have reconciled and documented the count on an Accountability Record. The completed Drug Accountability and Drug Destruction Record(s) will be returned to Immunomedics.

The investigator's copy of the Drug Return Destruction Record(s) must accurately document the destruction of all study drug supplies. Records will also include dates, lot numbers, and quantities returned to Immunomedics or its designated agent or destroyed locally.

7 MANAGEMENT OF INTERCURRENT EVENTS

Comprehensive assessments of any apparent toxicity experienced by the subject will be performed throughout the course of the study. Study site personnel will report any clinical AE, whether observed by the investigator or reported by the subject.

7.1.1 Grading of Toxicity

Clinical AEs or abnormal laboratory test results will be assessed by the principal investigator or other designated other physician, in accordance with the CTCAE v5.0 criteria.

7.1.2 Monitoring and Treatment of Toxicity

A physician or other qualified medical professional (e.g. Physician Assistant, Nurse Practitioner) designated by the Principal Investigator will manage and treat any toxicity.

7.2 ADVERSE EVENTS

A physician or other qualified medical professional (e.g. Physician Assistant, Nurse Practitioner) designated by the Principal Investigator will assess the seriousness, severity, and causality of an AE based on the following definitions.

7.2.1 Defining Adverse Events

An adverse event (AE) is any undesirable event occurring to or in a subject enrolled in a clinical trial, whether or not the event is considered related to the study drug (Sacituzumab Govitecan). This includes the time periods beginning after the first administration of study drug until 30 days after the last dose of study drug or until a start of alternative therapy.

Adverse events include the following types of occurrences:

1) Suspected adverse reactions;
2) Other medical experiences, regardless of their relationship to the study drug, such as injury, causes for surgery, accidents, increased severity of pre-existing symptoms, apparently unrelated illnesses, and significant abnormalities in clinical laboratory values, physiological testing, or physical examination findings; and 3) Reactions from drug overdose, abuse, withdrawal, sensitivity, or toxicity.

7.2.1.1 Serious Adverse Events

A serious adverse event (SAE) is any adverse' experience that occurs at any dose and results in any of the following outcomes.

1) **Death.** This includes any death that occurs during the conduct of the clinical study, including deaths that appear to be completely unrelated to the study drug (e.g., car accident). However, deaths that occur due to disease progression are not considered SAEs, but should be reported as a death on study. If a subject dies during the study, and an autopsy is performed, the autopsy results should be sent to Immunomedics. Possible evidence of organ toxicity and the potential relationship of the toxicity to the study rug are of particular interest. The autopsy report should distinguish between the relationship between the underlying diseases, their side effects, and the cause of death.
2) **Life-threatening adverse experience.** This includes any AE during which the subject is, in the view of the investigator, at immediate risk of death from the event as it occurs. This definition does not include any event that may have caused death if it had occurred in a more severe form.
3) Persistent or significant disability or incapacity
4) Inpatient hospitalization or prolongation of existing hospitalization
5) Congenital anomaly or birth defect
6) Other medically important event which, according to appropriate medical judgment, may require medical or surgical intervention to prevent one of the outcomes listed above.
7) Pregnancy occurring in subjects treated with Sacituzumab Govitecan should be reported using the serious adverse event reporting form.

7.2.1.2 Non-serious adverse events

A non-serious AE includes any AE that is not defined as an SAE.

7.2.1.3 Unexpected adverse events

An unexpected AE is any AE that is not identified in nature, severity or frequency.

7.2.2 Documenting All Adverse Events

Record all AEs as descriptive findings (symptoms, or laboratory, physical exam, or vitals abnormalities) or diagnoses if etiology is known. Included are all AEs that occur after the

start of treatment or within 30 days of administration of the last dose of study drug. Record AEs of any severity and AEs that are assessed as serious or not serious.

Note: Unchanged, chronic conditions and cancer symptoms present at baseline are NOT AEs and should not be recorded unless there is an exacerbation or worsening in severity of a chronic condition or cancer symptom after the first administration of study drug until 30 days after the last dose of study drug. Chronic conditions and/or cancer symptoms that exacerbate or worsen in severity should be documented as a "worsening" condition. Death due to disease progression and measures of disease progression collected as efficacy endpoints (eg increasing tumor size or new lesions) are not considered adverse events, but should be collected as termination reasons (if applicable) and/or noted in tumor assessment appropriate. Other reasons for death occurring during the AE reporting period are SAEs and should be reported as such.

7.2.2.1 Grading of Adverse Events

Severity of AEs or clinically significant laboratory test results will be assessed in accordance with the grading scale presented in the Common Toxicity Criteria for Adverse Events (CTCAE) version 4.0. A copy of this document can be found at the following internet site: http://cte.info.nih.gov/reporting/ctc.html. Clinically significant abnormal laboratory results and lab results requiring an intervention will be recorded as AEs and should describe whether the lab result was increased or decreased. The following definitions for rating severity of AEs will be used for events not covered in the CTCAE.

Grade 1: Mild; awareness of signs or symptoms that are easily tolerated, are of minor irritant type, cause no loss of time from usual activities, do not require medication or further medical evaluation, and/or are transient.

Grade 2: Moderate; signs or symptoms sufficient to interfere with function but not activities of daily living.

Grade 3: Severe; signs or symptoms sufficient to interfere with activities of daily living; signs and symptoms may be of a systemic nature, or require further medical evaluation and/or treatment.

Grade 4: Disabling or with life-threatening consequences. (This definition does not include any event that might have caused death if it had occurred in a more severe form.) Grade 5: Death

7.2.2.2 Relationship to Study Drug

Using the following criteria, investigators will assess whether there is a reasonable possibility that the study drugs (Sacituzumab Govitecan) caused or contributed to the AE.

Yes - The time sequence between the onset of the AE and study drug administration is consistent with the event being related to study drug; and/or there is a possible biologic

mechanism for study drug causing or contributing to the AE; and the AE may or may not be attributed to concurrent/underlying illness, other drugs, or procedures.

No - Another cause of the AE is most likely; and/or the time sequence between the onset of the AE and study drug administration is inconsistent with a causal relationship; and/or a causal relationship is considered biologically unlikely.

7.2.2.3 Abnormal Laboratory Test Results as Adverse Events

The investigator will monitor the laboratory test results and determine the clinical significance of any result that falls outside of the reference range. In accordance with good medical practice, any clinically significant abnormal laboratory test results must be followed until resolved or stabilized. Abnormal laboratory test results should not be reported as AEs unless, in the opinion of the investigator, the results constitute or are associated with a clinically relevant condition or require intervention.

In the event of unexplained, clinically significant abnormal laboratory test results, the tests should be repeated immediately and followed up until the values have returned to within the reference range or to baseline for that subject.

7.2.3 Reporting and Documenting Serious Adverse Events

Serious adverse events (SAE) that occur at any time point after the first dose of study drug until 30 days after the last dose of study drug must be reported. SAEs must be reported as per institutional policy and as required under the Data Safety Monitoring Plan (see 10.6 for DSMP).

1) Submit all known subject information (listed below) within 24 hours of knowledge of the SAE occurrence. The following information should also be entered in the database (or as much as possible to obtain and still report the event within 24 hours):
    a) Subject's Demographic Data
    b) Subject's weight
    c) Description of SAE, including date of onset and duration, severity, and outcome
    d) All dosing data of study drugs administered up to the date the SAE occurred
    e) Action taken regarding study drug administration
    f) Relationship of SAE to study drugs
    g) Concomitant medications, including regimen and indication
    h) Intervention, including concomitant medications used to treat SAE
    i) Pertinent laboratory data and diagnostic tests conducted and date
    j) Pertinent medical history of subject
    k) Date of hospital admission discharge (if applicable)
    l) Date of death (if applicable)

2) Perform appropriate diagnostic tests and therapeutic measures, and submit all followup substantiating data, such as diagnostic test reports and autopsy report to Immunomedics.
3) Conduct appropriate consultation and follow-up evaluations until the events are resolved, stabilized, or otherwise explained by the principal investigator.
4) Review each SAE report and evaluate the relationship of the SAE to study treatment and to the underlying disease. Immunomedics will determine whether the SAE is unexpected in nature.
5) Based on a cooperative assessment of the SAE with Immunomedics, a decision for any further action will be made. The primary consideration is subject safety. If the discovery of a new SAE related to the study drug raises concern over the safety of its continued administration to subjects, Immunomedics will take immediate steps to notify the FDA.
6) The investigator must report all SAEs and unexpected problems promptly to his or her IRB/IEC, as appropriate (see ICH Guidelines, Good Clinical Practice [E6]).

Other actions regarding SAEs might include the following:

a) Protocol amendment
b) Discontinuation or suspension of the protocol
c) Modification of informed consent to include recent findings
d) Informing current study participants of new findings
e) Identification of specific AEs as drug-related

7.2.4 Follow Up of Adverse Events

All AEs are followed until they are resolved or determined to be irreversible or otherwise explained by the principal investigator.

7.3 CONCOMITANT AND EXCLUDED THERAPY

UGT1A1 (Uridine diphosphate glucuronosyl transferase 1A1) inhibitors or inducers are to be avoided during study participation due to potential affect on SG metabolism.

All medications and blood products (prescription and over-the-counter including herbal preparations) taken within 21 days of Cycle l/Week 1 will be recorded by the investigator (or designee). The reason(s) for treatment, dosage, and dates of treatment should be recorded in the source documents. In addition, concomitant medications used to treat adverse events occurring up to 30 days after the last dose of study drug will be recorded.

Female subjects who have been on hormone replacement therapy (HRT) for menopausal symptoms for a period of at least 2 months will not be excluded from the study provided the HRT regimen remains unchanged during the conduct of the study.

Concurrent radiation is allowed. For patients receiving radiation to the brain following craniotomy, a two-week window must elapse prior to resuming study drug. The decision for radiation following surgical resection will be left to the treating team as per standard of care and does not impact the primary endpoint.

8. REMOVING SUBJECTS FROM THE STUDY

8.1 CRITERIA FOR TERMINATION

Subjects are free to discontinue (withdraw) at any time during this clinical trial. If a subject withdraws from participation in the study during the treatment period, he or she should be encouraged to return for an early termination visit for evaluation of safety (see Section 5.4.1, Study Termination/Early Study Termination).

The investigator has the right to discontinue any subject from study drug administration or study participation. Reasons for subject discontinuation may include, but are not limited to, the following:

- Clinically significant deterioration of the subject's condition;
- Disease progression;
- Requirement for other anti-tumor therapy during the study;
- Noncompliance;
- Pregnancy;
- Significant AE;
- Subject's right to withdraw from the study at any time, with or without stated reason;
- Significant protocol violation;
- Lost to follow-up;
- Death;

Any other reason that, in the opinion of the principal investigator, would justify the removal of a subject from the study. The primary consideration in any determination to discontinue a subject's participation must be the health and welfare of the subject.

All subjects will be instructed on the importance of complying with the requirements of the study. It is expected that subjects will complete all of the necessary visits. If a subject does not return for follow-up visits as directed or does not adhere to the study requirements, the investigator will determine if early withdrawal should occur.

8.2 DOCUMENTATION

The primary reason for early removal of a subject from the study must be documented clearly, and must be completed for any subject who has received any amount of drug during the treatment period. If the reason for early withdrawal is an AE or an abnormal laboratory value, the specific event or test result must also be recorded.

8.3 PROCEDURES FOR SUBJECTS WHO WITHDRAW EARLY

Following early termination, the subject should be informed about which evaluations are necessary to monitor his or her safety. In addition, subjects should be encouraged to complete any procedures or evaluations outlined in Section 5.4, Study Termination/Early Study Termination.

8.4 REPLACEMENT OF SUBJECTS

Subjects who have not received Sacituzumab Govitecan will be replaced. Subject enrollment numbers are unique and will not be re-assigned.

9. CONDITIONS FOR INITIATING, MODIFYING, OR TERMINATING THE STUDY 9.1 INSTITUTION REVIEW

The investigator will submit this protocol, any protocol modifications, and the subject consent form to be used in this study to the local Institutional Review Board (IRB) for review and approval. A letter confirming IRB approval of the protocol and subject consent form, and an IRB approved informed consent form must be forwarded to Immunomedics prior to the enrollment of subjects into the study.

9.2 INFORMED CONSENT

The investigator or his or her designee must explain to the subject, in the presence of a witness, the purpose and nature of the study, the study procedures, and the possible adverse effects, and all other elements of consent as defined in 21 CFR Part 50 and Clinical Trial Directive or ICH E6 guidelines before enrolling that subject in the study. It is the investigator's (or designee's) responsibility to obtain informed written consent from each subject, or if appropriate, the subject's parent or legal guardian.

9.3 MODIFICATIONS

If any modifications in the experimental design, dosages, parameters, subject selection, or any other sections of the protocol are indicated or required, the investigator will consult with Immunomedics before such changes are instituted. Modifications will be accomplished through formal amendments and approval from the appropriate IRB.

9.4 DEVIATIONS

The PI will consider any deviations from the protocol on a case-by-case basis. The investigator or other designated alternate in his absence will contact the local DSMB (DSMB2) as soon as possible to discuss the associated circumstances. The principal investigator and the DSMB will then decide whether the subject should continue to participate in the study. All protocol deviations and the reasons for such deviations must be noted in the source documents and will be reported to the CTRC DSMC and to the IRB as per standard institutional policy.

9.5 TERMINATION

Enrollment will be suspended if ≥2 patients are unable to undergo surgical resection as planned due to toxicity/adverse events related to the study drug or procedures. If following review by DSMB these events are confirmed related to study drug or procedures, the study will be terminated. Further, if Immunomedics or the investigators discover conditions during the course of the study that indicate it should be discontinued, an appropriate procedure for terminating the study will be instituted, including notification of the appropriate regulatory agencies and IRB.

10. INVESTIGATOR'S RESPONSIBILITIES

10.1 RESPONSILITIES/PERFORMANCE

The investigator will ensure that this study is conducted in accordance with all regulations governing the protection of human subjects. The investigator will adhere to the basic principles of "Good Clinical Practice," as outlined in Title 21 of the Code of Federal Regulations (CFR), Part 312, Subpart D, "Responsibilities of Sponsors and Investigators";

2 1 CFR, Part 50, "Protection of Human Subjects"; 21 CFR, Part 56, "Institutional Review Boards"; and the US Food and Drug Administration (FDA) guideline entitled "Good Clinical Practice: Consolidated Guideline". For studies conducted outside of the USA, the investigator will ensure adherence to the principles outlined in the International Conference on Harmonization (ICH) E6 "Guideline for Good Clinical Practice". Additionally, this study will be conducted in compliance with the Declaration of Helsinki and with any local laws

and regulations of the country in which the research is conducted. The investigator will ensure that all work and services described in or associated with this protocol will be conducted in accordance with the investigational plan, applicable regulations, and the highest standards of medical and clinical research practice. The investigator is responsible for the control of drugs under investigation. The investigator will provide copies of the study protocol and Investigator's Brochure to all sub- investigators, pharmacists, and other staff responsible for study conduct.

10.2 CONFIDENTIALITY

The investigator must ensure that each subject's anonymity will be maintained and each subject's identity will be protected from unauthorized parties. A number will be assigned to each subject upon study entry and the number and the subject's initials will be used to identify the subject for the duration of the study. Documents submitted to Immunomedics should not identify a subject by name. Documents that are not submitted to Immunomedics (eg, signed consent form) will be maintained by the investigator in strict confidence.

10.3 INSTITUTIONAL REVIEW

The investigator will submit this protocol, any protocol modifications, and any accompanying material provided to the subject (eg, informed consent form, subject information sheets, or descriptions of the study used to obtain informed consent) to the appropriate IRB for review and approval. A letter confirming IRB approval of the protocol and subject consent forms, and an IRB approved informed consent form must be forwarded to Immunomedics prior to the enrollment of subjects into the study. A copy of the approved subject consent form will also be forwarded to Immunomedics.

10.3.1 Modifications

If any modifications in the experimental design, dosages, parameters, or subject selection are indicated or required, the investigator will consult with Immunomedics (or vice versa) before such changes are instituted. Modifications will be accomplished through formal amendments to this protocol and approval by the appropriate IRB. Copies of all subsequent IRB approvals (e.g., protocol amendments) must be sent to Immunomedics.

10.3.2 Protocol Deviations

The PI will consider any deviations from the protocol on a case-by-case basis. The investigator or other designated alternate in his absence will contact the local DSMB (DSMB2) as soon as possible to discuss the associated circumstances. The principal investigator and the DSMB will then decide whether the subject should continue to participate in the study. All protocol deviations and the reasons for such deviations must be

noted in the source documents and will be reported to the Mays Cancer Center at UT Health San Antonio and the IRB as per standard institutional policy.

10.3.3 Termination

If Immunomedics and/or the investigator(s) discover conditions during the course of the study that indicate it should be discontinued, an appropriate procedure for terminating the study will be instituted, including notification of the appropriate regulatory agencies and the IRB.

10.4 INFORMED CONSENT AND PERMISSION TO USE PROTECTED HEALTH INFORMATION

It is the responsibility of the investigator to obtain written informed consent from each subject participating in this study after adequate explanation, in lay language, of the methods, objectives, anticipated benefits, and potential hazards of the study. The investigator must also explain that the subject is completely free to refuse to enter the study or to discontinue participation at any time (for any reason) and receive alternative conventional therapy as indicated. Prior to study participation, each subject will sign an IRB approved informed consent form, which will be in form and substance acceptable to Immunomedics, and receive a copy of same (and information leaflet, if appropriate). For subjects not qualified or able to give legal consent, consent must be obtained from a parent, legal guardian, or custodian.

The investigator or designee must explain to the subject before enrollment into the study that for evaluation of study results, the subject's protected health information obtained during the study may be shared with Immunomedics, regulatory agencies, and IECs/IRBs. It is the investigator's (or designee's) responsibility to obtain permission to use protected health information per HIPAA from each subject, or if appropriate, the subjects' parent or legal guardian.

10.5 SOURCE DOCUMENTATION AND INVESTIGATOR FILES

The investigator must maintain adequate and accurate records to fully document the conduct of the study and to ensure that study data can be subsequently verified. These documents should be classified into 2 separate categories: (1) investigator study file and (2) subject clinical source documents that corroborate data collected.

Subject clinical source documents would include hospital clinic patient records; physician's and nurse's notes; appointment book; original laboratory, ECG, EEG, radiology, pathology, and special assessment reports; pharmacy dispensing records; subject diaries;

signed informed consent forms; consultant letters; and subject screening and enrollment logs.

The following will be documented in source documents at the site:

1) Medical history/physical condition and diagnosis of the subject before involvement in the study sufficient to verify protocol entry criteria (if not already present);
2) Study number, assigned subject number, and verification that written informed consent was obtained (each recorded in dated and signed notes on the day of entry into the study);
3) Progress notes for each subject visit (each dated and signed);
4) Study drug dispensing and return;
5) Review of laboratory test results;
6) Adverse events (action taken and resolution);
7) Concomitant medications (including start and stop dates); and
8) Condition of subject upon completion of or early termination from the study.

10.5.1 Exclusion Log

The investigator must keep a record listing all patients considered for entry into the study but subsequently excluded. The reason for each exclusion will be recorded in the Subject Exclusion Log.

10.6 DATA SAFETY MONTIORING PLAN

A Data and Safety Monitoring Plan is required for all an individual protocols conducted at Mays Cancer Center at UT Health San Antonio. All protocols conducted at Mays Cancer Center are covered under the auspices of the Mays Cancer Center Institutional Data Safety Monitoring Plan (DSMP).

The Mays Cancer Center Institutional DSMP global policies provide individual trials with:

- institutional policies and procedures for institutional data safety and monitoring,
- an institutional guide to follow,
- monitoring of protocol accrual by the Mays Cancer Center Protocol Review Committee,
- review of study forms and orders by the Forms Committee,
- independent monitoring and source data verification by the Mays Cancer Center QA Monitor/Auditor
- tools for monitoring safety events,
- monitoring of UPIRSO's by the Director of Quality Assurance and DSMC,
- determining level of risk (Priority of Audit Level Score – PALS) ,
- oversight by the Data Safety Monitoring Committee (DSMC), and
- verification of protocol adherence via annual audit for all Investigator Initiated Studies by the Mays Cancer Center Quality Assurance Division.

10.6.1 Monitoring Safety

Due to the risks associated with participation in this protocol, the Mays Cancer Center DSMB2 in conjunction with the Principal Investigator will perform assessment of adverse events, adverse event trends and treatment effects on this study. The Mays Cancer Center DSMB2 acts as an independent Data Safety Monitoring Board (DSMB) for IIS conducted at Mays Cancer Center. The Mays Cancer Center will monitor data throughout the duration of a study to determine if continuation of the study is appropriate scientifically and ethically. An additional layer of review is provided by the Mays Cancer Center Data Safety Monitoring Committee (DSMC) who will review the DSMB's quarterly reports.

Baseline events and adverse events will be captured using the Mays Cancer Center Master Adverse Events Document for each patient using CTCAE V4.0 for the grading and attribution of adverse events. Usage of the CTRC Master Adverse Events Document centrally documents:

- the event and grades the seriousness of the event,
- if the event was a change from baseline,
- the determination of the relationship between the event and study intervention, • if the event was part of the normal disease process, and
- what actions were taken as a result of the event.

10.6.2 Reporting Requirements

For this study, the Master Adverse Events Documents collected on patients for this protocol will be reviewed by the Principal Investigator on a monthly basis to determine if a serious safety problem has emerged that result in a change or early termination of a protocol such as: • dose modification,

- suspending enrollment due to safety or efficacy, or
- termination of the study due to a significant change in risks or benefits.

The PI will provide the DSMB2 with the monthly findings for discussion and review during their meetings. Specific areas of concern that will be reported to the DSMB2 regarding this study that would qualify as an endpoint are:

- evidence from surgically resected tissue that Sacituzumab Govitecan does in fact NOT penetrate the blood brain barrier and as such would have no therapeutic benefit to patients on study
- unacceptable toxicity that would prevent patients from receiving treatment and therefore benefit

As per the Mays Cancer Center DSMP, any protocol modifications, problematic safety reports, unanticipated problems, and suspension or early termination of a trial must be reported to all members of the research team. Suspension and early termination of a trial

must also be reported immediately to the Director of Quality Assurance who will promptly notify the sponsor and the UTHSA IRB.

The PI will review the Master Adverse Events documents to determine the significance of the reported events and will provide findings using the Investigator Initiated Study Quarterly DSMC Report Form on a monthly basis with the DSMB2. The DSMB2 will review the information provided by the PI and report to the Mays Cancer Center DSMC on a quarterly basis unless an emergent issue has been identified. The Investigator Initiated Study Quarterly DSMC Report Form includes information on adverse events, current dose levels, number of patients enrolled, significant toxicities per the protocol, patient status (morbidity and mortality) dose adjustments with observed response, and any interim findings. Any trend consisting of three or more of the same event will be reported to the Mays Cancer Center DSMC for independent review outside of the quarterly reporting cycle, which begins three months following protocol start up. The DSMB2 will also provide its findings to the Mays Cancer Center's Regulatory Affairs Division so that it may be provided to the UTHSA IRB with the protocol's annual progress report. Conflict of interest is avoided by the independent reviews of the Mays Cancer Center DSMB2, Mays Cancer Center DSMC and by ongoing independent review of UPIRSO's by the Director of Quality Assurance.

All SAE and UPRISO's will be reported following MCC and UTHSCSA institutional guidelines.

UTHSCSA SAE/UPRISO REPORTING REQUIREMENTS		
Type Event	Report to	Timeframe
All AE, SAE and UPIRSO	Regulatory Affairs and DQA	Same as other notification timeframes except for
		SAE/AE which should be reported on Monday for the prior week
SAE	Clinical Trial Sponsor	within 24 hours
AE/SAE	UTHSA IRB	Annually
UPIRSO - all	Clinical Trial Sponsor	within 24 hours of the PI determining a UPIRSO exists
UPIRSO - life threatening	UTHSA IRB	within 48 hours of the PI determining a UPIRSO exists
UPIRSO - non-life threatening	UTHSA IRB	within 7 days of the PI determining a UPIRSO exists

Expedited reporting may not be appropriate for specific expected adverse events for certain later Phase II and Phase III protocols. In those situations the adverse events that will not have expedited reporting must be specified in the text of the approved protocol. An expected Grade 3 event that is definitely related to the investigational agent is only to be

reported if the patient is hospitalized using the generic reporting criteria. For instance, in a trial of an investigational agent where Grade 3 diarrhea requiring hospitalization is expected, only diarrhea requiring ICU care (Grade 4) might be designated for expedited reporting.

Serious adverse events on NCI sponsored trials utilizing a commercially available agent (with no IND's involved) will additionally be reported via the FDA's Medwatch program.

10.6.2 Assuring Compliance with Protocol and Data Accuracy

As with all studies conducted at Mays Cancer Center at UT Health San Antonio, the PI has ultimate responsibility for ensuring protocol compliance and data accuracy/integrity. Protocol compliance, data accuracy and reporting of events is further ensured by an annual audit conducted by the Data Safety Officer, whose audit report is shared with the PI, the research team and will be reviewed by the MCC DSMC.

Mays Cancer Center DSMB Membership

The Mays Cancer Center has two DSMB's with a primary set of members specific to the histology of the study consisting of UTHSA faculty and staff. This Protocol will utilize DSMB#1 for Hematological studies or DSMB#2 for Solid Tumor Studies.

As per NCI guidelines and to eliminate conflict of interest (financial, intellectual, professional, or regulatory in nature), the Mays Cancer Center DSMB specific to this study will not treat patients on this protocol. Usage of the DSMB specific to the histology has been created to ensure that experts in that histology are represented on the DSMB assembled for this protocol, but may be expanded, at the PI's discretion, to include other members which may include:

- Experts in the fields of medicine and science that are applicable to the study (if not currently represented on the DSMB),
- Statistical experts,
- Lay representatives,
- Multidisciplinary representation, from relevant specialties including experts such as bioethicists, biostatisticians and basic scientists, and
- Others who can offer an unbiased assessment of the study progress.

Additional or alternate membership of in the DSMB is selected by the DSMC chair, in conjunction with the PI of this protocol.

Mays Cancer Center DSMB Charter and Responsibilities

The Mays Cancer Center DSMB will provide information on the membership composition, including qualifications and experience to both the UTHSA IRB and Mays Cancer Center

PRC for review. The Mays Cancer Center DSMB for this study will act as an independent advisory board to the PI and will report its findings and recommendations to the PI, the UTHSA F and the Mays Cancer Center DSMC. Mays Cancer Center DSMB reports will utilize the Investigator Initiated Study Quarterly DSMC Report Form and meetings will occur on a monthly basis to review any updates from the prior meeting.

Once the protocol is activated, if not already established elsewhere in the protocol the Mays Cancer Center DSMB will establish and provide:

- procedures for maintaining confidentiality;
- statistical procedures including monitoring guidelines, which will be used to monitor the identified primary, secondary, and safety outcome variables;
- consider factors external to the study when relevant information becomes available, such as scientific or therapeutic developments that may have an impact on the safety of the participants or the ethics of the study;
- plans for changing frequency of interim analysis as well as procedures for recommending protocol changes;
- recommendation of dose escalation, MTD recommendation of early termination based on efficacy results;
- recommendation of termination due to unfavorable benefit-to-risk or inability to answer study questions;
- recommendation of continuation of ongoing studies;
- recommend modification of sample sizes based on ongoing assessment of event rates; and
- review of final results and publications.

11. REFERENCES

1. Shvartsur, A. and B. Bonavida, *Trop2 and its overexpression in cancers: regulation and clinical/therapeutic implications.* Genes & cancer, 2015. **6**(3-4): p. 84-105.
2. Trerotola, M., et al., *Upregulation of Trop-2 quantitatively stimulates human cancer growth.* Oncogene, 2012. **32**: p. 222.
3. Cubas, R., et al., *Trop2 expression contributes to tumor pathogenesis by activating the ERK MAPK pathway.* Mol Cancer, 2010. **9**: p. 253.
4. Shih, L.B., et al., *In vitro and in vivo reactivity of an internalizing antibody, RS7, with human breast cancer.* Cancer Res, 1995. **55**(23 Suppl): p. 5857s-5863s.
5. Stein, R., et al., *Advantage of yttrium-90-labeled over iodine-131-labeled monoclonal antibodies in the treatment of a human lung carcinoma xenograft.* Cancer, 1997. **80**(12 Suppl): p. 2636-41.
6. Varughese, J., et al., *High-grade, chemotherapy-resistant primary ovarian carcinoma cell lines overexpress human trophoblast cell-surface marker (Trop-2) and are highly sensitive to immunotherapy with hRS7, a humanized monoclonal anti-Trop-2 antibody.* Gynecol Oncol, 2011. **122**(1): p. 171-7.

7. Cardillo, T.M., et al., *Humanized Anti-Trop-2 IgG-SN-38 Conjugate for Effective Treatment of Diverse Epithelial Cancers: Preclinical Studies in Human Cancer Xenograft Models and Monkeys.* Clinical Cancer Research, 2011. **17**(10): p. 3157-3169.
8. Bardia, A., et al., *Efficacy and Safety of Anti-Trop-2 Antibody Drug Conjugate Sacituzumab Govitecan (IMMU-132) in Heavily Pretreated Patients With Metastatic Triple-Negative Breast Cancer.* Journal of Clinical Oncology, 2017. **35**(19): p. 2141-2148.
9. Bardia, A., et al., *Efficacy of sacituzumab govitecan (anti-Trop-2-SN-38 antibody-drug conjugate) for treatment-refractory hormone-receptor positive (HR+)/HER2- metastatic breast cancer (mBC).* Journal of Clinical Oncology, 2018. **36**(15_suppl): p. 1004-1004.
10. Lin, N.U., et al., *Sites of distant recurrence and clinical outcomes in patients with metastatic triple-negative breast cancer: high incidence of central nervous system metastases.* Cancer, 2008. **113**(10): p. 2638-45.
11. Jin, J., et al., *Incidence, pattern and prognosis of brain metastases in patients with metastatic triple negative breast cancer.* BMC Cancer, 2018. **18**(1): p. 446.
12. Niwinska, A., M. Murawska, and K. Pogoda, *Breast cancer subtypes and response to systemic treatment after whole-brain radiotherapy in patients with brain metastases.* Cancer, 2010. **116**(18): p. 4238-47.
13. Kruchko, C., et al., *CBTRUS Statistical Report: Primary Brain and Other Central Nervous System Tumors Diagnosed in the United States in 2011–2015.* Neuro-Oncology, 2018. **20**(suppl_4): p. iv1-iv86.
14. Stupp, R., et al., *Maintenance Therapy With Tumor-Treating Fields Plus Temozolomide vs Temozolomide Alone for Glioblastoma: A Randomized Clinical Trial.* JAMA, 2015. **314**(23): p. 2535-43.
15. Friedman, H.S., et al., *Bevacizumab alone and in combination with irinotecan in recurrent glioblastoma.* J Clin Oncol, 2009. **27**(28): p. 4733-40.
16. Ning, S., et al., *TROP2 expression and its correlation with tumor proliferation and angiogenesis in human gliomas.* Neurol Sci, 2013. **34**(10): p. 1745-50.
17. Harvard, B.I.o.M.a., *Broad Institute TCGA Genome Data Analysis Center (2016): Correlation between mRNA expression and clinical features.*
18. Verma, S., et al., *Trastuzumab emtansine for HER2-positive advanced breast cancer.* N Engl J Med, 2012. **367**(19): p. 1783-91.
19. Krop, I.E., et al., *Trastuzumab emtansine (T-DM1) versus lapatinib plus capecitabine in patients with HER2-positive metastatic breast cancer and central nervous system metastases: a retrospective, exploratory analysis in EMILIA.* Ann Oncol, 2015. **26**(1): p. 113-9.
20. Fabi, A., et al., *T-DM1 and brain metastases: Clinical outcome in HER2-positive metastatic breast cancer.* Breast, 2018. **41**: p. 137-143.
21. Jacot, W., et al., *Efficacy and safety of trastuzumab emtansine (T-DM1) in patients with HER2-positive breast cancer with brain metastases.* Breast Cancer Res Treat, 2016. **157**(2): p. 307-318.
22. Bartsch, R., et al., *Activity of T-DM1 in Her2-positive breast cancer brain metastases.* Clin Exp Metastasis, 2015. **32**(7): p. 729-37.
23. Scott, A.M., et al., *A phase I clinical trial with monoclonal antibody ch806 targeting transitional state and mutant epidermal growth factor receptors.* Proc Natl Acad Sci U S A, 2007. **104**(10): p. 4071-6.

24. Zalutsky, M.R., et al., *Pharmacokinetics and Tumor Localization of ¹³¹ILabeled Anti-Tenascin Monoclonal Antibody 81C6 in Patients with Gliomas and Other Intracranial Malignancies.* Cancer Research, 1989. **49**(10): p. 2807-2813.
25. Cardillo, T.M., et al., *Sacituzumab Govitecan (IMMU-132), an Anti-Trop-2/SN-38 Antibody–Drug Conjugate: Characterization and Efficacy in Pancreatic, Gastric, and Other Cancers.* Bioconjugate Chemistry, 2015. **26**(5): p. 919-931.
26. Vredenburgh, J.J., et al., *Experience with irinotecan for the treatment of malignant glioma.* Neuro-oncology, 2009. **11**(1): p. 80-91.
27. *Genomics of Drug Sensitivity in Cancer Project* Available from: https://www.cancerrxgene.org/translation/Drug/1494.
28. Sharkey, R.M., et al., *Enhanced Delivery of SN-38 to Human Tumor Xenografts with an Anti-Trop-2–SN-38 Antibody Conjugate (Sacituzumab Govitecan).* 2015. **21**(22): p. 51315138.
29. Starodub, A.N., et al., *First-in-Human Trial of a Novel Anti-Trop-2 Antibody-SN-38 Conjugate, Sacituzumab Govitecan, for the Treatment of Diverse Metastatic Solid Tumors.* Clin Cancer Res, 2015. **21**(17): p. 3870-8.
30. Ocean, A.J., et al., *Sacituzumab govitecan (IMMU-132), an anti-Trop-2-SN-38 antibodydrug conjugate for the treatment of diverse epithelial cancers: Safety and pharmacokinetics.* Cancer, 2017. **123**(19): p. 3843-3854.
31. Bardia, A., et al., *Sacituzumab Govitecan-hziy in Refractory Metastatic Triple-Negative Breast Cancer.* N Engl J Med, 2019. **380**(8): p. 741-751.
32. Stupp, R., et al., *Radiotherapy plus concomitant and adjuvant temozolomide for glioblastoma.* N Engl J Med, 2005. **352**(10): p. 987-96.
33. Goli, K.J., et al., *Phase II trial of bevacizumab and irinotecan in the treatment of malignant gliomas.* 2007. **25**(18_suppl): p. 2003-2003.
34. Siegel, R.L., K.D. Miller, and A. Jemal, *Cancer Statistics, 2017.* CA Cancer J Clin, 2017. **67**(1): p. 7-30.
35. Martin, A.M., et al., *Brain Metastases in Newly Diagnosed Breast Cancer: A PopulationBased Study.* JAMA Oncol, 2017. **3**(8): p. 1069-1077.
36. Niwinska, A., M. Murawska, and K. Pogoda, *Breast cancer brain metastases: differences in survival depending on biological subtype, RPA RTOG prognostic class and systemic treatment after whole-brain radiotherapy (WBRT).* Ann Oncol, 2010. **21**(5): p. 942-8.
37. Anders, C., et al., *TBCRC 018: phase II study of iniparib in combination with irinotecan to treat progressive triple negative breast cancer brain metastases.* Breast Cancer Res Treat, 2014. **146**(3): p. 557-66.
38. Heist, R.S., et al., *Therapy of Advanced Non-Small-Cell Lung Cancer With an SN-38-AntiTrop-2 Drug Conjugate, Sacituzumab Govitecan.* J Clin Oncol, 2017. **35**(24): p. 27902797.

Appendix A: Schedule of Assessments

Study Procedures	Screening (w/n 21 days of Surg)	Pre-Surg (Surg D-1)	Surgery (Surg D1)	PostSurg Visit	PostSurgical C1 (PSC1)		PostSurgical C2 (PSC2)		PostSurgical C3 (PSC3)		PostSurgical C4 (PSC4)		PostSurgical C5 (PCS5)		PostSurgical C6 (PSC6) and future cycles		EOT *
Day					Day 1	Day 8	Day 1	Day 8	Day 1	Day 8	Day 1	Day 8	Day 1	Day 8	Day 1	Day 8	
Informed Consent	X																
Demographics	X																
Med/Surg History	X																
Physical Exam (to include Adverse events and con meds)[C,F]	X	X		X	X	X	X		X		X		X		X		X
Vitals Signs	X	X		X	X	X	X	X	X	X	X	X	X	X	X	X	X
Height	X	X															
Weight	X	X		X	X	X	X	X	X	X	X	X	X	X	X	X	X
ECOG	X	X		X	X	X	X		X		X		X		X		X
ECG	X																
Hematology labs[A]	X	X			X	X	X	X	X	X	X	X	X	X	X	X	X
Chemistry labs[A]	X	X			X	X	X	X	X	X	X	X	X	X	X	X	X
Coagulation labs[A, B]	X	X			X												
Urinalysis[A, D]	X				X												
Pregnancy test	X				X				X				X				X
Sacituzumab 10mg/kg IV		X			X	X	X	X	X	X	X	X	X	X	X	X	
Neulasta (onPro)[H]		X				X		X		X		X		X		X	
Tumor Assessment[E]	X			X							X						X
Serum Sampling		X	X		X		X		X		X		X		X		X
Tumor Sampling			X														

CSF Sampling / LP [G]			X			X											

A – Blood draws must be within 5 days of C1D1, and within 3 days of D1 and within 1 day of D8.

B – INR only if patient is on warfarin

C – Physical exam can be done within 5 days before Day 1 of Cycles 1, 3, and 5.

D – Screen: UA with micro. Other visits, urine dipstick for protein & glucose. If positive or change from BL, complete urinalysis w/micro.

E – Tumor Assessments at screening, post-surgical visit, and after every 9 weeks. Scanning to be done within 5 days of dosing at specified time points. MRI only, no tumor measurements needed at screening.

F – Physical exams due on Day 1 and D8 for presurgical and first postsurgery cycles then only on D1 unless clinically indicated.

* - Occurs 3-4 weeks after the start of C6 or at least 2 weeks after the last dose of Sacituzumab for subjects who terminate early. Survival follow-up will be done by telephone after EOT every 3 months until one year from last dose.

G – Lumbar puncture is an optional procedure and will only be done with subject consent if CSF was not collected during surgery.

H- Neulasta (OnPro) is only required at the pre-surgical dose on D-1. Subsequent cycles are optional and dependent on ANC results.

APPENDIX B: EASTERN COOPERATIVE ONCOLOGY GROUP (ECOG) PERFORMANCE SCORE SCALE

Grade	Description
0	Fully active, able to carry on all pre-disease performance without restriction
1	Restricted in physically strenuous activity but ambulatory and able to carry out work of a light or sedentary nature, e.g., light house work, office work
2	Ambulatory and capable of all self-care but unable to carry out any work activities. Up and about more than 50% of waking hours
3	Capable of only limited self-care, confined to bed or chair more than 50% of waking hours
4	Completely disabled. Cannot carry on any self-care. Totally confined to bed or chair
5	Dead

APPENDIX C: RANO TUMOR RESPONSE

Table: Summary of the RANO Response Criteria

	CR	PR	SD	PD#
**T1-Gd +**	None	≥50% decrease	<50% decrease-<25% increase	≥25% increase*
**T2/FLAIR**	Stable or decrease	Stable or decrease	Stable or decrease	Increase*
**New Lesion**	None	None	None	Present*
**Corticosteroids**	None	Stable or decrease	Stable or decrease	**NA**
**Clinical Status**	Stable or increase	Stable or increase	Stable or increase	Decrease*
**Requirement for Response**	All	All	All	Any*

CR=complete response; PR=partial response; SD=stable disease; PD=progressive disease

Progression occurs when any of the criteria with * is present

NA: Increase in corticosteroids alone will not be taken into account in determining progression in the absence of persistent clinical deterioration

APPENDIX D:

Immunomedics "Product Inspection Form"

**Site ID:**		**Facility Name**		**Date Shipment Received and Inspected:**	
**Street Address:**		**City:**		**ZIP:**	
**Lot Number:**		**Expiration:**		**# of Vials Inspected:**	
**Temperature Upon Receipt:**					

Any Items that are marked "YES" requires a comment to be denoted

	**ITEM**	**STATUS (Circle)**	**COMMENTS**
1	Is particulate matter present (e.g. foreign mater, hair, glass, r, metal) fibe **Note**: The container should be free of particulates	**YES / NO**	
2	Is there any discoloration of the reconstituted drug product? **Note**: The drug product should be clear and pale yellow	**YES / NO**	
3	Is the vial broken (cracked neck, cracked body)?	**YES / NO**	
4	Is there any unsatisfactory sealing of the cap including any cation of indi tampering or evidence that could indicate abnormal of material, conditions container or cap under normal lighting conditions?	**YES / NO**	
5	Is the stopper damaged (stained, cracked, dented)?	**YES / NO**	
6	Is there visible cloudiness in the reconstituted drug product tion? solu	**YES / NO**	
7	Is the vial empty?	**YES / NO**	

Visual Inspection Performed By: ________________________________ **Date:** _______________

NOTE: For items denoted "YES", the subject Vial/Lot should be quarantined, and notify your CRA to contact the Immunomedics Quality Department should be contacted within 1 business day of the finding.

Immunomedics, Inc. Visual Inspection Form for IMMU-132 version date/number: 12-Feb-2019 ver 3.0

APPENDIX E: SUMMARY OF CHANGES

Protocol changes from Version 5 to Version 6

- Editorial changes made throughout
- Update to Synopsis, Planned Total Sample Size and Section 3: Study Design and Methods, Design to allow for replacement of subjects if their pathology from the craniotomy surgery on study comes back showing necrosis to achieve the planned total sample size. This change was made to avoid additional waiver requests from the IRB due to this specific scenario.

Protocol changes from Version 4 to Version 5

- Updated the version number and date of protocol
- Update the Responsible Study Coordinator or Research Nurse for the study.
- Updated the Responsible Pharmacist for the study

Protocol changes from Version 3 to Version 4

- Updated the version number and date of protocol
- Update the Responsible Study Coordinator or Research Nurse for the study.
- Increased the total number of patients from 20 to 30 as funding for an additional 10 breast to brain mets patients was granted.
- Adjusted the cohort names to be consistent throughout the protocol.
- Added a statement under Dose Reductions and Discontinuation to state that dose reductions will be allowable per physician discretion as long as a patient is still deriving clinical benefit as evidenced by radiographic stability on RANO or RECIST (depending on the cohort). This statement has been added after a waiver was requested and approved in the event this happens again.
- Section 5.4.1 Study Termination / Early Study Termination was updated. Patients will be able to continue on treatment after 6 cycles without PI approval as long as patient is still receiving benefits and hasn't progressed. The study termination visit will not occur 3-4 visits after cycle 6, it will occur at least 2 weeks after the last dose of Sacituzumab govitecan.
- Section 5.4.2 Survival Follow-up was changed from every 3 months until one year from first dose to every 3 months until one year from last dose.
- Appendix A: Schedule of Assessments was updated. Footnote for Neulasta was added stating that Nuelasta is only required at the pre-surgical dose on D-1. Subsequent cycles are optional and dependent on ANC results. Updated footnote E to state that tumor assessments will be done every 9 weeks instead of every third cycle. Updated for consistency in scans in case subjects are delayed. Updated footnote F – physical exams are to be done on Day 1 and Day 8 (not Day 18) for presurgical and first postsurgery cycles then only on D1 unless clinically indicated.
- Added Appendix D to include the summary of changes for the protocol.

SUPPLEMENTARY NOTE 3

A Phase 0, Investigator Initiated Study to Determine the Bioavailability of Sacituzumab Govitecan in Breast Brain Metastasis and Glioblastoma

Study Center:
Mays Cancer Center at UT Health San Antonio
Institute for Drug Development
7979 Wurzbach Rd., 4th Floor Zeller Building
San Antonio, TX 78229

Principal Investigator:
Andrew Brenner, MD, PhD
Mays Cancer Center at UT Health San Antonio
Institute for Drug Development
7979 Wurzbach Road, 4th floor
Mail Code# 8232
San Antonio, TX 78229
Tel. (210) 450-5936
Fax. (210) 692-7502
E-mail: brennera@uthscsa.edu

Co-Principal Investigator:
John R. Floyd, MD
UTHSA
Department of Neurosurgery
7703 Floyd Curl Drive
Mail Code# 7843
San Antonio, TX 78229
Tel. (210) 567-5625
Fax. (210) 567-6066
E-mail: floydj@uthscsa.edu

Sub-Investigators:
Prathibha Surapaneni
PGY-5, Fellow
Department of Hematology Oncology
UT Health San Antonio

See FDA 1572

IND# **TBD**

CTMS# 19-0069

Protocol Version 2, dated May 7 2019

Responsible Study Coordinator or Research Nurse:

Emily Cleveland, RN
(210) 450- 5958
ClevelandE@uthscsa.edu

Pharmacy:

IDS Pharmacy
Ivan Reveles, PharmD
(210) 450- 757
revelesi@uthscsa.edu

Regulatory Office:

Lisa Creighton
Mays Cancer Center at UT Health San Antonio
Institute for Drug Development
7979 Wurzbach Road, 4th floor
Mail Code# 8032
San Antonio, TX 78229
Tel. (210) 450-5953
Fax. (210) 450-0417
E-mail: creighton@uthscsa.edu
E-mail: RegulatoryAffairs@uthscsa.edu

INVESTIGATOR'S AGREEMENT

I have read and understand the contents of this clinical protocol for Protocol "A Phase 0, Investigator Initiated Study to Determine the Bioavailability of Sacituzumab Govitecan in Breast Brain Metastasis and Glioblastoma" CTMS# 19-0069 and will adhere to the study requirements as presented, including all statements regarding confidentially. In addition, I will conduct the study in accordance with current international conference on harmonization (ICH) guidance, Good Clinical Practice (GCP) guidance, the Declaration of Helsinki, US Food and Drug Administration (FDA) regulations and local IRB and legal requirements.

Name of Investigator: Andrew Brenner, MD, PhD

Institution: Mays Cancer Center, UT Health San Antonio

____________________________ __________________

Investigator Signature Date

TABLE OF CONTENTS

Synopsis	5
Introduction	8
Rationale	14
Objectives	14
Study Design	15
Population	22
Procedures	23
Materials and Supplies	27
Management of Intercurrent Events	29
Removing Subjects From Study	34
Conditions for Initiating, Modifying, or Terminating the Study	35
Investigator Responsibilities	35
References	42
Appendix A, Schedule	45
Appendix B, ECOG Performance Scale	46
Appendix C, RANO Response Criteria	46
Appendix D, Immunomedics “Product Inspection Form”	47

Synopsis

**Study Title:**	A Phase 0, Investigator Initiated Study to determine he bioavailability of Sacituzumab Govitecan in Breast Brain Metastasis and Glioblastoma
**Investigators:**	Andrew J. Brenner MD, PhD<br>John R. Floyd, MD<br>*See 1572 for other investigators*
**Indication**	Brain metastases from Breast cancer and Primary brain tumors (Glioblastoma)
**Primary Objectives:**	To determine the extent by which Sacituzumab Govitecan is able to penetrate the blood brain barrier and access tumor tissue by testing for free SN-38, SN-38G and total SN-38 concentrations in tumor tissue as well as in CSF (if available) and serum samples.
**Secondary Objectives:**	• To determine the progression-free survival after debulking craniotomy for patients treated with Sacituzumab Govitecan in patients with Breast brain metastatic tumors and Glioblastoma.<br>• To determine overall survival after debulking craniotomy for patients treated with Sacituzumab Govitecan in patients with Breast brain metastatic tumors and Glioblastoma.<br>• To assess the safety of Sacituzumab Govitecan in Breast brain metastatic tumors and Glioblastoma.
**Exploratory Objectives:**	Correlate total SN-38 and free SN-38 tumor tissue concentrations with tumoral hypoxia and tumoral Trop-2 expression.
**Study Design:**	Single center, non-randomized, Phase 0 study. Sacituzumab Govitecan given preoperatively, followed by craniotomy with surgery or biopsy of brain tumors (GBM and metastatic brain tumors from Breast) and intraoperative tissue collection will follow with contemporaneous CSF (depending on tumor location) and whole blood (serum) sampling.<br><br>Sacituzumab Govitecan treatment will be initiated on day-1, prior to surgery. Sacituzumab govitecan will continue to be administered by IV infusion over 3 hours on Days 1 and 8 of a 21 day cycle post-operatively until progression.<br><br>All patients will be subjected to standard dose of Sacituzumab Govitecan 10mg/kg without any dose escalations.

Cohorts	# of subjects	Post-operative Sacituzumab dose	Schedule/ Frequency	Route
1 (Breast brain metastasis)	10	10 mg/kg	D1 and D8 of 21-day cycle	Intravenous

	2 (Glioblastoma) | 10 | 10 mg/kg | D1 and D8 of 21-day cycle | Intravenous
**Duration:**	Subjects will be allowed to continue treatment on study post-surgery or until they have evidence of significant treatment-related toxicity or progressive disease.
**Planned Total Sample Size:**	Up to approximately 20 subjects: Two cohorts of patients will be enrolled in this phase 3 study, 10 subjects with breast brain metastasis and 10 subjects with Glioblastoma.
**Inclusion Criteria**	1. At least 18 years of age<br>2. Histologically or cytologically documented breast cancer (Cohort A) with known or suspected parenchymal brain metastases.<br>3. Recurrent glioblastoma (Cohort B) with documented progression by RANO criteria following standard combined modality treatment with radiation and temozolomide.<br>4. Plans to undergo craniotomy as part of standard of care. Patients emergently needing surgical debulking due to symptoms of their disease are not eligible.<br>5. Recovered from toxicities of prior therapy to grade 0 or 1<br>6. ECOG performance status ≤ 2.<br>7. Life expectancy of at least 3 months.<br>8. Acceptable liver function:<br>a) Bilirubin ≤ 1.5 times upper limit of normal<br>b) AST (SGOT) and ALT (SGPT) ≤ 3.0 times upper limit of normal (ULN);<br>9. Adequate renal function: calculated creatinine clearance ≥30mL/minute according to the Cockcroft and Gault formula<br>10. Acceptable hematologic status (without hematologic support)<br>a) ANC ≥1500 cells/uL<br>b) Platelet count ≥100,000/uL<br>c) Hemoglobin ≥9.0 g/dL<br>11. All women of childbearing potential must have a negative serum pregnancy test and male and female subjects must agree to use effective means of contraception (surgical sterilization or the use or barrier contraception with either a condom or diaphragm in conjunction with spermicidal gel or an IUD) with their partner from entry into the study through 6 months after the last dose.
**Exclusion Criteria:**	1. The subject is receiving warfarin (or other coumarin derivatives) and is unable to switch to low molecular weight heparin (LMWH) before the first dose of study drug.<br>2. The subject has evidence of acute intracranial or intratumoral hemorrhage either by MRI or computerized tomography (CT) scan. Subjects with resolving hemorrhage changes, punctate hemorrhage, or hemosiderin are eligible.<br>3. The subject is unable to undergo MRI scan (eg, has pacemaker).

	4. The subject has received enzyme-inducing anti-epileptic agents within 14 days of study drug (eg, carbamazepine, phenytoin, phenobarbital, primidone).<br>5. Patients whose only lesion undergoing resection has received stereotactic radiation within the past 3 months<br>6. The subject has received any of the following prior anticancer therapy:<br>• Biologic agents (antibodies, immune modulators, vaccines, cytokines) within 21 days prior to first dose of study drug<br>• Prior treatment with Sacituzumab Govitecan<br>7. Patients receiving UGT1A1 (Uridine diphosphate glucuronosyl transferase 1A1) inhibitors or inducers.<br>8. History of significant cardiovascular disease, defined as:<br>• Congestive heart failure greater than New York Heart Association (NYHA) Class II according to the NYHA Functional Classification.<br>• Unstable angina or myocardial infarction within 6 months before enrollment.<br>• Serious cardiac arrhythmia.<br>9. Clinically significant ECG abnormality, including:<br>• Marked Baseline prolonged QT/QTc interval (ie, a repeated demonstration of a QTc interval >500 ms) demonstrated on ECG at Screening.<br>• History of risk factors for torsade de pointes (eg, heart failure, hypokalemia, family history of long QT Syndrome).<br>10. Any medical or other condition which, in the opinion of the Investigator, causes the subject to be medically unfit to receive Sacituzumab Govitecan, or unsuitable for any other reason.
**Assessments of:**<br>• **Efficacy**	Progression-free survival<br>Overall survival
• **Safety**	Safety endpoints<br>• Incidence and severity of adverse events will be monitored using CTCAE version 5.0<br>• Changes in lab parameters, vital signs and weight
• **Tumor Penetration**	Tumor samples will be tested for total SN-38, free SN-38, and SN-38G with concentrations correlated against plasma concentration at the same timepoint.
**Procedures (Summary)**	All 20 subjects will receive study drug Sacituzumab Govitecan preoperatively. Intraoperative tissue collection will follow with contemporaneous CSF (depending on tumor location) and whole blood (serum) sampling. Samples will be tested for total SN-38and free SN-38, as well as SN-38G. Following recovery from surgery, patients will resume treatment<br><br>Adverse events will be reported for all events occurring after the start of treatment until 30 days after study drug is discontinued or subsequent cancer therapy is initiated.

INTRODUCTION

1.1 SCIENTIFIC BACKGROUND

1.1.1 Sacituzumab govitecan is an antibody drug conjugate that targets Trop-2 for the selective delivery of SN-38 to tumors. Trop2, also known as trophoblast antigen 2, is a cell surface glycoprotein which is differentially expressed in a number of epithelial tumors[1, 2]. Originally identified in trophoblast cells, it is an intracellular calcium signal transducer which provides crucial signals for cells with requirements for proliferation, survival, self-renewal, and invasion[3]. A number of approaches have been used to target Trop2, including antibody based therapy using the RS7-3G11 (RS7) murine IgG1 antibody[4][5]. In vitro studies have demonstrated antibody-dependent cellular cytotoxicity activity against Trop-2 positive carcinomas[6]. Based upon the broad reactivity of RS7 with epithelial cancers and its ability to internalize, Goldenberg et al hypothesized that conjugating RS7 to the topoisomerase inhibitor SN-38 (the active metabolite of irinotecan) could result in sustained release of the drug within the tumor environment as well as achieve direct intracellular delivery[7]. The antibody drug conjugate (ADC), Sacituzumab govitecan (SG), showed enhanced efficacy in rodent models with minimal toxicity in primates, leading to clinical trials. Most recently, SG was evaluated in a single-arm, multicenter trial in 69 patients with relapsed/refractory metastatic triple negative breast cancer (TNBC) at a 10 mg/kg starting dose on days 1 and 8 of 21-day cycles[8]. The results were impressive given the refractory nature of this population, with a confirmed objective response rate of 30% (including 2 complete responses), with responses occurring early (median onset of 1.9 months) and being durable (median duration 8.9m). SG has since been granted priority review designation by the FDA, with approval anticipated upon resolution of manufacturing issues. If approved, this will be the first targeted therapy for non-BRCA mutated TNBC.

Data has also been made available showing a similar level of efficacy in refractory hormone receptor positive breast cancer[9]. Fifty-four patients having received at least 2 prior treatments, with a median of 3 prior hormonal agents and 2 prior chemotherapy regimens were treated with SG. The overall response rate was 31% with 17 partial responses, and a clinical benefit rate of 48%.

1.1.2 Brain metastases remain a clinical dilemma in triple negative breast cancer. Nearly half of all women with advanced triple negative breast cancer will be diagnosed with brain metastases[10]. The outcome for these patients is quite poor, with a median overall survival following the diagnosis of brain metastasis of only 7.3 months [11]. Few treatment options exist, with only carboplatin and capecitabine being active agents in

triple negative breast cancer while also showing activity within the CNS. Even with these two therapies, systemic treatment has had no impact on overall survival following the diagnosis of brain metastasis for TNBC, in contrast to other subtypes such as luminal or HER2 subsets of patients[12]. Therefore, with the activity of SG in TNBC, and the high relative frequency of brain metastases in TNBC, a logical question follows as to SG's ability to reach and potentially impact brain metastases in TNBC.

1.1.3 Primary brain tumors are characterized by poor survival which correlates with Trop2 expression. In 2019, an estimated 26,170 new cases of primary malignant brain tumors will be diagnosed and 15,475 patients will die from these tumors[13]. The majority of these are astrocytic, with glioblastoma (GBM, Grade IV astrocytoma) representing 48%. GBM is the most common and most aggressive of the primary malignant brain tumors in adults, and hence the primary target of drug development for intracranial malignancy. Currently, front-line treatment consists of a multi-modality approach that includes maximal surgical resection, adjuvant radiation therapy with concurrent temozolomide, and maintenance temozolomide with tumor treatments fields[14]. Once a patient fails standard front-line therapy, prognosis is very poor. The only currently approved therapeutic for salvage treatment is bevacizumab, which has no proven survival benefit [15]. Survival for patients with GBM is currently only a median of 20.9 months. Interestingly, Trop2 expression correlates with not only grade in gliomas, but with malignant features. While normal brain expression of Trop2 is not observed, 95% of GBM samples examined showed moderate to intense staining by immunohistochemistry[16] and strong correlations were observed for both proliferation rate ($r=0.68$, $p=0.01$) and microvessel density ($r=0.37$, $p=0.03$). Associations have also been observed for both grade ($r=0.17$, $p<0.01$) and time to death ($r=-0.16$, $p<0.01$) in the TCGA dataset[17]. This supports GBM as an additional under-met need potentially targetable with SG, with the caveat that this ADC is able to reach its target.

1.1.4 Antibody drug conjugates may achieve higher concentrations within brain tumors than systemic administration of the drug alone. Consensus generated over the years has been that brain tumors, whether primary or secondary, are inaccessible to antibodies due to their large size and the restriction of the blood brain barrier (BBB). However, converging evidence has recently challenged this view. In breast cancer, the ADC Trastusumab-emtansine (T-DM1) was approved based upon a survival benefit in the EMILIA study[18]. The first suggestion of CNS activity was the finding of prolonged OS of 26.8 months in the 10% subset of patients with brain metastases treated with T-DM1 relative to patients treated with lapatinib/capecitabine with 12.9 months [19]. While prospective studies of intracranial response are otherwise lacking, these findings have been corroborated in retrospective analyses where the intracranial response rate has been seen at approximately 25% [20-22]. In GBM, encouraging data has been reported with the EGFR targeting ADC ABT-414, with an objective response rate of 14% and a 6-month progression free survival (PFS) rate of 25%. This was in a recurrent setting where responses are uncommon. Scott et al had previously shown accumulation of radiolabeled ^{111}In-ch806 (the parental chimeric precursor to ABT-414) within tumor of a patient with anaplastic astrocytoma at day 7 following infusion[23], with no accumulation in normal organs. Animal studies also lend support, with one study using labelled 81C6

demonstrating tumor-to-normal-brain ratios of 25:1 to as high as 200:1[24]. However, there is a clear lack of prospective studies characterizing intracranial intratumoral concentrations for ADCs.

1.1.5 Sacituzumab govitecan is unique as an ADC, with payload and linker characteristics preferable for CNS delivery. SG utilizes a linker designated CL2A (Figure 1). The linkage between CL2A and SN-38 is sensitive to both acidic and alkaline conditions, allowing the detachment of SN-38 at a rate of about 50% per the ex vivo serum study [25]. This less stable linker allows for SN-38 to be released at the tumor site after the ADC targets the cells, making the drug accessible to surrounding tumor cells and not just cells directly targeted by the ADC. The payload, SN-38, is the active metabolite of irinotecan which crosses the blood-brain barrier, and is frequently a drug partner in CNS regimens[26]. However, SN-38 has 1000 times more activity than irinotecan itself with typical IC50s in the single digit nanomolar range for most GBM cell lines[27]. The combination is one of a hydrolysable linker and release is not solely dependent on pH, with a payload that has high potency and good CNS penetration. While many ADCs may be dependent on disrupted vasculature to reach tumor antigen across the blood brain barrier and internalization for payload release, SG may additionally be able to release SN-38 within the vasculature upon encountering the reduced pH of the tumor microenvironment. Free SN-38, being able to cross the blood brain barrier, is thereby hypothetically capable of accumulating in the tumor.

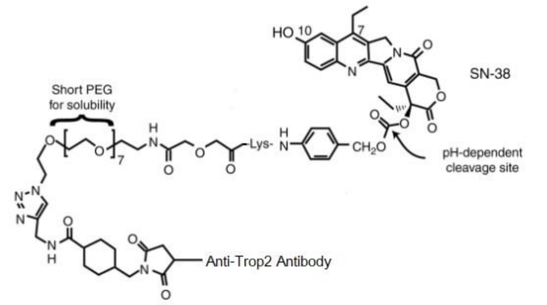

Figure 1. Sacituzumab govitecan structure with the CL2A linker providing a pH dependent cleavage site.

1.1.6 Nonclinical Studies with Sacituzumab Govitecan

1.1.6.1 Human Tumor Xenograft Studies

Sharkey et al [28] examined the delivery of SN-38 to Trop-2 expressing tumors and assessed constitutive products in serum, liver and small intestine in nude mice bearing human tumor xenografts, given a single injection of Irinotecan (40mg/kg; 0.8 mg/mouse, containing 460 µg SN-38 equivalents) or Sacituzumab Govitecan (SG); 1 mg containing 16 µg SN-38 equivalents).

Study design utilized 3 sets of studies for this analysis; nude mice bearing xenografts of the human pancreatic cancer cell line, Capan-1 and NCI-N87. Mice were administered intravenously with single doses containing fixed amount of SG (1.0mg), whereas Irinotecan was given at a dose of 40mg/kg, using the average prestudy weight of the animals to determine a fixed dose for that group. By mass, 1.0mg of irinotecan contains approximately 0.58mg of SN-38. Animals in the first and third study were examined at 5

different intervals and at 2 to 3 intervals in the second study. At select times, tissues were extracted and concentrations of the products measured by reversed-phase high-performance liquid chromatography (HPLC)[28]. HPLC analysis of SN-38, SN-38G and irinotecan required extractions of the harvested tissues.

This study demonstrated the following results favoring Sacituzumab govitecan (IMMU-132). In serum, >98% of irinotecan was cleared within 5 minutes ; peak levels of SN-38 and SN-38G (glucuronidated SN-38) were detected in equal amounts and no longer detected after 6 to 8 hours. Intact IMMU-132 cleared with a half-life of 14 hours which closely reflected the in vitro rate of SN-38 released from the conjugate in mouse serum[28]. Area under the curve (AUC) analysis indicated that IMMU-132 delivers 20-fold to as much as 136-fold more SN-38 to tumors with irinotecan, with tumor: blood ratios favoring IMMU-132 by 20- to 40- fold. Intestinal concentrations of SN-38/SN-38G also were 9-fold lower with IMMU-132, indicating reduced intestinal uptake that elucidates the lower rate and severity of diarrhea in patients[28].

SN-38 is a highly potent drug but delivery by irinotecan is compromised by (i) rapid clearance from the blood, (ii) poor conversion rate, and (iii) rapid conversion to the inactive SN-38G form. Thus, development of new type of ADC that utilizes SN-38, improves the pharmacokinetics of SN-38 and also provided a mechanism for selective tumor retention via the retention antibody. IMMU-132 delivers much higher levels of SN-38 to tumors than irinotecan and importantly, all of the SN-38 delivered to the tumor by IMMU-132 is released in its most potent form along with significantly much lower amounts of SN-38/SN-38G in the intestine reduces the risk of severe diarrhea in patients , which is confirmed in clinical studies[28].

1.1.7 Clinical Study with Sacituzumab govitecan

Study reported by Starodub et al 2015[29] is a phase 1 dose escalation study investigating Sacituzumab govitecan in subjects with metastatic solid cancers. The trial was designed as a 3 + 3 phase 1 design, starting at a dose of 8mg/kg per injection, with dosages given weekly for 2 weeks (day1 and 8) in a 3-week treatment cycle, with cycles repeated until dose-limiting toxicity or progression. Although no preselection based on Trop-2 expression was required, Trop-2 expression in present in >75% of the cases. Twenty five patients were treated at dose levels of 8 (n=7), 10 (n=6), 12 (n=9) and 18 (n=3)mg/kg. Based on the toxicity studies, hematologic toxicity was reported to be the major dose-limiting adverse reaction with grade 3 or 4 neutropenia (n=9) at doses of 12 and 18mg/kg. While the maximum tolerated dose (MTD) was declared to be 12mg/kg, 8.0 and 10.0 mg/kg dose levels were selected for further expansion, as patients were more likely to tolerate additional cycles at these levels with minimal supportive care and responses were observed at these levels. Other common toxicities attributed to sacituzumab govitecan included fatigue, nausea, vomiting (grade 1 or 2) and alopecia. This phase 1 experience has indicated that SG is tolerated with moderate and manageable toxicity, all related to the activity of SN-38 with no evidence of damage to normal tissues known to contain Trop-2. Importantly, SG is active in patients with diverse metastatic solid tumors, even after failing prior therapy with topoisomerase-1 inhibitors.

Ocean et al. 2017 reported preliminary results of the Phase I/II multicenter, dose expansion study to determine the pharmacokinetics and safety of multiple cycles of Sacituzumab govitecan (IMMU-132) at doses of 8 or 10 mg/kg in patients with diverse advanced epithelial cancers[30]. Patients with diverse metastatic cancers who received IMMU-132 at 8mg/kg (n=81) and 10mg/kg (n=97) were examined. The median number of prior therapies for all patients was four. Trop-2 was positive in 93% of the available specimens. Hematological toxicity with grade 3 or 4 neutropenia was observed at both doses causing treatment delays (29% in 8mg/kg group and 34% in 10mg/kg dose group)[30]. 50% of all dose reductions in both the groups occurred after first or second dose in the first cycle. During the phase 2 expansion portion of the trial, investigators were permitted to use a granulocyte - colony - stimulating factor cytokine after the first dose to prophylactically control subsequent neutropenia, with 22% in 8mg/kg group and 26% in 10mg/kg group requiring one hematologic cytokine supportive treatment[30]. Patients with UGT1A1*28*28 haplotype were somewhat more likely to develop grade >/=grade 3 neutropenia than those with other haplotypes; however, because 40% of patients did not experience grade>/=3 neutropenia, and approximately 40% of those with other haplotypes experienced grade>/=3 neutropenia, the management of neutropenic event is more appropriate than screening for patients who have UGT1A1 mutant alleles[30]. At the time of analysis, adverse events for all causalities were reported as 99% and 92% in the 8mg/kg and 10mg/kg cohorts. The 10mg/kg cohort had somewhat higher incidence of grade 3 events including neutropenia, febrile neutropenia and diarrhea, but the overall incidences were not appreciable to warrant selecting 8mg/kg as the starting dose. Free SN-38 levels 30 minutes after the first dose and neutropenia were assessed which showed that there is no evidence to suggest that neutropenia was correlated with the levels of free SN-38 initially in the serum. No serum samples exhibited any antibody response to any component of IMMU-132. In addition, the clinical benefit rate was calculated by combining objective response rates and stable disease that lasted for >/=4months for lung cancer and >/=6months for colorectal and TNBCs, was higher in 10mg/kg group than the 8mg/kg cohort. In conclusion, results from this trial showed that Sacituzumab govitecan is safe with excellent pharmacokinetic profile, manageable toxicity profile along with improved responses and a good therapeutic index at 10mg/kg, making this dose being chosen for future developments particularly in metastatic TNBC for pursuing accelerated approval.

Study reported by Bardia et al. 2017 is a single arm, multicenter study that enrolled patients with many different cancer types (including mTNBC) to evaluate safety and antitumor activity of Sacituzumab in these patients. 69 patients with mTNBC were enrolled, all were heavily pretreated with median of five lines of therapy since diagnosis and were in the dosing cohort of 10mg/kg, administered intravenously on days 1 and 8 of 21-day repeated cycles[31]. The primary end points were safety and objective response rate (ORR); secondary end points were progression-free survival and overall survival. The confirmed ORR was 30% with median response duration of 8.9 months and early median onset of response was 1.9 months; clinical benefit rate was at 46%[31]. Median Progression free survival (PFS) was 9.6(95% CI, 5.0 to 7.3) months and median overall survival was 16.6 (95% CI, 11.1 to 20.6) months, along with manageable side effect

profile[31]. This received Breakthrough Therapy designation from the FDA for the treatment of patients with mTNBC.

1.1.8 Standard Treatment for High Grade Glioma

High-grade gliomas are highly aggressive tumors and invariably recur after standard of care first-line therapy. Currently, front-line treatment consists of a multi-modality approach that includes maximal surgical resection, adjuvant radiation therapy with concurrent temozolomide at 75 mg/m^2 followed by 6 months of single-agent temozolomide at up to 200 mg/m^2. Temozolomide (a prodrug) is a rapidly and non-enzymatically converted to the active alkylating metabolite MTIC [(methyl-triazene-1-yl) – imidazole-4-carboxamide]. The cytotoxic effects of MTIC are manifested through alkylation of DNA at the O6, N7 guanine positions.

This multimodal approach has been the standard of care ever since the publication of the landmark EORTC phase III trial published by Roger Stupp et al in 2005[32] that demonstrated an improved primary end-point of median overall survival of this regimen when compared to adjuvant radiation alone. A median survival of 14.6 months was reported in the temozolomide group versus 12.1 months in the radiation alone group[32]. At that time, radiation alone was considered standard of care in most countries. In the US, common practice was to add adjuvant nitrosurea-based chemotherapy regimen with a small survival benefit with significant adverse effects.

However, once a patient fails standard front-line therapy, prognosis is very poor and new therapies are needed. In the era of targeted therapies, anti-angiogenic drugs have come into the limelight. Bevacizumab, a recombinant humanized monoclonal antibody against VEGF, has been studied extensively over the past several years and has demonstrated rather impressive radiographic and clinical response rates when compared to historical data but the responses are not very long-lasting. The largest study to date was a phase II, multicenter, open-label, noncomparative trial conducted by Friedman et al in 2009[33] which evaluate the efficacy of bevacizumab (10 mg/kg every 2 weeks), alone and in combination with irinotecan (125 mg/m2 in those non receiving enzyme-inducing anti-epileptic agents and 340 mg/m2 in those who were taking these drugs), in patients with recurrent glioblastoma. In the bevacizumab-alone and the bevacizumab-plus-irinotecan groups, estimated 6-month progression-free survival rates were 42.6% and 50.3%, respectively; objective response rates were 28.2% and 37.8%, respectively; and median overall survival times were 9.2 months and 8.7 months, respectively. Nonetheless, these are all improved outcomes when compared to historical data in the recurrent setting. Bevacizumab obtained accelerated FDA-approval in 2009 for patients with recurrent glioblastoma multiforme. The activity of Sacituzumab govitecan has not yet been assessed in GBM. In order to address CNS penetration of sacituzumab govitecan in these settings, we propose a window of opportunity surgical study to assess bioavailability of Sacituzumab govitecan.

1.1.9 Standard Treatment of Breast Brain Metastasis

Breast cancer is the most frequently diagnosed tumor and second leading mortality in female world[34]. It is the second most common solid malignancy to metastasize to brain, estimated to be present at the time of diagnosis of breast cancer in 0.41% of patients,

constituting all 7.56% of all metastatic sites [35]. Patients with triple negative or HER2 positive subtypes experience significantly higher incidence of brain metastasis (BM) occurrence. The cumulative incidence of brain metastasis at 1 and 2 years was 17 and 25% respectively[11]. Retrospective series illustrate overall survival following a diagnosis of TNBC-BM is 4 months despite initial treatment with whole brain radiotherapy (WBRT)[36]. In contrast to patients with endocrine sensitive or HER2-positive BC-BM, the addition of systemic therapy following WBRT has yet to yield improvements in survival for patients with TNBC-BM. The development of effective systemic therapies for patients diagnosed with TNBC-BM is an urgent and unmet medical need. Phase II study of Inipraib in combination with Irinotecan/TBCRC 018 evaluated the efficacy and safety of Iniparib which is a small molecule anti-cancer agent that crosses the blood brain barrier, with the topoisomerase I inhibitor irinotecan in patients with TNBC-BM. This novel, phase II study illustrated that irinotecan and iniparib yields an intracranial clinical benefit rate (CBR) of 30% among patients with new and/or progressive TNBC-BM with a tolerable safety profile and no significant detriment to quality of life[37]. While encouraging, further treatment options are clearly needed.

1.1.10 Biomarkers

As mentioned above, SG targets Trop-2 for the selective delivery of SN-38 to tumors. SG carries SN-38, a topoisomerase inhibitor active in the nanomolar range for most cells and freely cross the blood brain barrier. Brain metastases is a significant concern in mTNBC, but whether this agent is able to target the CNS through the blood brain barrier is unknown. We further hypothesize that while total concentration of SN-38 (both antibody conjugated and free) will correlate with expression of trop2, free SN-38 will correlate more strongly with intratumoral hypoxia. We will assess carbonic anhydrase IX (CAIX), Trop-2, and γ-H2AX in surgical specimens by immunohistochemistry, semiautomated image analysis and semiquantitative scoring will be performed, and correlated to results from the above aim.

1.2 RATIONALE FOR DOSE SELECTION AND SCHEDULE

Dosing will be at 10 mg/kg on days 1 and 8 of a 21-day cycle, as previously published and as being assessed for accelerated approval in metastatic TNBC.

1.3 STUDY COMPLIANCE

This study will be conducted in compliance with this protocol, the principles of Good Clinical Practices, and applicable regulations.

2. OBJECTIVES OF THE STUDY

2.1 PRIMARY OBJECTIVES

To determine the extent by which Sacituzumab Govitecan is able to penetrate the blood brain barrier and access tumor tissue by testing for free SN-38, SN-38G and total SN-38 concentrations in tumor tissue as well as in CSF (if available) and serum samples.

2.2 SECONDARY OBJECTIVES

1. To determine the progression-free survival after debulking craniotomy for patients treated with Sacituzumab Govitecan in patients with breast brain metastatic tumors and glioblastoma.
2. To determine overall after debulking craniotomy for patients treated with Sacituzumab Govitecan in patients with breast brain metastatic tumors and glioblastoma.
3. To assess the safety of Sacituzumab Govitecan in Breast brain metastatic tumors and Glioblastoma.

2.3 EXPLORATORY OBJECTIVES

1. Correlate tumoral total SN-38 and free SN-38 with tumoral hypoxia and tumoral Trop-2 expression.

3. STUDY DESIGN AND METHODS

3.1 DESIGN

1. Single center, prospective study, non-randomized
2. Sacituzumab govitecan is given as single dose at 10mg/kg administered pre-operatively on Day-1 and will receive sacituzumab govitecan post-operatively on Day 1 and 8 of 21-day cycle until progression.
3. Sacituzumab govitecan will be administered intravenously over a period of 3 hours at a dose of 10mg/kg.
4. Surgery will be followed by post-operative treatment with sacituzumab govitecan given intravenously with standard dose of 10 mg/kg on day1 and day 8 of 21-day cycle, until disease progression

Cohort	# of subjects	Pre-op dose	Post- op dose (Day1 and Day8 of 21-day cycle)
1 (GBM)	10	10mg/kg	10mg/kg
1 (Breast brain tumors)	10	10mg/kg	10mg/kg

5. Approximately 20 patients will be enrolled. Study includes 2 cohorts of 10 patients each with GBM and breast brain tumors from TNBC (n=10 in each cohort).
6. Sacituzumab govitecan will be administered by IV infusion over 3 hours on day 1 and day 8 of each 21-day cycle.

3.1.1 Investigational Sites

This study is a single center, Phase 0 study, non-randomized without dose escalation.

3.1.2 Subject Assignment Methods

This is an open-label study and all of the patients enrolled will receive the study drug Sacituzumab Govitecan pre-operatively and post-operatively. Study includes two cohorts

of patients, which includes patients with Glioblastoma multiforme and metastatic breast brain tumors. Presurgical dose will consist of single agent Sacituzumab Govitecan at 10mg/kg IV prior to surgery. Once the patient has recovered from surgery adequately in the opinion of both the PI and the neurosurgeon, and if they continue to meet all eligibility criteria, they will be eligible to continue receiving Sacituzumab govitecan in the post-operative setting.

3.1.3 Statistics

Given the absence of previous data on study drug uptake in human tumors and probable heterogeneity, no formal sample size calculation was conducted. Descriptive statistics will be used to summarize and compare drug concentrations in serum, CSF and tumor tissue. Drug levels from resected specimens will be presented as averages and ranges of determinations, with correction for blood volume using the absorbance of hemoglobin. Tumor concentrations will similarly be expressed as a tumor-to-serum ratios. For serum studies, area-under-the-curve (AUC) concentration will be calculated, ending at the time of tumor resection. The numbers of patients screened, screen failures by reason, the number enrolled and completing the study at each stage and the number and proportion progression-free at each stage will be tabulated. The distribution of time to progression and death will be summarized with Kaplan-Meier curves. Adverse events will be tabulated.

For biomarker exploration, two means of analysis will be used. First, a traditional semiquantitative analysis by a trained pathologist utilizing the H-score. The score is obtained by the formula:

> 3 x percentage of strongly staining nuclei + 2 x percentage of moderately staining nuclei + percentage of weakly staining nuclei, giving a range of 0 to 300.

Additionally, semiautomated image analysis and semiquantitative scoring will be performed using Image ProPlus Software. The hypoxic fraction is calculated as the ratio of area of the region of interest identified with morphometric analysis to total area of the representative area analyzed. The analyses between immunohistochemistry and drug concentration correlation will be calculated by linear regression.

All statistical testing will be two sided with a significance level of 5%. SAS Version 9.3 for Windows (SAS Institute, Cary, North Carolina) will be used throughout. Statistical analyses will be performed by Joel E. Michalek, PhD, Department of Epidemiology and Biostatistics University of Texas Health Science Center at San Antonio.

3.2 TREATMENT REGIMEN

3.2.1 Sacituzumab Govitecan

The dose SG chosen for this study is 10mg/kg consistent with published results [8, 29, 30, 38]. Sacituzumab govitecan will be administered by IV infusion over 3 hours. Each dose will be prepared in normal saline for injection in infusion bags and administered

intravenously via an infusion pump. The full recommended infusion volume should be used. Each 100 mg of antibody contains approximately 1.6 mg of SN-38, with a mean drug:antibody ratio (DAR) of 7.6:1[29]. Before the start of each infusion, prophylaxis with acetaminophen 650mg po, diphenhydramine 25mg IV, and dexamethasone 10mg is given. Presurgical treatment will be allocated to a single dose either 16 hours preoperatively (t1/2, n=5 per cohort) or 48 hours preoperatively (3x t1/2, n=5 per cohort). These time points were selected based upon the established half-life[30] and results from subcutaneous xenograft studies[29]. Growth factor support (G-CSF, filgrastim, peg-filgrastim) is mandated for the preoperative dose, and at the investigator's discretion for subsequent doses. Following recovery from surgery, therapy consists of two consecutive doses given on days 1 and 8 of a 3-week treatment until unacceptable toxicity or progression. Timing between consent and preoperative dose, as well as resuming dosing postoperatively are as noted in section 5.3.

This study will use a standard dose of 10mg/kg IV in both the cohorts (no dose escalations).

Cohort	# of subjects	Pre and post -operative Sacituzumab dose
1	10	10mg/kg
2	10	10mg/kg

Intravenous Administration of sacituzumab govitecan
Do not administer as an IV push or bolus. Sacituzumab Govitecan is administered intravenously as a slow infusion as described below.

Intravenous access must be well established prior to initiating infusion. At the time of dosing, the IV line will be connected to an infusion container containing the prepared volume of Sacituzumab Govitecan. Either gravity or an infusion pump may be used. Only normal saline should be used as the infusion base solution, since the Sponsor has not examined the compatibility of Sacituzumab Govitecan with other infusion diluents.

The initial infusion should proceed slowly. If vital signs remain stable and in the absence of infusion reactions, the infusion rate can be incrementally advanced following suggested guidelines given in the table below, and following completion, the intravenous line should be flushed slowly with 20 mL normal saline and the end of infusion time recorded. In the event of infusion reactions or vital sign changes, the infusion rate may be slowed, interrupted or terminated, as considered appropriate by the managing physician.

Study drug must be stored under refrigerated conditions (2-8°C) in a locked room that can be accessed only by the pharmacist, the study Investigator, or another duly authorized study/site personnel. The study medications must not be used outside of the context of this protocol. Under no circumstances should the Investigator or other site personnel supply study drug to other Investigators, subjects, or clinics, or allow supplies to be used other than as directed by this protocol without prior written authorization from Immunomedics, Inc.

Table 3.2.1 Infusion Rate Guidelines for Patients Remaining Stable in Absence of Hypersensitivity or Infusion-Related Events

Infusion Rate	Infusion #1	Subsequent Infusions
Initial Rate (first 15 min.)	50 mg/hr, or less	100 - 200 mg/hr
Incremental Rate (advance every 15-30 min.)	50 mg/hr	100 - 200 mg/hr
Maximum Recommended Rate	500 mg/hr	1000 mg/hr

Drug Interactions

No formal drug-drug interaction studies with Sacituzumab Govitecan have been conducted. SN-38 (the active metabolite of Sacituzumab Govitecan) is metabolized via human UDP-glucuronosyltransferase 1A1(UGT1A1). Concomitant administration of strong inhibitors or inducers of UGT1A1, with Sacituzumab Govitecan, should be avoided due to the potential to either increase (inhibitors) or decrease (inducers) the exposure to SN-38.

3.3 DOSE MODIFICATIONS AND TREATMENT OF SACITUZUMAB GOVITECAN – ASSOCIATED TOXICITIES

Instructions for the infusion of sacituzumab govitecan are provided in Section 6.2. The following sections provide guidance for sacituzumab govitecan administration and management of treatment-related toxicities, including modification of dosing and treatment discontinuation. Toxicities should be managed in accordance with standard institutional practices and accepted treatment guidelines.

3.3.1 Preventative Medications

Infusion-Related Reactions: Pre-medication for prevention of infusion-related reactions with antipyretics and H1 and H2 blockers should be administered before each sacituzumab govitecan infusion. Corticosteroids (hydrocortisone 50 mg or equivalent P.O. or I.V.) may be administered prior to subsequent infusions if the patient had experienced an infusion-related reaction with a previous infusion. Additional details of recommended treatment of infusion-related reactions are described in Section 3.3.2.1.

Nausea, Vomiting: Sacituzumab govitecan is considered to be moderately emetogenic. Premedication with a 2-drug antiemetic regimen is recommended. If nausea and vomiting are persistent, a 3-drug regimen may be used, including a 5-HT3 inhibitor (ondansetron or palonosetron, or other agents according to local practices), an NK1-receptor antagonist (fosaprepitant), and dexamethasone (10 mg PO or IV). The use of fosaprepitant is preferred over aprepitant as aprepitant is a cytochrome P450 3A4 inhibitor and, therefore, may increase exposure to sacituzumab govitecan. Anticipatory nausea can be treated with olanzapine. The recommended treatment of delayed nausea and vomiting is described in Section 6.5.2.2.

3.3.2 Management of Sacituzumab Govitecan Toxicities

NCI-CTCAE v5.0 is used to grade the severity of all AEs. The guidelines for management of toxicities associated with sacituzumab govitecan are based on the assessment of severity according to these criteria. Toxicities should be managed in accordance with standard medical practice and treatment guidelines. Instructions for sacituzumab govitecan dose reduction for treatment-related toxicities are provided in Section 3.3.2.2.

3.3.2.1 Infusion-Related Reactions

Infusion-related reactions are defined as symptoms that occur during and within the first 6 hours after the infusion of sacituzumab govitecan. Symptoms can include: fever, chills, rigors, arthralgias, myalgias, urticaria, pruritus, rash, diaphoresis, hypotension, dizziness, syncope, hypertension, dyspnea, cough, and wheezing, as well as severe hypersensitivity reactions including anaphylactic reactions. Infusion-related reactions should be treated in accordance with best clinical practices and standard institutional guidelines. Because of the potential for life-threatening infusion-related reactions, sacituzumab govitecan should only be administered in a setting in which appropriately trained medical staff, emergency equipment and medications are available in the event that resuscitation is required. NCI CTCAE v5.0 is used to grade the severity of all infusion-related adverse events. Premedication for the prevention of infusion-related reactions is described in Section 3.3.1.

Grade 3 and Grade 4 Events

Grade 3 and Grade 4 infusion-related reactions can include severe or clinically significant cardiopulmonary events and severe allergic reactions such as symptomatic bronchospasm and anaphylactic reactions. Grade 3 infusion-related reactions are defined as those which are prolonged and do not improve with symptomatic treatment and/or brief interruption of treatment, reactions that recur following treatment, and reactions that require hospitalization. Grade 4 reactions include potentially life-threatening reactions, requiring urgent intervention. Severe allergic and anaphylactic reactions should be treated in accordance with best clinical practices and standard institutional guidelines. If Grade 3 or Grade 4 infusion-related reactions occur, sacituzumab govitecan should be permanently discontinued.

Grade 2 Events

Grade 2 infusion-related reactions are defined as those that require infusion interruption and respond to symptomatic treatment; prophylactic medications are indicated for ≤24 hours. For Grade 2 infusion-related reactions, the infusion should be interrupted for at least 15 minutes until symptoms resolve. After symptoms resolve, the infusion should be resumed at a slower infusion rate. Recommended infusion rates are provided in Section *<insert cross-reference to section that describes drug administration>*. For recurrent Grade 2 infusion reactions that fails to recover within 6 hours, despite optimal management, permanently discontinue sacituzumab govitecan.

3.3.2.2 Gastrointestinal Toxicities

Nausea, vomiting, and diarrhea are frequent sacituzumab govitecan-associated toxicities. Appropriate treatment, including, as needed, fluid and electrolyte replacement, is required to minimize the risk of serious consequences such as dehydration. Instructions for

sacituzumab govitecan dose reduction for treatment-related gastrointestinal toxicities are provided in Section 3.3.2.4.

Nausea and Vomiting
Instructions for the use of premedications for prophylactic treatment of nausea and vomiting and anticipatory nausea are provided in Section 6.5.1. Do not hold the dose of sacituzumab govitecan for Grade 3 nausea unless Grade 3 nausea persists despite maximal optimal medical management. Patients should be treated for delayed nausea and vomiting on Days 2 and 3 with 5-HT_3 receptor antagonist (ondansetron or palonosetron) monotherapy and other agents if needed. Steroids may be added if symptoms do not resolve with these other agents. Consider olanzapine for persistent or anticipatory nausea; an olanzapine dose of 2.5 mg or 5 mg at bedtime is recommended. If a NK1 receptor antagonist is administered, the use of fosaprepitant is preferred over aprepitant as aprepitant is a cytochrome P450 3A4 inhibitor and, therefore, may increase exposure to sacituzumab govitecan.

Diarrhea
Dietary modification should be recommended for the management of diarrhea., including a bland diet, small frequent meals, adequate fluid intake of clear liquids to maintain hydration, and discontinuation of lactose-containing foods and drinks and alcohol. Loperamide should be administered at the onset of treatment-related Grade 1 or Grade 2 diarrhea, at an initial dose of 4 mg, followed by 2 mg with every episode of diarrhea to a maximum dose of 16 mg/day. If diarrhea is not resolved after 24 hours,—add diphenoxylate/atropine.

Add octreotide 100-150 mcg SC tid if diarrhea persists. For Grade 3 or Grade 4 diarrhea, the patient should be hospitalized, and treated with intravenous fluids, and octreotide. Antibiotics can be administered as clinically indicated.

Subjects who exhibit an excessive cholinergic response to treatment with sacituzumab govitecan (e.g., abdominal cramping, diarrhea, salivation, etc.) can receive appropriate premedication (e.g., atropine) for subsequent treatments.

3.3.2.3 Neutropenia

Complete blood cell counts must be obtained prior to each sacituzumab govitecan infusion and should be administered if absolute neutrophil counts meet the following criteria:

- Day 1: ANC > 1500/mm^3
- Day 8: ANC >1000/mm^3

The routine prophylactic use of growth factors is not recommended; however, they may be used in patients who have experienced febrile neutropenia or Grade 3 or Grade 4 neutropenia following previous infusions. Growth factors may also be administered in the setting of neutropenia in patients at high risk of poor clinical outcomes, including those with prolonged neutropenia, ANC <100/mm^3, febrile neutropenia, and serious infections.

3.3.2.4 Sacituzumab Govitecan Dose Delays, Dose Reductions and Discontinuation Guidelines

Dose Delays

Sacituzumab govitecan is to be administered in 21-day cycles on day 1 and day 8; the next cycle should start a minimum 14 days after the day 8 dose. Visit windows of one day prior to and two days after the scheduled infusion are permitted. The scheduled day 1 and day 8 infusions may be delayed for up to one week for treatment-related toxicities. Instructions for dose delays and dose reductions for specific toxicities are summarized below. Dosing may be delayed for >Grade 2 toxicities for a maximum of one week. If the toxicity has improved to ≤Grade 2, the dose should be administered at that time. For a toxicity that delays day 8 dosing, if the toxicity has not resolved to ≤Grade 2 within one week, dosing should resume with the next scheduled cycle, i.e. the next dose will be Day 1 of the following cycle. Regardless of whether the day 8 dose is delayed for toxicity, there should be a minimum of 14 days between the day 8 infusion and the day 1 infusion of the next cycle. There cannot be more than a 5-week dose delay from the last dose or failure to resolve a toxicity within 3 weeks from the last dose.

Dose Reductions and Discontinuation

The major toxicities of sacituzumab govitecan are expected to be gastrointestinal symptoms and hematologic suppression. All patients will be closely monitored over the course of their treatment and aggressively medically managed, including dose reduction and interruption, in order to prevent the need for treatment discontinuation and serious complications of these toxicities. Sacituzumab dose reductions and interruptions will be managed based on toxicity severity, as assessed by NCI CTCAE v5.0. The sacituzumab govitecan dose must not be re-escalated following a dose reduction. Table 1 summarizes recommendations for sacituzumab govitecan dose reductions and discontinuations for treatment-related toxicities.

Table 1: Recommended Dose Reduction Schedule for Sacituzumab Govitecan

**Event**<br>NCI CTCAE v5.0	**Occurrence**	**Recommended dose reduction or action**
**Severe Neutropenia**		
Grade 4 neutropenia ≥7 days,<br>OR<br>Grade 3 febrile neutropenia (absolute neutrophil count <1000/mm^3 and fever ≥38.5°C),<br>OR<br>At time of scheduled treatment, ≥Grade 3 neutropenia which has delayed dosing by 1 week	First	Administer granulocyte-colony stimulating factor (G-CSF) or sooner, if clinically indicated
	Second	25% dose reduction
	Third	50% dose reduction
	Fourth	Discontinue treatment

≥Grade 3 neutropenia which delays dosing beyond 3 weeks	First	Discontinue treatment
**Severe Non-Neutropenic Toxicity**		
Grade 4 non-hematologic toxicity of any duration,<br>OR<br>Any ≥Grade 3 nausea, vomiting or diarrhea due to treatment that is not controlled with antiemetics and anti-diarrheal agents,<br>OR<br>Other ≥Grade 3 non-hematologic toxicity persisting >48 hours despite optimal medical management,<br>OR<br>At time of scheduled treatment, ≥Grade 3 non-neutropenic hematologic or non-hematologic toxicity, which has delayed dosing by 1 week	First	25% dose reduction
	Second	50% dose reduction
	Third	Discontinue treatment
≥Grade 3 non-neutropenic hematologic or non-hematologic toxicity, which has delayed dosing for more than 3 weeks	First	Discontinue treatment

4. PATIENT POPULATION

4.1 INCLUSION CRITERIA

1. At least 18 years of age
2. Histologically or cytologically documented breast cancer (Cohort A) with known or suspected parenchymal brain metastases.
3. Recurrent glioblastoma (Cohort B) with documented progression by RANO criteria following standard combined modality treatment with radiation and temozolomide.
4. Plans to undergo craniotomy as part of standard of care. Patients emergently needing surgical debulking due to symptoms of their disease are not eligible.
5. Recovered from toxicities of prior therapy to grade 0 or 1
6. ECOG performance status ≤ 2.
7. Life expectancy of at least 3 months.
8. Acceptable liver function:
    a) Bilirubin ≤ 1.5 times upper limit of normal
    b) AST (SGOT) and ALT (SGPT) ≤ 3.0 times upper limit of normal (ULN);
9. Acceptable renal function: calculated creatinine clearance ≥30mL/minute according to the Cockcroft and Gault formula
10. Acceptable hematologic status (without hematologic support)
    a) ANC ≥1500 cells/uL
    b) Platelet count ≥100,000/uL
    c) Hemoglobin ≥9.0 g/dL

12. All women of childbearing potential must have a negative serum pregnancy test and male and female subjects must agree to use effective means of contraception (surgical sterilization or the use or barrier contraception with either a condom or diaphragm in conjunction with spermicidal gel or an IUD) with their partner from entry into the study through 6 months after the last dose

4.2 EXCLUSION CRITERIA

1. The subject is receiving warfarin (or other coumarin derivatives) and is unable to switch to low molecular weight heparin (LMWH) before the first dose of study drug.
2. The subject has evidence of acute intracranial or intratumoral hemorrhage either by MRI or computerized tomography (CT) scan. Subjects with resolving hemorrhage changes, punctate hemorrhage, or hemosiderin are eligible.
3. The subject is unable to undergo MRI scan (eg, has pacemaker).
4. The subject has received enzyme-inducing anti-epileptic agents within 14 days of study drug (eg, carbamazepine, phenytoin, phenobarbital, primidone).
5. Patients whose only lesion undergoing resection has received stereotactic radiation within the past 3 months
6. The subject has received any of the following prior anticancer therapy:
   a) Biologic agents (antibodies, immune modulators, vaccines, cytokines) within 21 days prior to first dose of study drug
   b) Prior treatment with Sacituzumab Govitecan
7. Patients receiving UGT1A1 (Uridine diphosphate glucuronosyl transferase 1A1) inhibitors or inducers.
8. History of significant cardiovascular disease, defined as:
   i) Congestive heart failure greater than New York Heart Association (NYHA) Class II according to the NYHA Functional Classification.
   ii) Unstable angina or myocardial infarction within 6 months before enrollment.
   iii) Serious cardiac arrhythmia.
9. Clinically significant ECG abnormality, including:
   i) Marked Baseline prolonged QT/QTc interval (ie, a repeated demonstration of a QTc interval >500 ms) demonstrated on ECG at Screening.
   ii) History of risk factors for torsade de pointes (eg, heart failure, hypokalemia, family history of long QT Syndrome).
10. Any medical or other condition which, in the opinion of the Investigator, causes the subject to be medically unfit to receive Sacituzumab Govitecan, or unsuitable for any other reason.

5. PROCEDURES

Subjects are expected to participate for up to treatment duration with continued follow up for survival until one year after the first dose of study drug. Please refer to Appendix A, Schedule of Assessments, for an overview of the study assessments. Subjects who withdraw from the study before all follow-up procedures have been performed will be managed and documented as described in Section 8, Removing Subjects from the Study.

A summary of visits and clinical procedures is found in Appendix A, Schedule of Assessments. The total duration of the active part of the study for each subject will be approximately 18 weeks, divided as follows:

- Up to 3 weeks predose (screening period)
- Up to 6 weeks for presurgical dosing, surgery and recovery.
- 3-week treatment periods of Sacituzumab govitecan
- Study termination visit 1-2 weeks after last dose of study medication

When a subject has completed the study termination or early termination visit, he/she and/or a family member will be contacted for survival information every 3 months until one year from completion.

All subjects will be screened within 21 days prior to Presurgical Day -1. Vital signs, clinical laboratory test results, weight and AEs will be used to assess safety. Efficacy will be assessed based on tumor assessments (objective response rate, progression-free survival and duration of response) conducted at intervals during the study. Subjects who have not progressed after 6 cycles may be permitted to continue therapy on a case-by-case basis.

During screening, candidates for the study will be fully informed about the nature of the study and possible risks, and will receive a copy of the informed consent for review. Candidates must read the consent form and sign the document after the investigator has answered all questions to the candidate's satisfaction. Further procedures can begin only after the consent form has been signed. The original signed consent form will be retained by the investigator and a copy will be given to the candidate. Candidates will be evaluated for entry into the study according to the stated inclusion and exclusion criteria (Section 4, Study Population). The investigator will evaluate the results of all examinations, including clinical laboratory tests, and will determine each candidate's suitability for the study. The investigator must know the baseline results before enrollment. The pregnancy test for females of reproductive potential must be negative for those subjects to proceed to enrollment. All screening procedures must be done within 21 days of presurgical day -1, unless otherwise specified. The following procedures will be performed to establish each candidate's general health and qualifications for possible enrollment into the study:

- Obtain signed, written informed consent and permission to use protected health information, (in accordance with the Health Insurance Portability and Accountability Act or HIPAA). Refusal to sign informed consent and permission excludes an individual from the study.
- Record medical history, including cancer history: histology of primary tumor (including degree of differentiation), date of cancer diagnosis, types and dates of prior anti-tumor therapy (including surgery, radiation therapy, systemic therapy),and date of most recent disease progression.
- Record recent medication history, including vitamins, herbal preparations, blood products, and other over the counter (OTC) drugs.
- Record blood pressure (BP), heart rate (HR), respiratory rate (RR) and temperature measurements. In subjects with known significant pulmonary disease, measure oxygen saturation using pulse oximeter after a 2 minute walk

- Perform a complete physical examination, including height and weight.
- Perform tumor assessment with MRI of the brain per RANO criteria both preoperatively and postoperatively within 5 days of dosing respectively. (refer to Appendix C)
- Assess Eastern Cooperative Oncology Group (ECOG) Performance Status score (see Appendix B, Eastern Cooperative Oncology Group Performance Status Scale).
- Draw blood samples for hematology, chemistry and coagulation.
- Obtain a blood sample for serum HCG pregnancy test in female subjects of child-bearing potential (all female subjects unless surgically sterilized or at least 1 year post-menopausal).
- Obtain a urine sample for urinalysis with micro.
- Obtain a baseline ECG to assess for cardiac arrhythmias or evidence of recent cardiac events.
- Review inclusion and exclusion criteria (see Section 4, Study Population).

5.3 TREATMENT PERIOD- CYCLES 1-6

Study drug should be administered with 60 min of the pre-surgical timepoint and within ±2 days of the nominal time point for postoperative cycles. Lab tests used for determining dosing must be done within 5 days before the first dose of study drug (Cycle l/Day 1), within 3 days before Days 1 and 8 of all subsequent cycles. All other required study assessments should be obtained within 5 days of the nominal time point unless otherwise specified. Subjects must receive their single pre-surgical dose of study drug within 21 days of the start of screening. Postoperative dosing (Cycle 1/Day 1) may begin no sooner than 21 days postoperatively, and a maximum of 60 days postoperatively. Patients not able to begin treatment postoperatively within 60 days of surgery should be removed from the study, unless exception is given by the study PI and a representative from Immunomedics.

5.3.1 Presurgical Evaluations and Procedures

The following procedures will be done in all subjects**:**

- If screening assessments have not been performed within 3 days of planned surgery, then screening assessments will be repeated except for informed consent, demographics, MRI, medical/surgical history, and ECG;
- Serum and whole blood will be collected from patients on day -1 within 30 minutes at the end of Sacituzumab infusion, and intra-operatively at the time of tissue collection for biomarker analysis.

5.3.2 Procedures (Day 1 of each cycle)

Before administering Sacituzumab govitecan, the following procedures will be done in all subjects, at the post-surgical visit or within 5 days of Cycle 1 Day 1:

- Record interim medical history since screening;
- Confirm that subject continues to meet inclusion/exclusion criteria;
- Record concomitant medications for previous 14 days;
- Draw serum and whole blood samples for biomarker analysis

The following procedures will be done predose on all subjects within a 5-day window prior to Cycle 1 Day 1 and within 3 days of Day 1 for **All Future Cycles** (unless otherwise specified) and before administering Sacituzumab govitecan in subjects:

- Assess Eastern Cooperative Oncology Group (ECOG) Performance Status score;
- Assess whether subject is adequately hydrated for administration of study drugs
- Cycle 2 and all subsequent cycles: Record AEs since last visit;
- Record concomitant medications since last cycle;
- Record weight and vital signs;
- Detailed physical exam including neurologic assessment.
- Draw blood samples for hematology and chemistry;
- Draw serum and whole blood samples for biomarker analysis
- Obtain (serum or urine) pregnancy test (prior to start of Cycle 1, 3 and 5 only; females of childbearing potential)
- Administer Sacituzumab

5.3.3 Procedures (Day 8 of each cycle)

- A physical exam from Day 1 will be taken into consideration for D8 of cycle as well.
- Record AEs since the last visit, on the day of infusion D8
- Measure and record vital signs (BP, HR, RR, temperature) on D8
- Obtain blood samples for hematology, chemistry on Day8
- Administer Sacituzumab govitecan on D8 of 21-day treatment cycle.

5.4 STUDY TERMINATION/EARLY STUDY TERMINATION AND SURVIVAL FOLLOW-UP

5.4.1 Study Termination / Early Study Termination

This visit will occur 3-4 weeks after the start of Cycle 6 or at least 2 weeks after the last dose of Sacituzumab govitecan treatment for subjects who terminate early. Subjects who have not progressed after 6 cycles maybe permitted to continue therapy on a case-by-case basis after discussion with the PI. The following will be done at the Termination/Early Termination visit:

- Record concomitant medications, including vitamins, herbal preparations, blood products, and other OTC drugs since the last visit;
- Record AEs since the last visit;
- Perform a complete physical examination, including weight;
- Assess Eastern Cooperative Oncology Group (ECOG) Performance Status score;
- Measure and record vital signs (BP, HR, RR, temperature);
- Obtain blood samples for hematology and chemistry
- Obtain serum and whole blood samples for biomarker analysis
- Obtain a blood sample for serum HCG pregnancy test in female subjects of child-bearing potential (all female subjects unless surgically sterilized or at least 1 year post-menopausal);
- Perform tumor assessments, using the same imaging assessments done at baseline, if not done within past 4 weeks.

In accordance with good medical practice, any ongoing study drug-related AE present at study termination, including a clinically significant laboratory test abnormality, will be followed until resolved or until the event stabilizes and the overall clinical outcome has been ascertained. Adverse events starting up to 30 days after the last dose of study medication, or until the start of an alternate medication, may be collected by telephone contacts.

5.4.2 Survival Follow-up

When a subject has completed the study termination or early termination visit, he/she and/or a family member will be contacted for survival information every 3 months until one year from first dose. Anti-tumor therapy (description and dates) since the last contact will be collected at each survival follow up.

5.4.3 Termination Reasons

- Death.
- Withdrawal of consent for the study at any time for any reason.
- Lost to follow-up.
- Sponsor decision.

5.5.1 Safety Procedures

5.5.1.1 Physical Examination

A complete physical examination will be performed at screening and at study termination or early study termination and results will be recorded by the investigator (or designee). Limited physical examination will be done within 5 days before Day 1 of each cycle (q21days). Body weight will be measured on Day 1 of every cycle. The results of the physical examinations will be used for safety monitoring purposes only. At each study visit, according to good medical practice, the subject's general health (e.g., appearance, adequacy of hydration, presence of illness or injury, temperature, and vital signs indicative of a concurrent illness) will be assessed to determine whether continued dosing is appropriate.

5.5.1.2 Vital Signs

BP, HR, RR and temperature will be measured at the following time points:

- Screening
- All subjects: Day 1 and Day 8 of every cycle (predose and postdose for each study drug administered)
- Study Termination or Early Study Termination

Blood pressure and HR measurements should be obtained with the subject's arm unconstrained by clothing or other material. The measurements will be obtained with the appropriate cuff size from the opposite arm from that used for blood sampling, where possible, which is supported at the level of the heart. All BP measurements will be obtained from the same arm throughout the dosing period. The cuff should be placed on the designated arm at least 10 minutes prior to taking BP measurements.

5.5.1.3 Disease Assessment

Patients will be assessed at screening, within 3 days prior to the first post-surgical cycle, and at the end of every even cycle, with MRI per the modified RANO criteria.

6. MATERIALS AND SUPPLIES

6.1 DOSAGE, FORM, DRUG SUPPLY AND STORAGE

Sacituzumab govitecan is formulated for investigational use only. Formulation consists of 10 mg/mL sacituzumab govitecan formulated in 25 mM MES, pH 6.5, together with the following excipients (25 mM trehalose, 0.01% Polysorbate 80), which are then lyophilized. Glass vials containing 200 mg of sacituzumab govitecan as a sterile, non-pyrogenic, lyophilized powder are to be stored under refrigerated conditions (2-8ºC) until used.

Each vial is labeled "For Clinical Trial Use Only. Caution: New Drug-Limited by Federal (or United States) Law to Investigational Use. Sponsor: Immunomedics, Inc" and identified by study drug name, lot number, and dose. Since the formulated drug product contains no preservative, vials should be used only once. Sacituzumab govitecan will be provided in single vial packaging.

6.2 STUDY DRUG PREPARATION AND ADMINISTRATION

Reconstitute with normal saline and dilute immediately into infusion bags. Initiate the infusion within 1 hour of reconstitution/dilution. If infusion is delayed beyond 1 hour, refrigerate at 2-8°C for no more than 4 hours from reconstitution/dilution prior to infusion. If refrigerated, allow the diluted solution to come to room temperature prior to administration. If infusion does not begin within 4 hours after reconstitution/dilution, dispose of the original preparation and prepare a new infusion bag by reconstitution and dilution from new vials. Discard any unused portion in the vial. The product does not contain a preservative.

Appropriate use of aseptic technique should be employed in preparing the dose. Allow the sacituzumab govitecan vials to warm to room temperature to allow faster dissolution. The 200 mg of lyophilized powder in each vial should be reconstituted using 20 mL of 0.9% sterile sodium chloride (normal saline). The reconstituted solution should be gently shaken and allowed to dissolve for up to 15 minutes. Calculate the prescribed dose in mg based on the patient's bodyweight at the beginning of EACH cycle (or more frequently for > 10% change in body weight or if required by institutional policy; see Table 6.1 below).

The appropriate calculated amount should then be withdrawn from the supplied vials of study drug. The Sponsor recommends using a 21-gauge needle. Inject the solution into a glass or plastic infusion container slowly to minimize foaming and do not shake the contents. Adjust the volume in the infusion container as needed with normal saline to obtain a concentration of 1.1-3.4 mg/mL (total volume should not exceed 500 mL). Only normal sterile saline should be used since the stability of the reconstituted product has not been determined with other infusion- based solutions. The prepared study drug is stable for up to 8 hours at room temperature, however, as there is no preservative, the prepared study drug should be refrigerated.

Table 6.1 Preparation for 10 mg/kg Dosing (Assumes 70-kg Subject)			
**Total Dose Needed**		**70 kg x 10mg/kg**	**700 mg dose needed**
Step 1	Calculate number of vials Required	700 mg ÷ 200 mg/vial = 3.5	4 vials needed
Step 2	Reconstitute each vial by adding 20 mL 0.9% Sodium Chloride Injection, USP, to each vial	Each vial contains 200 mg/vial ÷ 20 mL Sodium Chloride Injection	10 mg/mL Reconstituted Product
Step 3	Calculate number of mL's needed to equal desired dose	700 mg dose ÷ 10 mg/mL	70 mL of reconstituted drug product needed to achieve desired dose
Step 4	Adjust volume in 250 mL infusion bag	250 mL 0.9% Sodium Chloride Injection, USP extract 70 mL	180 mL remaining in infusion bag
Step 5	Withdraw the appropriate calculated amount from the reconstituted vials of study drug and slowly inject into the infusion bag	70 mL of reconstituted drug product added to 180 mL 0.9% Sodium Chloride Injection, USP	250 mL Total Volume
Final Concentration		700 mg ÷ 250 mL	2.8 mg/mL

6.3 DRUG ACCOUNTABILITY

The investigator is responsible for the control of drugs under investigation. Adequate records of the receipt and disposition of all study drug shipped to the site must be maintained. Records will include dates, quantities received, quantities dispensed, and the identification codes of the subjects who received study drug. The individual administering the study drug will write the study number, subject number, date, and start/stop times of administration on the study drug label, and the Drug Accountability Record, as appropriate.

6.4 DISPOSITION OF USED AND UNUSED VIALS OF STUDY MEDICATION

All used, partially used, and unused vials must be retained by the pharmacist. Periodically throughout and at the conclusion of the study, inventory checks and accountability of study materials will be conducted by a representative of Immunomedics or its designated agent. Once accountability is completed, a Immunomedics representative will authorize the return to a designated facility or release for local destruction of all used, partially used, and unused vials. Partially used or empty vials may be destroyed prior to Immunomedics's review per the site's standard institutional procedures and local and federal regulations as applicable, and with appropriate documentation after two study staff members have reconciled and documented the count on an Accountability Record. The completed Drug Accountability and Drug Destruction Record(s) will be returned to Immunomedics.

The investigator's copy of the Drug Return Destruction Record(s) must accurately document the destruction of all study drug supplies. Records will also include dates, lot numbers, and quantities returned to Immunomedics or its designated agent or destroyed locally.

7 MANAGEMENT OF INTERCURRENT EVENTS

Comprehensive assessments of any apparent toxicity experienced by the subject will be performed throughout the course of the study. Study site personnel will report any clinical AE, whether observed by the investigator or reported by the subject.

7.1.1 Grading of Toxicity

Clinical AEs or abnormal laboratory test results will be assessed by the principal investigator or other designated other physician, in accordance with the CTCAE v5.0 criteria.

7.1.2 Monitoring and Treatment of Toxicity

A physician or other qualified medical professional (e.g. Physician Assistant, Nurse Practitioner) designated by the Principal Investigator will manage and treat any toxicity.

7.2 ADVERSE EVENTS

A physician or other qualified medical professional (e.g. Physician Assistant, Nurse Practitioner) designated by the Principal Investigator will assess the seriousness, severity, and causality of an AE based on the following definitions.

7.2.1 Defining Adverse Events

An adverse event (AE) is any undesirable event occurring to or in a subject enrolled in a clinical trial, whether or not the event is considered related to the study drug (Sacituzumab Govitecan). This includes the time periods beginning after the first administration of study drug until 30 days after the last dose of study drug or until a start of alternative therapy.

Adverse events include the following types of occurrences:

1) Suspected adverse reactions;
2) Other medical experiences, regardless of their relationship to the study drug, such as injury, causes for surgery, accidents, increased severity of pre-existing symptoms, apparently unrelated illnesses, and significant abnormalities in clinical laboratory values, physiological testing, or physical examination findings; and
3) Reactions from drug overdose, abuse, withdrawal, sensitivity, or toxicity.

7.2.1.1 Serious Adverse Events

A serious adverse event (SAE) is any adverse' experience that occurs at any dose and results in any of the following outcomes.

1) **Death.** This includes any death that occurs during the conduct of the clinical study, including deaths that appear to be completely unrelated to the study drug (e.g., car

accident). However, deaths that occur due to disease progression are not considered SAEs, but should be reported as a death on study. If a subject dies during the study, and an autopsy is performed, the autopsy results should be sent to Immunomedics. Possible evidence of organ toxicity and the potential relationship of the toxicity to the study rug are of particular interest. The autopsy report should distinguish between the relationship between the underlying diseases, their side effects, and the cause of death.

2) **Life-threatening adverse experience.** This includes any AE during which the subject is, in the view of the investigator, at immediate risk of death from the event as it occurs. This definition does not include any event that may have caused death if it had occurred in a more severe form.
3) Persistent or significant disability or incapacity
4) Inpatient hospitalization or prolongation of existing hospitalization
5) Congenital anomaly or birth defect
6) Other medically important event which, according to appropriate medical judgment, may require medical or surgical intervention to prevent one of the outcomes listed above.
7) Pregnancy occurring in subjects treated with Sacituzumab Govitecan should be reported using the serious adverse event reporting form.

7.2.1.2 Non-serious adverse events

A non-serious AE includes any AE that is not defined as an SAE.

7.2.1.3 Unexpected adverse events

An unexpected AE is any AE that is not identified in nature, severity or frequency.

7.2.2 Documenting All Adverse Events

Record all AEs as descriptive findings (symptoms, or laboratory, physical exam, or vitals abnormalities) or diagnoses if etiology is known. Included are all AEs that occur after the start of treatment or within 30 days of administration of the last dose of study drug. Record AEs of any severity and AEs that are assessed as serious or not serious.

Note: Unchanged, chronic conditions and cancer symptoms present at baseline are NOT AEs and should not be recorded unless there is an exacerbation or worsening in severity of a chronic condition or cancer symptom after the first administration of study drug until 30 days after the last dose of study drug. Chronic conditions and/or cancer symptoms that exacerbate or worsen in severity should be documented as a "worsening" condition. Death due to disease progression and measures of disease progression collected as efficacy endpoints (eg increasing tumor size or new lesions) are not considered adverse events, but should be collected as termination reasons (if applicable) and/or noted in tumor assessment appropriate. Other reasons for death occurring during the AE reporting period are SAEs and should be reported as such.

7.2.2.1 Grading of Adverse Events

Severity of AEs or clinically significant laboratory test results will be assessed in accordance with the grading scale presented in the Common Toxicity Criteria for Adverse Events (CTCAE) version 4.0. A copy of this document can be found at the following

internet site: http://cte.info.nih.gov/reporting/ctc.html. Clinically significant abnormal laboratory results and lab results requiring an intervention will be recorded as AEs and should describe whether the lab result was increased or decreased. The following definitions for rating severity of AEs will be used for events not covered in the CTCAE.

Grade 1: Mild; awareness of signs or symptoms that are easily tolerated, are of minor irritant type, cause no loss of time from usual activities, do not require medication or further medical evaluation, and/or are transient.

Grade 2: Moderate; signs or symptoms sufficient to interfere with function but not activities of daily living.

Grade 3: Severe; signs or symptoms sufficient to interfere with activities of daily living; signs and symptoms may be of a systemic nature, or require further medical evaluation and/or treatment.

Grade 4: Disabling or with life-threatening consequences. (This definition does not include any event that might have caused death if it had occurred in a more severe form.)

Grade 5: Death

7.2.2.2 Relationship to Study Drug

Using the following criteria, investigators will assess whether there is a reasonable possibility that the study drugs (Sacituzumab Govitecan) caused or contributed to the AE.

Yes - The time sequence between the onset of the AE and study drug administration is consistent with the event being related to study drug; and/or There is a possible biologic mechanism for study drug causing or contributing to the AE; and the AE may or may not be attributed to concurrent/underlying illness, other drugs, or procedures.

No - Another cause of the AE is most likely; and/or the time sequence between the onset of the AE and study drug administration is inconsistent with a causal relationship; and/or a causal relationship is considered biologically unlikely.

7.2.2.3 Abnormal Laboratory Test Results as Adverse Events

The investigator will monitor the laboratory test results and determine the clinical significance of any result that falls outside of the reference range. In accordance with good medical practice, any clinically significant abnormal laboratory test results must be followed until resolved or stabilized. Abnormal laboratory test results should not be reported as AEs unless, in the opinion of the investigator, the results constitute or are associated with a clinically relevant condition or require intervention.

In the event of unexplained, clinically significant abnormal laboratory test results, the tests should be repeated immediately and followed up until the values have returned to within the reference range or to baseline for that subject.

7.2.3 Reporting and Documenting Serious Adverse Events

Serious adverse events (SAE) that occur at any time point after the first dose of study drug until 30 days after the last dose of study drug must be reported. SAEs must be reported as

per institutional policy and as required under the Data Safety Monitoring Plan (see 10.6 for DSMP).

1) Submit all known subject information (listed below) within 24 hours of knowledge of the SAE occurrence. The following information should also be entered in the database (or as much as possible to obtain and still report the event within 24 hours):
    a) Subject's Demographic Data
    b) Subject's weight
    c) Description of SAE, including date of onset and duration, severity, and outcome
    d) All dosing data of study drugs administered up to the date the SAE occurred
    e) Action taken regarding study drug administration
    f) Relationship of SAE to study drugs
    g) Concomitant medications, including regimen and indication
    h) Intervention, including concomitant medications used to treat SAE
    i) Pertinent laboratory data and diagnostic tests conducted and date
    j) Pertinent medical history of subject
    k) Date of hospital admission discharge (if applicable)
    l) Date of death (if applicable)
2) Perform appropriate diagnostic tests and therapeutic measures, and submit all follow-up substantiating data, such as diagnostic test reports and autopsy report to Immunomedics.
3) Conduct appropriate consultation and follow-up evaluations until the events are resolved, stabilized, or otherwise explained by the principal investigator.
4) Review each SAE report and evaluate the relationship of the SAE to study treatment and to the underlying disease. Immunomedics will determine whether the SAE is unexpected in nature.
5) Based on a cooperative assessment of the SAE with Immunomedics, a decision for any further action will be made. The primary consideration is subject safety. If the discovery of a new SAE related to the study drug raises concern over the safety of its continued administration to subjects, Immunomedics will take immediate steps to notify the FDA.
6) The investigator must report all SAEs and unexpected problems promptly to his or her IRB/IEC, as appropriate (see ICH Guidelines, Good Clinical Practice [E6]).

Other actions regarding SAEs might include the following:
    a) Protocol amendment
    b) Discontinuation or suspension of the protocol
    c) Modification of informed consent to include recent findings
    d) Informing current study participants of new findings
    e) Identification of specific AEs as drug-related

7.2.4 Follow Up of Adverse Events

All AEs are followed until they are resolved or determined to be irreversible or otherwise explained by the principal investigator.

7.3 CONCOMITANT AND EXCLUDED THERAPY

UGT1A1 (Uridine diphosphate glucuronosyl transferase 1A1) inhibitors or inducers are to be avoided during study participation due to potential affect on SG metabolism.
All medications and blood products (prescription and over-the-counter including herbal preparations) taken within 21 days of Cycle l/Week 1 will be recorded by the investigator (or designee). The reason(s) for treatment, dosage, and dates of treatment should be recorded in the source documents. In addition, concomitant medications used to treat adverse events occurring up to 30 days after the last dose of study drug will be recorded.

Female subjects who have been on hormone replacement therapy (HRT) for menopausal symptoms for a period of at least 2 months will not be excluded from the study provided the HRT regimen remains unchanged during the conduct of the study.

8. REMOVING SUBJECTS FROM THE STUDY

8.1 CRITERIA FOR TERMINATION

Subjects are free to discontinue (withdraw) at any time during this clinical trial. If a subject withdraws from participation in the study during the treatment period, he or she should be encouraged to return for an early termination visit for evaluation of safety (see Section 5.4.1, Study Termination/Early Study Termination).

The investigator has the right to discontinue any subject from study drug administration or study participation. Reasons for subject discontinuation may include, but are not limited to, the following:

- Clinically significant deterioration of the subject's condition;
- Disease progression;
- Requirement for other anti-tumor therapy during the study;
- Noncompliance;
- Pregnancy;
- Significant AE;
- Subject's right to withdraw from the study at any time, with or without stated reason;
- Significant protocol violation;
- Lost to follow-up;
- Death;

Any other reason that, in the opinion of the principal investigator, would justify the removal of a subject from the study. The primary consideration in any determination to discontinue a subject's participation must be the health and welfare of the subject.

All subjects will be instructed on the importance of complying with the requirements of the study. It is expected that subjects will complete all of the necessary visits. If a subject does not return for follow-up visits as directed or does not adhere to the study requirements, the investigator will determine if early withdrawal should occur.

8.2 DOCUMENTATION

The primary reason for early removal of a subject from the study must be documented clearly, and must be completed for any subject who has received any amount of drug during the treatment period. If the reason for early withdrawal is an AE or an abnormal laboratory value, the specific event or test result must also be recorded.

8.3 PROCEDURES FOR SUBJECTS WHO WITHDRAW EARLY

Following early termination, the subject should be informed about which evaluations are necessary to monitor his or her safety. In addition, subjects should be encouraged to complete any procedures or evaluations outlined in Section 5.4, Study Termination/Early Study Termination.

8.4 REPLACEMENT OF SUBJECTS

Subjects who have not received Sacituzumab Govitecan will be replaced. Subject enrollment numbers are unique and will not be re-assigned.

9. CONDITIONS FOR INITIATING, MODIFYING, OR TERMINATING THE STUDY

9.1 INSTITUTION REVIEW

The investigator will submit this protocol, any protocol modifications, and the subject consent form to be used in this study to the local Institutional Review Board (IRB) for review and approval. A letter confirming IRB approval of the protocol and subject consent form, and an IRB approved informed consent form must be forwarded to Immunomedics prior to the enrollment of subjects into the study.

9.2 INFORMED CONSENT

The investigator or his or her designee must explain to the subject, in the presence of a witness, the purpose and nature of the study, the study procedures, and the possible adverse effects, and all other elements of consent as defined in 21 CFR Part 50 and Clinical Trial Directive or ICH E6 guidelines before enrolling that subject in the study. It is the investigator's (or designee's) responsibility to obtain informed written consent from each subject, or if appropriate, the subject's parent or legal guardian.

9.3 MODIFICATIONS

If any modifications in the experimental design, dosages, parameters, subject selection, or any other sections of the protocol are indicated or required, the investigator will consult with Immunomedics before such changes are instituted. Modifications will be accomplished through formal amendments and approval from the appropriate IRB.

9.4 DEVIATIONS

The PI will consider any deviations from the protocol on a case-by-case basis. The investigator or other designated alternate in his absence will contact the local DSMB (DSMB2) as soon as possible to discuss the associated circumstances. The principal investigator and the DSMB will then decide whether the subject should continue to participate in the study. All protocol deviations and the reasons for such deviations must

be noted in the source documents and will be reported to the CTRC DSMC and to the IRB as per standard institutional policy.

9.5 TERMINATION

Although there are no predefined criteria for termination, if Immunomedics or the investigators) discover conditions during the course of the study that indicate it should be discontinued, an appropriate procedure for terminating the study will be instituted, including notification of the appropriate regulatory agencies and IRB.

10. INVESTIGATOR'S RESPONSIBILITIES

10.1 RESPONSILITIES/PERFORMANCE

The investigator will ensure that this study is conducted in accordance with all regulations governing the protection of human subjects. The investigator will adhere to the basic principles of "Good Clinical Practice," as outlined in Title 21 of the Code of Federal Regulations (CFR), Part 312, Subpart D, "Responsibilities of Sponsors and Investigators"; 2 1 CFR, Part 50, "Protection of Human Subjects"; 21 CFR, Part 56, "Institutional Review Boards"; and the US Food and Drug Administration (FDA) guideline entitled "Good Clinical Practice: Consolidated Guideline". For studies conducted outside of the USA, the investigator will ensure adherence to the principles outlined in the International Conference on Harmonization (ICH) E6 "Guideline for Good Clinical Practice". Additionally, this study will be conducted in compliance with the Declaration of Helsinki and with any local laws and regulations of the country in which the research is conducted. The investigator will ensure that all work and services described in or associated with this protocol will be conducted in accordance with the investigational plan, applicable regulations, and the highest standards of medical and clinical research practice. The investigator is responsible for the control of drugs under investigation. The investigator will provide copies of the study protocol and Investigator's Brochure to all sub- investigators, pharmacists, and other staff responsible for study conduct.

10.2 CONFIDENTIALITY

The investigator must ensure that each subject's anonymity will be maintained and each subject's identity will be protected from unauthorized parties. A number will be assigned to each subject upon study entry and the number and the subject's initials will be used to identify the subject for the duration of the study. Documents submitted to Immunomedics should not identify a subject by name. Documents that are not submitted to Immunomedics (eg, signed consent form) will be maintained by the investigator in strict confidence.

10.3 INSTITUTIONAL REVIEW

The investigator will submit this protocol, any protocol modifications, and any accompanying material provided to the subject (eg, informed consent form, subject information sheets, or descriptions of the study used to obtain informed consent) to the appropriate IRB for review and approval. A letter confirming IRB approval of the protocol and subject consent forms, and an IRB approved informed consent form must be forwarded to Immunomedics prior to the enrollment of subjects into the study. A copy of the approved subject consent form will also be forwarded to Immunomedics.

10.3.1 Modifications

If any modifications in the experimental design, dosages, parameters, or subject selection are indicated or required, the investigator will consult with Immunomedics (or vice versa) before such changes are instituted. Modifications will be accomplished through formal amendments to this protocol and approval by the appropriate IRB. Copies of all subsequent IRB approvals (e.g., protocol amendments) must be sent to Immunomedics.

10.3.2 Protocol Deviations

The PI will consider any deviations from the protocol on a case-by-case basis. The investigator or other designated alternate in his absence will contact the local DSMB (DSMB2) as soon as possible to discuss the associated circumstances. The principal investigator and the DSMB will then decide whether the subject should continue to participate in the study. All protocol deviations and the reasons for such deviations must be noted in the source documents and will be reported to the Mays Cancer Center at UT Health San Antonio and the IRB as per standard institutional policy.

10.3.3 Termination

If Immunomedics and/or the investigator(s) discover conditions during the course of the study that indicate it should be discontinued, an appropriate procedure for terminating the study will be instituted, including notification of the appropriate regulatory agencies and the IRB.

10.4 INFORMED CONSENT AND PERMISSION TO USE PROTECTED HEALTH INFORMATION

It is the responsibility of the investigator to obtain written informed consent from each subject participating in this study after adequate explanation, in lay language, of the methods, objectives, anticipated benefits, and potential hazards of the study. The investigator must also explain that the subject is completely free to refuse to enter the study or to discontinue participation at any time (for any reason) and receive alternative conventional therapy as indicated. Prior to study participation, each subject will sign an IRB approved informed consent form, which will be in form and substance acceptable to Immunomedics, and receive a copy of same (and information leaflet, if appropriate). For subjects not qualified or able to give legal consent, consent must be obtained from a parent, legal guardian, or custodian.

The investigator or designee must explain to the subject before enrollment into the study that for evaluation of study results, the subject's protected health information obtained during the study may be shared with Immunomedics, regulatory agencies, and IECs/IRBs. It is the investigator's (or designee's) responsibility to obtain permission to use protected health information per HIPAA from each subject, or if appropriate, the subjects' parent or legal guardian.

10.5 SOURCE DOCUMENTATION AND INVESTIGATOR FILES

The investigator must maintain adequate and accurate records to fully document the conduct of the study and to ensure that study data can be subsequently verified. These

documents should be classified into 2 separate categories: (1) investigator study file and (2) subject clinical source documents that corroborate data collected.

Subject clinical source documents would include hospital clinic patient records; physician's and nurse's notes; appointment book; original laboratory, ECG, EEG, radiology, pathology, and special assessment reports; pharmacy dispensing records; subject diaries; signed informed consent forms; consultant letters; and subject screening and enrollment logs.

The following will be documented in source documents at the site:

1) Medical history/physical condition and diagnosis of the subject before involvement in the study sufficient to verify protocol entry criteria (if not already present);
2) Study number, assigned subject number, and verification that written informed consent was obtained (each recorded in dated and signed notes on the day of entry into the study);
3) Progress notes for each subject visit (each dated and signed);
4) Study drug dispensing and return;
5) Review of laboratory test results;
6) Adverse events (action taken and resolution);
7) Concomitant medications (including start and stop dates); and
8) Condition of subject upon completion of or early termination from the study.

10.5.1 Exclusion Log

The investigator must keep a record listing all patients considered for entry into the study but subsequently excluded. The reason for each exclusion will be recorded in the Subject Exclusion Log.

10.6 DATA SAFETY MONTIORING PLAN

A Data and Safety Monitoring Plan is required for all an individual protocols conducted at Mays Cancer Center at UT Health San Antonio. All protocols conducted at Mays Cancer Center are covered under the auspices of the Mays Cancer Center Institutional Data Safety Monitoring Plan (DSMP).

The Mays Cancer Center Institutional DSMP global policies provide individual trials with:

- institutional policies and procedures for institutional data safety and monitoring,
- an institutional guide to follow,
- monitoring of protocol accrual by the Mays Cancer Center Protocol Review Committee,
- review of study forms and orders by the Forms Committee,
- independent monitoring and source data verification by the Mays Cancer Center QA Monitor/Auditor
- tools for monitoring safety events,
- monitoring of UPIRSO’s by the Director of Quality Assurance and DSMC,
- determining level of risk (Priority of Audit Level Score – PALS) ,
- oversight by the Data Safety Monitoring Committee (DSMC), and
- verification of protocol adherence via annual audit for all Investigator Initiated Studies by the Mays Cancer Center Quality Assurance Division.

10.6.1 Monitoring Safety

Due to the risks associated with participation in this protocol, the Mays Cancer Center DSMB2 in conjunction with the Principal Investigator will perform assessment of adverse events, adverse event trends and treatment effects on this study. The Mays Cancer Center DSMB2 acts as an independent Data Safety Monitoring Board (DSMB) for IIS conducted at Mays Cancer Center. The Mays Cancer Center will monitor data throughout the duration of a study to determine if continuation of the study is appropriate scientifically and ethically. An additional layer of review is provided by the Mays Cancer Center Data Safety Monitoring Committee (DSMC) who will review the DSMB's quarterly reports.

Baseline events and adverse events will be captured using the Mays Cancer Center Master Adverse Events Document for each patient using CTCAE V4.0 for the grading and attribution of adverse events. Usage of the CTRC Master Adverse Events Document centrally documents:

- the event and grades the seriousness of the event,
- if the event was a change from baseline,
- the determination of the relationship between the event and study intervention,
- if the event was part of the normal disease process, and
- what actions were taken as a result of the event.

10.6.2 Reporting Requirements

For this study, the Master Adverse Events Documents collected on patients for this protocol will be reviewed by the Principal Investigator on a monthly basis to determine if a serious safety problem has emerged that result in a change or early termination of a protocol such as:

- dose modification,
- suspending enrollment due to safety or efficacy, or
- termination of the study due to a significant change in risks or benefits.

The PI will provide the DSMB2 with the monthly findings for discussion and review during their meetings. Specific areas of concern that will be reported to the DSMB2 regarding this study that would qualify as an endpoint are:

- evidence from surgically resected tissue that Sacituzumab Govitecan does in fact NOT penetrate the blood brain barrier and as such would have no therapeutic benefit to patients on study
- unacceptable toxicity that would prevent patients from receiving treatment and therefore benefit

As per the Mays Cancer Center DSMP, any protocol modifications, problematic safety reports, unanticipated problems, and suspension or early termination of a trial must be reported to all members of the research team. Suspension and early termination of a trial must also be reported immediately to the Director of Quality Assurance who will promptly notify the sponsor and the UTHSA IRB.

The PI will review the Master Adverse Events documents to determine the significance of the reported events and will provide findings using the Investigator Initiated Study Quarterly DSMC Report Form on a monthly basis with the DSMB2. The DSMB2 will review the information provided by the PI and report to the Mays Cancer Center DSMC on a quarterly basis unless an emergent issue has been identified. The Investigator Initiated Study Quarterly DSMC Report Form includes information on adverse events, current dose levels, number of patients enrolled, significant toxicities per the protocol, patient status (morbidity and mortality) dose adjustments with observed response, and any interim findings. Any trend consisting of three or more of the same event will be reported to the Mays Cancer Center DSMC for independent review outside of the quarterly reporting cycle, which begins three months following protocol start up. The DSMB2 will also provide its findings to the Mays Cancer Center's Regulatory Affairs Division so that it may be provided to the UTHSA IRB with the protocol's annual progress report. Conflict of interest is avoided by the independent reviews of the Mays Cancer Center DSMB2, Mays Cancer Center DSMC and by ongoing independent review of UPIRSO's by the Director of Quality Assurance.

All SAE and UPRISO's will be reported following CTRC and UTHSCSA institutional guidelines.

**UTHSCSA SAE/UPRISO REPORTING REQUIREMENTS**		
Type Event	Report to	Timeframe
All AE, SAE and UPIRSO	Regulatory Affairs and DQA	Same as other notification timeframes except for SAE/AE which should be reported on Monday for the prior week
SAE	Clinical Trial Sponsor	within 24 hours
AE/SAE	UTHSA IRB	Annually
UPIRSO - all	Clinical Trial Sponsor	within 24 hours of the PI determining a UPIRSO exists
UPIRSO - life threatening	UTHSA IRB	within 48 hours of the PI determining a UPIRSO exists
UPIRSO - non-life threatening	UTHSA IRB	within 7 days of the PI determining a UPIRSO exists

Expedited reporting may not be appropriate for specific expected adverse events for certain later Phase II and Phase III protocols. In those situations the adverse events that will not have expedited reporting must be specified in the text of the approved protocol. An expected Grade 3 event that is definitely related to the investigational agent is only to be reported if the patient is hospitalized using the generic reporting criteria. For instance, in a trial of an investigational agent where Grade 3 diarrhea requiring hospitalization is expected, only diarrhea requiring ICU care (Grade 4) might be designated for expedited reporting.

Serious adverse events on NCI sponsored trials utilizing a commercially available agent (with no IND's involved) will additionally be reported via the FDA's Medwatch program.

10.6.2 Assuring Compliance with Protocol and Data Accuracy

As with all studies conducted at Mays Cancer Center at UT Health San Antonio, the PI has ultimate responsibility for ensuring protocol compliance and data accuracy/integrity. Protocol compliance, data accuracy and reporting of events is further ensured by an annual audit conducted by the Data Safety Officer, whose audit report is shared with the PI, the research team and will be reviewed by the CTRC DSMC.

Mays Cancer Center DSMB Membership

The Mays Cancer Center has two DSMB's with a primary set of members specific to the histology of the study consisting of UTHSA faculty and staff. This Protocol will utilize DSMB#1 for Hematological studies or DSMB#2 for Solid Tumor Studies.

As per NCI guidelines and to eliminate conflict of interest (financial, intellectual, professional, or regulatory in nature), the Mays Cancer Center DSMB specific to this study will not treat patients on this protocol. Usage of the DSMB specific to the histology has been created to ensure that experts in that histology are represented on the DSMB assembled for this protocol, but may be expanded, at the PI's discretion, to include other members which may include:

- Experts in the fields of medicine and science that are applicable to the study (if not currently represented on the DSMB),
- Statistical experts,
- Lay representatives,
- Multidisciplinary representation, from relevant specialties including experts such as bioethicists, biostatisticians and basic scientists, and
- Others who can offer an unbiased assessment of the study progress.

Additional or alternate membership of in the DSMB is selected by the DSMC chair, in conjunction with the PI of this protocol.

Mays Cancer Center DSMB Charter and Responsibilities

The Mays Cancer Center DSMB will provide information on the membership composition, including qualifications and experience to both the UTHSA IRB and Mays Cancer Center PRC for review. The Mays Cancer Center DSMB for this study will act as an independent advisory board to the PI and will report its findings and recommendations to the PI, the UTHSA F and the Mays Cancer Center DSMC. Mays Cancer Center DSMB reports will utilize the Investigator Initiated Study Quarterly DSMC Report Form and meetings will occur on a monthly basis to review any updates from the prior meeting.

Once the protocol is activated, if not already established elsewhere in the protocol the Mays Cancer Center DSMB will establish and provide:

- procedures for maintaining confidentiality;
- statistical procedures including monitoring guidelines, which will be used to monitor the identified primary, secondary, and safety outcome variables;
- consider factors external to the study when relevant information becomes available, such as scientific or therapeutic developments that may have an impact on the safety of the participants or the ethics of the study;

- plans for changing frequency of interim analysis as well as procedures for recommending protocol changes;
- recommendation of dose escalation, MTD recommendation of early termination based on efficacy results;
- recommendation of termination due to unfavorable benefit-to-risk or inability to answer study questions;
- recommendation of continuation of ongoing studies;
- recommend modification of sample sizes based on ongoing assessment of event rates; and
- review of final results and publications.

11. REFERENCES

1. Shvartsur, A. and B. Bonavida, *Trop2 and its overexpression in cancers: regulation and clinical/therapeutic implications.* Genes & cancer, 2015. **6**(3-4): p. 84-105.
2. Trerotola, M., et al., *Upregulation of Trop-2 quantitatively stimulates human cancer growth.* Oncogene, 2012. **32**: p. 222.
3. Cubas, R., et al., *Trop2 expression contributes to tumor pathogenesis by activating the ERK MAPK pathway.* Mol Cancer, 2010. **9**: p. 253.
4. Shih, L.B., et al., *In vitro and in vivo reactivity of an internalizing antibody, RS7, with human breast cancer.* Cancer Res, 1995. **55**(23 Suppl): p. 5857s-5863s.
5. Stein, R., et al., *Advantage of yttrium-90-labeled over iodine-131-labeled monoclonal antibodies in the treatment of a human lung carcinoma xenograft.* Cancer, 1997. **80**(12 Suppl): p. 2636-41.
6. Varughese, J., et al., *High-grade, chemotherapy-resistant primary ovarian carcinoma cell lines overexpress human trophoblast cell-surface marker (Trop-2) and are highly sensitive to immunotherapy with hRS7, a humanized monoclonal anti-Trop-2 antibody.* Gynecol Oncol, 2011. **122**(1): p. 171-7.
7. Cardillo, T.M., et al., *Humanized Anti-Trop-2 IgG-SN-38 Conjugate for Effective Treatment of Diverse Epithelial Cancers: Preclinical Studies in Human Cancer Xenograft Models and Monkeys.* Clinical Cancer Research, 2011. **17**(10): p. 3157-3169.
8. Bardia, A., et al., *Efficacy and Safety of Anti-Trop-2 Antibody Drug Conjugate Sacituzumab Govitecan (IMMU-132) in Heavily Pretreated Patients With Metastatic Triple-Negative Breast Cancer.* Journal of Clinical Oncology, 2017. **35**(19): p. 2141-2148.
9. Bardia, A., et al., *Efficacy of sacituzumab govitecan (anti-Trop-2-SN-38 antibody-drug conjugate) for treatment-refractory hormone-receptor positive (HR+)/HER2- metastatic breast cancer (mBC).* Journal of Clinical Oncology, 2018. **36**(15_suppl): p. 1004-1004.
10. Lin, N.U., et al., *Sites of distant recurrence and clinical outcomes in patients with metastatic triple-negative breast cancer: high incidence of central nervous system metastases.* Cancer, 2008. **113**(10): p. 2638-45.
11. Jin, J., et al., *Incidence, pattern and prognosis of brain metastases in patients with metastatic triple negative breast cancer.* BMC Cancer, 2018. **18**(1): p. 446.
12. Niwinska, A., M. Murawska, and K. Pogoda, *Breast cancer subtypes and response to systemic treatment after whole-brain radiotherapy in patients with brain metastases.* Cancer, 2010. **116**(18): p. 4238-47.
13. Kruchko, C., et al., *CBTRUS Statistical Report: Primary Brain and Other Central Nervous System Tumors Diagnosed in the United States in 2011–2015.* Neuro-Oncology, 2018. **20**(suppl_4): p. iv1-iv86.
14. Stupp, R., et al., *Maintenance Therapy With Tumor-Treating Fields Plus Temozolomide vs Temozolomide Alone for Glioblastoma: A Randomized Clinical Trial.* JAMA, 2015. **314**(23): p. 2535-43.
15. Friedman, H.S., et al., *Bevacizumab alone and in combination with irinotecan in recurrent glioblastoma.* J Clin Oncol, 2009. **27**(28): p. 4733-40.
16. Ning, S., et al., *TROP2 expression and its correlation with tumor proliferation and angiogenesis in human gliomas.* Neurol Sci, 2013. **34**(10): p. 1745-50.
17. Harvard, B.I.o.M.a., *Broad Institute TCGA Genome Data Analysis Center (2016): Correlation between mRNA expression and clinical features.*

18. Verma, S., et al., *Trastuzumab emtansine for HER2-positive advanced breast cancer.* N Engl J Med, 2012. **367**(19): p. 1783-91.
19. Krop, I.E., et al., *Trastuzumab emtansine (T-DM1) versus lapatinib plus capecitabine in patients with HER2-positive metastatic breast cancer and central nervous system metastases: a retrospective, exploratory analysis in EMILIA.* Ann Oncol, 2015. **26**(1): p. 113-9.
20. Fabi, A., et al., *T-DM1 and brain metastases: Clinical outcome in HER2-positive metastatic breast cancer.* Breast, 2018. **41**: p. 137-143.
21. Jacot, W., et al., *Efficacy and safety of trastuzumab emtansine (T-DM1) in patients with HER2-positive breast cancer with brain metastases.* Breast Cancer Res Treat, 2016. **157**(2): p. 307-318.
22. Bartsch, R., et al., *Activity of T-DM1 in Her2-positive breast cancer brain metastases.* Clin Exp Metastasis, 2015. **32**(7): p. 729-37.
23. Scott, A.M., et al., *A phase I clinical trial with monoclonal antibody ch806 targeting transitional state and mutant epidermal growth factor receptors.* Proc Natl Acad Sci U S A, 2007. **104**(10): p. 4071-6.
24. Zalutsky, M.R., et al., *Pharmacokinetics and Tumor Localization of ¹³¹I-Labeled Anti-Tenascin Monoclonal Antibody 81C6 in Patients with Gliomas and Other Intracranial Malignancies.* Cancer Research, 1989. **49**(10): p. 2807-2813.
25. Cardillo, T.M., et al., *Sacituzumab Govitecan (IMMU-132), an Anti-Trop-2/SN-38 Antibody–Drug Conjugate: Characterization and Efficacy in Pancreatic, Gastric, and Other Cancers.* Bioconjugate Chemistry, 2015. **26**(5): p. 919-931.
26. Vredenburgh, J.J., et al., *Experience with irinotecan for the treatment of malignant glioma.* Neuro-oncology, 2009. **11**(1): p. 80-91.
27. *Genomics of Drug Sensitivity in Cancer Project* Available from: https://www.cancerrxgene.org/translation/Drug/1494.
28. Sharkey, R.M., et al., *Enhanced Delivery of SN-38 to Human Tumor Xenografts with an Anti-Trop-2–SN-38 Antibody Conjugate (Sacituzumab Govitecan).* 2015. **21**(22): p. 5131-5138.
29. Starodub, A.N., et al., *First-in-Human Trial of a Novel Anti-Trop-2 Antibody-SN-38 Conjugate, Sacituzumab Govitecan, for the Treatment of Diverse Metastatic Solid Tumors.* Clin Cancer Res, 2015. **21**(17): p. 3870-8.
30. Ocean, A.J., et al., *Sacituzumab govitecan (IMMU-132), an anti-Trop-2-SN-38 antibody-drug conjugate for the treatment of diverse epithelial cancers: Safety and pharmacokinetics.* Cancer, 2017. **123**(19): p. 3843-3854.
31. Bardia, A., et al., *Sacituzumab Govitecan-hziy in Refractory Metastatic Triple-Negative Breast Cancer.* N Engl J Med, 2019. **380**(8): p. 741-751.
32. Stupp, R., et al., *Radiotherapy plus concomitant and adjuvant temozolomide for glioblastoma.* N Engl J Med, 2005. **352**(10): p. 987-96.
33. Goli, K.J., et al., *Phase II trial of bevacizumab and irinotecan in the treatment of malignant gliomas.* 2007. **25**(18_suppl): p. 2003-2003.
34. Siegel, R.L., K.D. Miller, and A. Jemal, *Cancer Statistics, 2017.* CA Cancer J Clin, 2017. **67**(1): p. 7-30.
35. Martin, A.M., et al., *Brain Metastases in Newly Diagnosed Breast Cancer: A Population-Based Study.* JAMA Oncol, 2017. **3**(8): p. 1069-1077.
36. Niwinska, A., M. Murawska, and K. Pogoda, *Breast cancer brain metastases: differences*

in survival depending on biological subtype, RPA RTOG prognostic class and systemic treatment after whole-brain radiotherapy (WBRT). Ann Oncol, 2010. **21**(5): p. 942-8.
37. Anders, C., et al., *TBCRC 018: phase II study of iniparib in combination with irinotecan to treat progressive triple negative breast cancer brain metastases.* Breast Cancer Res Treat, 2014. **146**(3): p. 557-66.
38. Heist, R.S., et al., *Therapy of Advanced Non-Small-Cell Lung Cancer With an SN-38-Anti-Trop-2 Drug Conjugate, Sacituzumab Govitecan.* J Clin Oncol, 2017. **35**(24): p. 2790-2797.

Appendix A: Schedule of Assessments

Study Procedures	Screening (w/n 21 days of Surg)	Pre-Surg (Surg D-1)	Surgery (Surg D1)	Post-Surg Visit	Post-Surgical C1 (PSC1)		Post-Surgical C2 (PSC2)		Post-Surgical C3 (PSC3)		Post-Surgical C4 (PSC4)		Post-Surgical C5 (PCS5)		Post-Surgical C6 (PSC6) and future cycles		EOT*
Day					Day 1	Day 8	Day 1	Day 8	Day 1	Day 8	Day 1	Day 8	Day 1	Day 8	Day 1	Da y 8	
Informed Consent	X																
Demographics	X																
Med/Surg History	X																
Physical Exam (to include Adverse events and con meds)[C,F]	X	X		X	X	X	X		X		X		X		X		X
Vitals Signs	X	X		X	X	X	X	X	X	X	X	X	X	X	X	X	X
Height	X	X															
Weight	X	X		X	X	X	X	X	X	X	X	X	X	X	X	X	X
ECOG	X	X		X	X	X	X		X		X		X		X		X
ECG	X																
Hematology labs[A]	X	X			X	X	X	X	X	X	X	X	X	X	X	X	X
Chemistry labs[A]	X	X			X	X	X	X	X	X	X	X	X	X	X	X	X
Coagulation labs[A, B]	X	X			X												
Urinalysis[A, D]	X				X												
Pregnancy test	X				X				X				X				X
Sacituzumab 10mg/kg IV		X			X	X	X	X	X	X	X	X	X	X	X	X	
Neulasta (onPro)		X				X		X		X		X		X		X	
Tumor Assessment[E]	X			X							X						X
Serum Sampling		X	X		X		X		X		X		X		X		X
Tumor Sampling			X														
CSF Sampling / LP [G]			X			X											

A – Blood draws must be within 5 days of C1D1, and within 3 days of D1 and within 1 day of D8.
B – INR only if patient is on warfarin
C – Physical exam can be done within 5 days before Day 1 of Cycles 1, 3, and 5.
D – Screen: UA with micro. Other visits, urine dipstick for protein & glucose. If positive or change from BL, complete urinalysis w/micro.
E – Tumor Assessments at screening, post-surgical visit, and after every third cycle. Scanning to be done within 3 days of dosing at specified time points.
F – Physical exams due on Day 1 and D18 for presurgical and first postsurgery cycles then only on D1 unless clinically indicated.
* - Occurs 3-4 weeks after the start of C6 or at least 2 weeks after the last dose of Sacituzumab for subjects who terminate early. Survival follow-up will be done by telephone after EOT every 3 months until one year from first dose.
G – Lumbar puncture is an optional procedure and will only be done with subject consent if CSF was not collected during surgery.

APPENDIX B: EASTERN COOPERATIVE ONCOLOGY GROUP (ECOG) PERFORMANCE SCORE SCALE

Grade	Description
0	Fully active, able to carry on all pre-disease performance without restriction
1	Restricted in physically strenuous activity but ambulatory and able to carry out work of a light or sedentary nature, e.g., light house work, office work
2	Ambulatory and capable of all self-care but unable to carry out any work activities. Up and about more than 50% of waking hours
3	Capable of only limited self-care, confined to bed or chair more than 50% of waking hours
4	Completely disabled. Cannot carry on any self-care. Totally confined to bed or chair
5	Dead

APPENDIX C: RANO TUMOR RESPONSE

Table: Summary of the RANO Response Criteria

	CR	PR	SD	PD#
**T1-Gd +**	None	≥50% decrease	<50% decrease-<25% increase	≥25% increase*
**T2/FLAIR**	Stable or decrease	Stable or decrease	Stable or decrease	Increase*
**New Lesion**	None	None	None	Present*
**Corticosteroids**	None	Stable or decrease	Stable or decrease	**NA**
**Clinical Status**	Stable or increase	Stable or increase	Stable or increase	Decrease*
**Requirement for Response**	All	All	All	Any*

CR=complete response; PR=partial response; SD=stable disease; PD=progressive disease

Progression occurs when any of the criteria with * is present

NA: Increase in corticosteroids alone will not be taken into account in determining progression in the absence of persistent clinical deterioration

APPENDIX D:

Immunomedics "Product Inspection Form"

**Site ID:**		**Facility Name**		**Date Shipment Received and Inspected:**	
**Street Address:**		**City:**		**ZIP:**	
**Lot Number:**		**Expiration:**		**# of Vials Inspected:**	
**Temperature Upon Receipt:**					

Any Items that are marked "YES" requires a comment to be denoted

ITEM		STATUS (Circle)	COMMENTS
1	Is particulate matter present (e.g. foreign mater, hair, glass, fiber, metal)<br>**Note**: The container should be free of particulates	**YES / NO**	
2	Is there any discoloration of the reconstituted drug product?<br>**Note**: The drug product should be clear and pale yellow	**YES / NO**	
3	Is the vial broken (cracked neck, cracked body)?	**YES / NO**	
4	Is there any unsatisfactory sealing of the cap including any indication of tampering or evidence that could indicate abnormal conditions of material, container or cap under normal lighting conditions?	**YES / NO**	
5	Is the stopper damaged (stained, cracked, dented)?	**YES / NO**	
6	Is there visible cloudiness in the reconstituted drug product solution?	**YES / NO**	
7	Is the vial empty?	**YES / NO**	

Visual Inspection Performed By: ________________________________ **Date:** ________________

NOTE: For items denoted "YES", the subject Vial/Lot should be quarantined, and notify your CRA to contact the Immunomedics Quality Department should be contacted within 1 business day of the finding.

Immunomedics, Inc. Visual Inspection Form for IMMU-132 version date/number: 12-Feb-2019 ver 3.0
