# Supplementary material for: Sacituzumab Govitecan in patients with breast cancer brain metastases and recurrent glioblastoma: a phase 0 window-of-opportunity trial
Source: Nat Commun. 2024 Aug 7;15:6707. doi: 10.1038/s41467-024-50558-9 (PMC11306739; doi:10.1038/s41467-024-50558-9)
Supplement: Supplementary file 2 — Reporting Summary [file 41467_2024_50558_MOESM2_ESM.pdf]

nature portfolio

Corresponding author(s): Andrew Brenner

Last updated by author(s): May 13, 2024

Reporting Summary

Nature Portfolio wishes to improve the reproducibility of the work that we publish. This form provides structure for consistency and transparency in reporting. For further information on Nature Portfolio policies, see our Editorial Policies and the Editorial Policy Checklist.

Statistics

For all statistical analyses, confirm that the following items are present in the figure legend, table legend, main text, or Methods section.

n/a	Confirmed	
☐	☒	The exact sample size (*n*) for each experimental group/condition, given as a discrete number and unit of measurement
☒	☐	A statement on whether measurements were taken from distinct samples or whether the same sample was measured repeatedly
☐	☒	The statistical test(s) used AND whether they are one- or two-sided<br>*Only common tests should be described solely by name; describe more complex techniques in the Methods section.*
☒	☐	A description of all covariates tested
☐	☒	A description of any assumptions or corrections, such as tests of normality and adjustment for multiple comparisons
☐	☒	A full description of the statistical parameters including central tendency (e.g. means) or other basic estimates (e.g. regression coefficient) AND variation (e.g. standard deviation) or associated estimates of uncertainty (e.g. confidence intervals)
☐	☒	For null hypothesis testing, the test statistic (e.g. *F*, *t*, *r*) with confidence intervals, effect sizes, degrees of freedom and *P* value noted<br>*Give P values as exact values whenever suitable.*
☒	☐	For Bayesian analysis, information on the choice of priors and Markov chain Monte Carlo settings
☒	☐	For hierarchical and complex designs, identification of the appropriate level for tests and full reporting of outcomes
☒	☐	Estimates of effect sizes (e.g. Cohen's *d*, Pearson's *r*), indicating how they were calculated

Our web collection on statistics for biologists contains articles on many of the points above.

Software and code

Policy information about availability of computer code

Data collection	REDCap (multiple versions up to 13.7.31) was used for data collection and monitoring throughout.
Data analysis	SAS Version 9.3 for Windows (SAS Institute, Cary, North Carolina) was used throughout. Microsoft 365 (multiple versions up to v2403) with Word, Excel and PowerPoint were used for analysis and manuscript drafting.

For manuscripts utilizing custom algorithms or software that are central to the research but not yet described in published literature, software must be made available to editors and reviewers. We strongly encourage code deposition in a community repository (e.g. GitHub). See the Nature Portfolio guidelines for submitting code & software for further information.

Data

Policy information about availability of data

All manuscripts must include a data availability statement. This statement should provide the following information, where applicable:

- Accession codes, unique identifiers, or web links for publicly available datasets
- A description of any restrictions on data availability
- For clinical datasets or third party data, please ensure that the statement adheres to our policy

In order to protect potential indirect identifiers while also supporting scientific endeavors, the data generated in this study are available upon appropriate request from the corresponding author. Requests can be made for preclinical and deidentified clinical data. Email is the preferred mode of contact and requests should be made from publication and up until 36 months following publication. Requests for non-commercial analysis should be made by researchers and include sound justification such as for use in meta-analysis. Signed data access agreements may be required.

Research involving human participants, their data, or biological material

Policy information about studies with human participants or human data. See also policy information about sex, gender (identity/presentation), and sexual orientation and race, ethnicity and racism.

Reporting on sex and gender	Breast cancer has a large female predominance and all of the patients in the breast cancer cohort self-identified as female. Due to biological differences including the influence of hormones, only female mice were used in the xenograft model of breast cancer. Glioblastoma has a male predominance and 75% of our rGBM cohort self-identified as male. No formal subgroup analysis by sex or gender was conducted due to limited sample size.
Reporting on race, ethnicity, or other socially relevant groupings	Due to the nature of our patient catchment area, 40% of patients self-identified as Hispanic or Latino. No formal subgroup analysis by race or ethnicity was conducted due to limited sample size.
Population characteristics	No known covariates were examined of controlled for in this study due to limited sample size.
Recruitment	Clinical trials were offered during clinic and posted on Clinicaltrials.gov
Ethics oversight	UTHSA IRB and IACUC

Note that full information on the approval of the study protocol must also be provided in the manuscript.

Field-specific reporting

Please select the one below that is the best fit for your research. If you are not sure, read the appropriate sections before making your selection.

☒ Life sciences ☐ Behavioural & social sciences ☐ Ecological, evolutionary & environmental sciences

For a reference copy of the document with all sections, see nature.com/documents/nr-reporting-summary-flat.pdf

Life sciences study design

All studies must disclose on these points even when the disclosure is negative.

Sample size	25 patients. Given the paucity of available data regarding ADC uptake in human tumors and the probable heterogeneity of this, no formal sample size calculations were performed. Accordingly, sample size for this phase 0 window-of-opportunity study was determined primarily by logistics and feasibility.
Data exclusions	Data exclusion, such as for a patient who had radiographic 'recurrence' but on at time of surgery was found to actually have radiation effect/ necrosis, is detailed in Demographics Supplementary and the CONSORT flow diagram.
Replication	This was a phase 0 study. We did not seek to replicate our initial results or that of another study.
Randomization	No randomization was undertaken for this phase 0 study.
Blinding	No blinding was undertaken due to risks of surgery and study design.

Reporting for specific materials, systems and methods

We require information from authors about some types of materials, experimental systems and methods used in many studies. Here, indicate whether each material, system or method listed is relevant to your study. If you are not sure if a list item applies to your research, read the appropriate section before selecting a response.

Materials & experimental systems

n/a	Involved in the study
☐	☒ Antibodies
☐	☒ Eukaryotic cell lines
☒	☐ Palaeontology and archaeology
☐	☒ Animals and other organisms
☐	☒ Clinical data
☒	☐ Dual use research of concern
☒	☐ Plants

Methods

n/a	Involved in the study
☒	☐ ChIP-seq
☒	☐ Flow cytometry
☐	☒ MRI-based neuroimaging

Antibodies

Antibodies used	Sacituzumab was provided by Gilead for clinical trial treatment. IHC antibodies were Polyclonal goat anti-Trop-2 antibody (R&D Systems, Catalog: AF650, Clone: not provided, Lot: CIE0319091) at a concentration of 5 µg/mL (Dilution 1 to 40); Carbonic anhydrase IX (CAIX) using a kit (WILEX Oncogene Science, CA IX IHC Kit, Catalog: 06490035, Clone: not provided, Lot: 776391A) and an antibody against Phospho-Histone γH2AX ([Ser139][20E3], Cell Signaling, Catalog: 9718S, Clone: not provided, Lot: 21, Dilution: 1 to 100).
Validation	None. No primary antibodies were used, only commercially available antibodies.

Eukaryotic cell lines

Policy information about cell lines and Sex and Gender in Research

Cell line source(s)	Triple negative breast cancer cell line (MDA-MB-468-GFP-Luc). Cell line was sourced from ATCC HTB-134.
Authentication	Cells were not authenticated.
Mycoplasma contamination	Cells were tested for mycoplasma contamination.
Commonly misidentified lines (See ICLAC register)	MDA-MB-468 does not appear in misidentified cell lines in the ICLAC register (version 13).

Animals and other research organisms

Policy information about studies involving animals; ARRIVE guidelines recommended for reporting animal research, and Sex and Gender in Research

Laboratory animals	Twenty SCID/NCr immunocompromised mice were inoculated intracranially with a 2 x 106 count of a triple negative breast cancer cell line (MDA-MB-468-GFP-Luc). All mice were 6 weeks at time of inoculation. Mice were housed in a dedicated pathogen-free cages with a cycle of 12 hours light and 12 dark and 22°C and 50% humidity. All mice were 6 weeks at time of inoculation.
Wild animals	None
Reporting on sex	Only female mice were used as this was a model of breast cancer metastasis and men account for less than 1 percent of breast cancer incidents in humans.
Field-collected samples	None
Ethics oversight	All animal experiments were performed using a UTHSCA IACUC approved protocol and according to all relevant ethical regulations.

Note that full information on the approval of the study protocol must also be provided in the manuscript.

Clinical data

Policy information about clinical studies

All manuscripts should comply with the ICMJEguidelines for publication of clinical research and a completedCONSORT checklist must be included with all submissions.

Clinical trial registration	Trial (NCT03995706) enrolled at Clinical Trials.gov as Neuro/Sacituzumab Govitecan/Breast Brain Metastasis/Glioblastoma/Ph 0: https://clinicaltrials.gov/study/NCT03995706?cond=NCT03995706
Study protocol	Protocol to be uploaded in supplemental upon publication.
Data collection	RedCAP was used for clinical data as per Institutional policy throughout the study period. Patient enrollment took place from 8/16/19 to 10/22/20 at the Mays Cancer Center. Mays Cancer Center is the only NCI-Designated cancer center in South Texas and part of the University of Texas Health Science Center at San Antonio (UTHSCSA). Data collection continued through 9/2023. Length of follow up was 1 year.

Outcomes	The primary outcome was SN-38 concentration in surgical tissue samples. The primary endpoint was to determine intracranial concentrations of SN-38 as well as those of serum and CSF by UHPLC-MS. Secondary endpoints were measuring the PFS and OS for these patients from first post-surgical treatment, as determined by RANO and RECIST as well as to assess safety in this population as determined by CTCAE. While not statistically powered, the exploratory analysis of Trop-2, CAIX and γH2AX expression and correlation was pre-specified and quantified using IHC.

Plants

Seed stocks	None.
Novel plant genotypes	None.
Authentication	None.

Magnetic resonance imaging

Experimental design

Design type	Clinical Use Only.
Design specifications	As per clinical Use.
Behavioral performance measures	None.

Acquisition

Imaging type(s)	MRI scans were performed on 3T MRI scanners (Philips, GE, and Siemens).
Field strength	3T
Sequence & imaging parameters	Each session consisted of 3D pre- and post-contrast T1 weighted images, FLAIR (fluid-attenuated inversion recovery), diffusion weighted images, and Dynamic Susceptibility Contrast (DSC). T1 pre-contrast, FLAIR images were acquired before administration of contrast agent, dynamic contrast enhancement (DCE) and diffusion weighted images were acquired after. For DCE, injection took place after 10 baseline frames were obtained. The second injection was for DSC perfusion MRI and T1 post-contrast images.
Area of acquisition	Per radiologist and technician discretion.

Diffusion MRI ☐ Used ☐ Not used

Preprocessing

Preprocessing software	Per radiologist and technician discretion.
Normalization	Per radiologist and technician discretion.
Normalization template	Per radiologist and technician discretion.
Noise and artifact removal	Per radiologist and technician discretion.
Volume censoring	Per radiologist and technician discretion.

Statistical modeling & inference

Model type and settings	Per radiologist and technician discretion.
Effect(s) tested	Per radiologist and technician discretion.

Specify type of analysis: ☐ Whole brain ☐ ROI-based ☐ Both

Statistic type for inference (See Eklund et al. 2016)	Per radiologist and technician discretion.

Correction	Per radiologist and technician discretion.

Models & analysis

n/a	Involved in the study
☒	☐ Functional and/or effective connectivity
☒	☐ Graph analysis
☒	☐ Multivariate modeling or predictive analysis
